# Transcriptome of fetal cortex of tree shrew underlying the emergence of outer subventricular zone

**DOI:** 10.1038/s42003-026-10802-y

**Published:** 2026-09-14

**Authors:** Ting Hu, Yangxuan Wen, Yulian Tan, Yifan Kong, Ling Yuan, Soojin V. Yi, Lei Shi

**Affiliations:** 1 State Key Laboratory of Genetic Evolution & Animal Models, Kunming Institute of Zoology, Chinese Academy of Sciences, Kunming, Yunnan PR China; 2 Yunnan Key Laboratory of Animal Models and Human Disease Mechanisms, Kunming Institute of Zoology, Chinese Academy of Sciences, Kunming, Yunnan PR China; 3 Kunming College of Life Science, University of Chinese Academy of Sciences, Kunming, Yunnan PR China; 4 Center for Medical Genetics, Hunan Key Laboratory of Medical Genetics, MOE Key Lab of Rare Pediatric Diseases, School of Life Sciences, Central South University, Changsha, Hunan PR China; 5 Department of Ecology, Evolution, and Marine Biology, University of California, Santa Barbara, CA USA; 6 Department of Molecular, Cellular and Developmental Biology, University of California, Santa Barbara, CA USA; 7 Neuroscience Research Institute, University of California, Santa Barbara, CA USA; 8 National Resource Center for Non-Human Primates, Kunming Primate Research Center, and National Research Facility for Phenotypic & Genetic Analysis of Model Animals (Primate Facility), Kunming Institute of Zoology, Chinese Academy of Sciences, Kunming, Yunnan PR China

**Keywords:** Evolutionary developmental biology, Developmental neurogenesis

## Abstract

A defining feature of primates is the expansion and increased complexity of the cerebral cortex, processes that are largely associated with evolutionary adaptations of the neural progenitor-enriched outer subventricular zone (OSVZ). Yet, the genetic mechanisms underlying the emergence of the primate OSVZ remain unknown. The tree shrew is a closer outgroup to primates than rodents, its brain exhibits an intermediate phenotype; while being *lissencephalic*, it nonetheless possesses an OSVZ. Here, we applied laser microdissection technology to conduct comprehensive gene expression analyses of different cortical layers in the tree shrew brain across three key developmental stages. We show that cell cycle-related genes are already involved in the tree shrew OSVZ. In contrast, extracellular matrix-associated pathways appear to be unique to the primate OSVZ. Furthermore, overexpression of *ZNF664* significantly increased intermediate progenitor proliferation in the mouse subventricular zone. Together, these results reveal shared molecular mechanisms underlying OSVZ development in primates and tree shrews, providing insights into the origin of primate cortical complexity.

## Abstract (web summary)

The authors generated gene expression profiles for each cortical layer of the tree shrew, especially of OSVZ. Cross-species comparative analyses revealed that cell cycle- and ECM-associated pathways are shared between primates and tree shrews. Zinc finger proteins were identified as unrecognized regulators of OSVZ evolution

## Introduction

The increase in brain complexity and cognitive abilities has distinguished humans from other non-human primates^1,2^. Numerous comparative analyses between humans and non-human primates have highlighted that genetic changes have driven the evolution and development of human cortical phenotypes^2–4^, including human accelerated regions^5^, sequence changes in protein-coding^6,7^ and noncoding genes^8,9^, segmental duplications and other structural variations^10,11^, alterations in epigenetic regulation^12,13^, and changes in gene expression^14–16^. It is important to note that many human-specific traits originated in ancestral lineages, such as those leading to primates^17–19^. However, the genetic changes contributing to the emergence and evolution of primate phenotypes, particularly the complexity of primate brains, remain poorly understood.

Brain evolution is tightly linked to cortical development processes^19–21^, which ultimately depend on the behavior of neural progenitor cells (NPCs)^22,23^. NPCs are distributed across two germinal zones: the ventricular zone (VZ) and the subventricular zone (SVZ), which lies basal to the VZ. Apical progenitors (APs) are primarily located in the VZ, where they span the entire cortical wall during neurogenesis, whereas basal progenitors (BPs) accumulate in the SVZ^24^. BPs are derived from APs and mainly comprise intermediate progenitor cells (IPCs) and basal radial glial cells (bRGCs).

The mouse fetal cortex contains a VZ and a comparatively thinner SVZ. In primates, the SVZ is markedly expanded and further subdivided into the inner SVZ (ISVZ) and the outer SVZ (OSVZ), which are demarcated by a thin fiber layer^25–28^. While the ISVZ is comparable to the mouse SVZ^29^, the OSVZ is absent in the mouse cortex^25^. This structural divergence is accompanied by distinct progenitor cell populations. In mice, IPCs are the predominant progenitor type in the SVZ, whereas in primates the OSVZ is primarily populated by bRGCs, also known as outer radial glial cells (oRGCs). During primate brain evolution, bRGCs acquired enhanced self-renewal and proliferative capacities^30,31^, thereby contributing to the substantial expansion of the OSVZ^28,32^. These processes are thought to play crucial roles in driving the expansion and increased complexity of the human cortex^22,29,33,34^. However, the molecular mechanisms underlying the origin and evolution of the OSVZ remain largely unresolved.

**Fig. 1:**
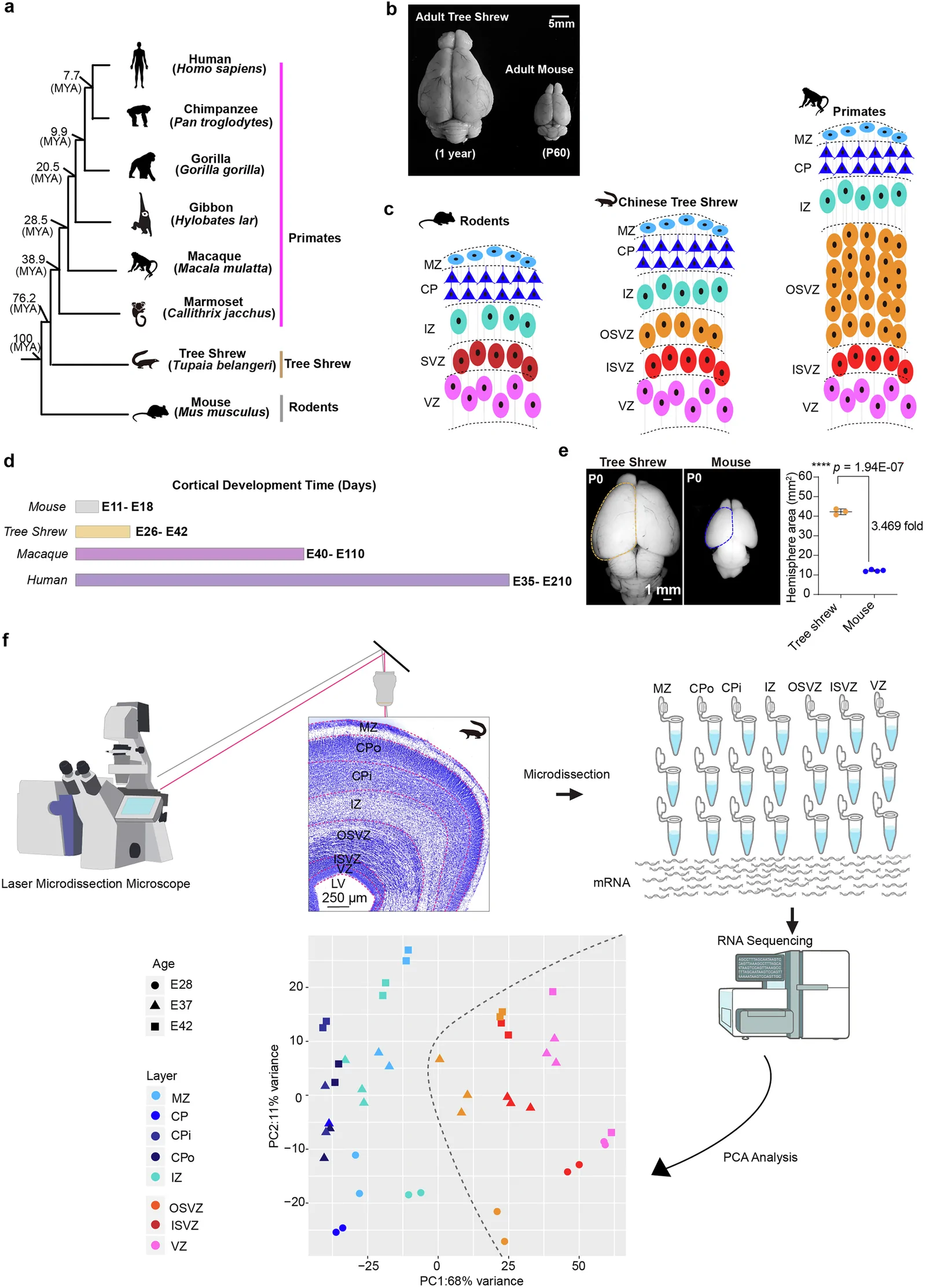
Laser microdissection of tree shrew cortical laminae. **a** A schematic diagram illustrating the phylogenetic relationships among rodents, the Chinese tree shrew, and primates. Numbers indicate divergence times in millions of years. **b** Representative brain images of adult tree shrews and mice. **c** A schematic diagram showing cortical layer organization in rodents, tree shrews, and primates. **d** A schematic diagram displaying the developmental time of cerebral cortex among rodents, the Chinese tree shrew, macaque and human. Note that the data of developmental time for each species was assessed from published studies^40,43–45^. **e** Quantification of cortical size in the tree shrew and mice at P0. (tree shrew, *n* = 3; mouse, *n* = 4, mea*n* ± SD, **** represents *p* < 0.0001, two-tailed, unpaired *t*-test). **f** (Top) A schematic overview of the microdissection workflow and a representative Nissl-stained image of the E37 Chinese tree shrew cortex, showing eight dissected layers from the marginal zone to the ventricular zone. (Bottom) PCA of frontal cortical layers from Chinese tree shrews at E28, E37, and E42 based on genome-wide gene expression levels.

The Chinese tree shrew (*Tupaia belangeri chinensis*) is a closer outgroup to primates than rodents^35–38^ (Fig. 1a). It has a higher brain-to-body mass ratio than the mouse, and its adult brain area is approximately threefold larger than that of an adult mouse (Fig. 1b). Intriguingly, the tree shrew and other clades including carnivores and ungulates exhibit an expanded OSVZ during cortical development^39–41^, suggesting that SVZ expansion preceded the emergence of primates. Despite this feature, tree shrews are lissencephalic, possessing a smooth cortex with a gyrification index (GI) of approximately 1.06^22^ (Fig. 1b). Together, these features position the tree shrew as a transitional model that captures early evolutionary changes associated with primate brain evolution.

In this study, we successfully dissected cortical layers and generated genome-wide gene expression maps during tree shrew development, to investigate molecular features linked to OSVZ evolution. Our results suggest that cell-cycle processes were already functionally involved in the OSVZ prior to primate evolution, whereas ECM-associated genes may be primarily responsible for OSVZ expansion in primates. Moreover, we discovered that zinc finger proteins may also contribute to OSVZ evolution. Remarkably, overexpression of *ZNF664* significantly increased the number of progenitors in the mouse SVZ. In summary, the tree shrew can serve as a novel animal model for studying primate brain evolution and development.

## Results

### Transcriptome maps of the cortical lamina of Chinese tree shrews

Unlike rodents (e.g., mice), the tree shrew SVZ is subdivided into the ISVZ and OSVZ, closely resembling primate cortical organization (Fig. 1c). However, the molecular and transcriptional programs driving this developmental and evolutionary divergence remain largely unexplored. The peak of tree shrew OSVZ development occurs around embryonic day 37 (E37), within a gestation period of 41-45 days^42^. The developmental time the cerebral cortex in tree shrew can persist about ~17 days^40^, which is twice as long as in mouse (~8 days), but much shorter than primates^43–45^ (macaque ~70 days; human ~175 days) (Fig. 1d). Accordingly, the brain size of tree shrew is about 3.46 fold of mouse at postnatal 0 days (P0) (Fig. 1e). To characterize the molecular features associated with cortical layer development, we collected embryonic tree shrew brains at three key neurogenic stages: E28, representing an early stage; E37, corresponding to the mid-stage and peak of OSVZ development; and E42, representing a late stage of cortical development (Fig. 1f and Supplementary Data 1).

We used laser capture microdissection of fetal cortices to generate transcriptome data from the marginal zone (MZ), cortical plate (CP), intermediate zone (IZ), OSVZ, ISVZ, and VZ across these three developmental stages (Fig. 1f, top panel). Principal component analysis (PCA) demonstrated that the first principal component (PC1) separated neuron-enriched layers, including the MZ, which contains Cajal-Retzius cells, transient glutamatergic neurons; the CP, enriched for mature neurons; and the IZ, populated by migrating neurons, from progenitor-enriched germinal zone (GZ) layers, comprising the VZ, ISVZ, and OSVZ. Within the GZs, the VZ, which harbors APs, was distinct from the ISVZ and OSVZ, both of which were enriched in BPs (Fig. 1f, bottom panel). In contrast, PC2 largely separated developmental stages. Separate PCA analyses of each developmental stage confirmed the pattern observed in the combined PCA, in which PC1 separated neuron-rich layers from progenitor-rich layers (Supplementary Fig. 1a–c, left panel). The expression of marker genes further corroborated cortical organization. Specifically, the NPC marker *SOX2*, the neuronal markers *SATB2*, the migrating neuron marker *DAB1*, and the Cajal-Retzius cell marker *RELN* were highly expressed in the GZ, CP, IZ, and MZ, respectively (Supplementary Fig. 1a–c, right panel).

Therefore, the transcriptional signatures of the dissected layers reflect their temporal and spatial positions within the embryonic tree shrew cortex.

### Layer- and stage-specific gene expression in fetal cortical laminae

**Fig. 2:**
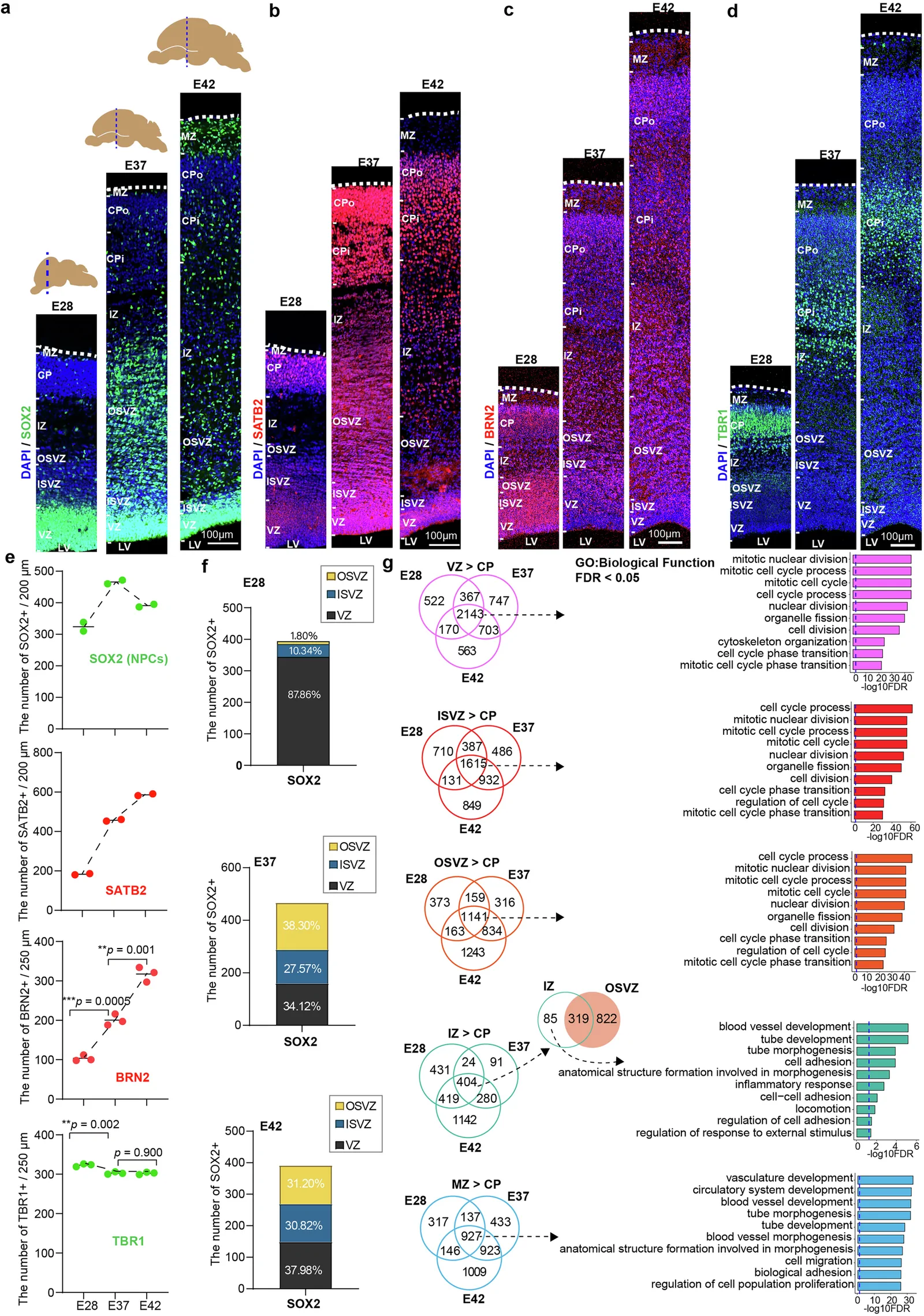
Generation of cortical lamina transcriptome profiles during tree shrew development. **a–d** Immunofluorescence staining of cortical sections for the upper-layer, deep-layer, and AP markers *SATB2*, *BRN2, TBR1*, and *SOX2*, respectively, at E28, E37, and E42 in Chinese tree shrews. **e** Quantification of the distribution patterns of *SOX2*-, *SATB2*-, *BRN2*, and *TBR1*-positive cells during cortical development. (SOX2 and SATB2:E28, *n* = 2, E37, *n* = 2, E42, *n* = 2; BRN2 and TBR1:E28, *n* = 3, E37, *n* = 3, E42, *n* = 3, mean, * represents *p* < 0.05, ** represents *p* < 0.01, *** represents *p* < 0.001, two-tailed, unpaired *t*-test). **f** Quantification of *SOX2*^*+*^ cell numbers and proportions in the VZ, ISVZ, and OSVZ at E37 and E42. Two biological replicates were analyzed at each time point. **g** Identification and GO analysis of genes consistently highly expressed in each cortical layer across three developmental stages. Note that IZ-expressed genes were filtered by OSVZ-expressed genes, to avoid any potential issues due to the proximity between IZ and OSVZ.

We further utilized immunostaining to examine layer-specific transcriptional programs. Using excitatory neuron marker SATB2^+^, upper-layer (L2-L4) BRN2^+^ and deeper-layer TBR1^+^ neuron markers, we observed that, during neurogenesis, SATB2^+^ neurons became enriched in the outer cortical plate (CPo). BRN2^+^ neurons were still in migration, dispersing across the cortical plate, whereas TBR1^+^ neurons were restricted to the inner cortical plate (Fig. 2a–d). Quantification analysis suggested that TBR1^+^ neurons first decreased from E28 to E37, and then remained at a comparable level from E37 to E42 (Fig. 2e), while SATB2^+^ and BRN2^+^ neurons continued to increase along neurogenesis (Fig. 2e). SOX2^+^ progenitor cells in the VZ, on the other hand, increased markedly from E28, reached a peak at E37, and subsequently dispersed along the entire cortical wall by E42 (Fig. 2e). In addition, at E37, the proportion of SOX2^+^ cells was highest in the OSVZ (Fig. 2e, OSVZ-38.3% vs ISVZ-27.57% and VZ-34.12%), whereas at E42, most SOX2+ cells were located in the VZ (Fig. 2f, VZ-37.98% vs OSVZ-31.2% and ISVZ-30.82%).

As we observed some SOX2-positive cells in the cortical plate (Fig. 2a), we further examined whether this was due to biological differences, antibody specificity or other technical factors. Using another SOX2-antibody (CST, Cell Signaling Technology, 4900 T) in parallel with the previously used SOX2-antibody (Invitrogen, 53-9811-82), we performed SOX2-immunostaining again at E28, E37 and E42 (Supplementary Fig. 2a). Overall, we observed the trend regarding SOX2^+^ progenitor cells, reaching the peak at E37 (Fig. 2e and Supplementary Fig. 2b), although the number of SOX2^+^ cells was slightly higher from the Invitrogen antibody compared to that from CST (Supplementary Fig. 2b). Moreover, for both antibodies, the proportions of SOX2^+^ progenitors in the CP were much lower than those VZ, ISVZ and OSVZ (Supplementary Fig. 2c). We further examined images from previous studies and also observed the presence of low numbers of SOX2+ cells in the embryonic cortical plates^46–48^. These results indicated that the presence of SOX2^+^ cells in the cortical plate of tree shrew may represent a true biological pattern during embryonic development, although the possibility of false positives cannot be completely excluded. Nevertheless, these findings suggest that NPC proliferation processes were reaching the peak in the OSVZ around E37, while NPC differentiation processes may have already initiated at earlier stages.

We then examined differential gene expression (DEG) between cortical layers (Supplementary Fig. 3 and Supplementary Data 2–4). Focusing on the molecular features of progenitor-rich layers, we identified genes that were differentially upregulated in these layers compared with the CP (Fig. 2g). We identified 2143, 1615, and 1141 genes that were consistently and significantly upregulated in the VZ, ISVZ, and OSVZ, respectively, relative to the CP across all developmental stages. Gene ontology (GO) analysis revealed that these genes were primarily enriched in cell cycle-related processes (Fig. 2g and Supplementary Data 5). IZ is adjacent to the OSVZ (Fig. 2a–d), although we identified 404 genes were consistently highly expressed (Fig. 2g and Supplementary Data 5), we further intersected these genes with 1141 OSVZ expressed genes, and identified 85 genes as IZ-expressed gene with highly confidence, enriched in tube development, cell-cell adhesion, and related processes, consistent with its role in neuronal migration (Fig. 2g and Supplementary Data 5). In the MZ, 927 genes were consistently highly expressed and enriched in vascular development, tube development, and cell migration (Fig. 2g and Supplementary Data 5). Developmental stage-specific DEG and GO analyses yielded results consistent with these overall patterns (Fig. 2g and Supplementary Fig. 4a–c).

### Genes exhibiting linear changes of expression in CP and IZ

**Fig. 3:**
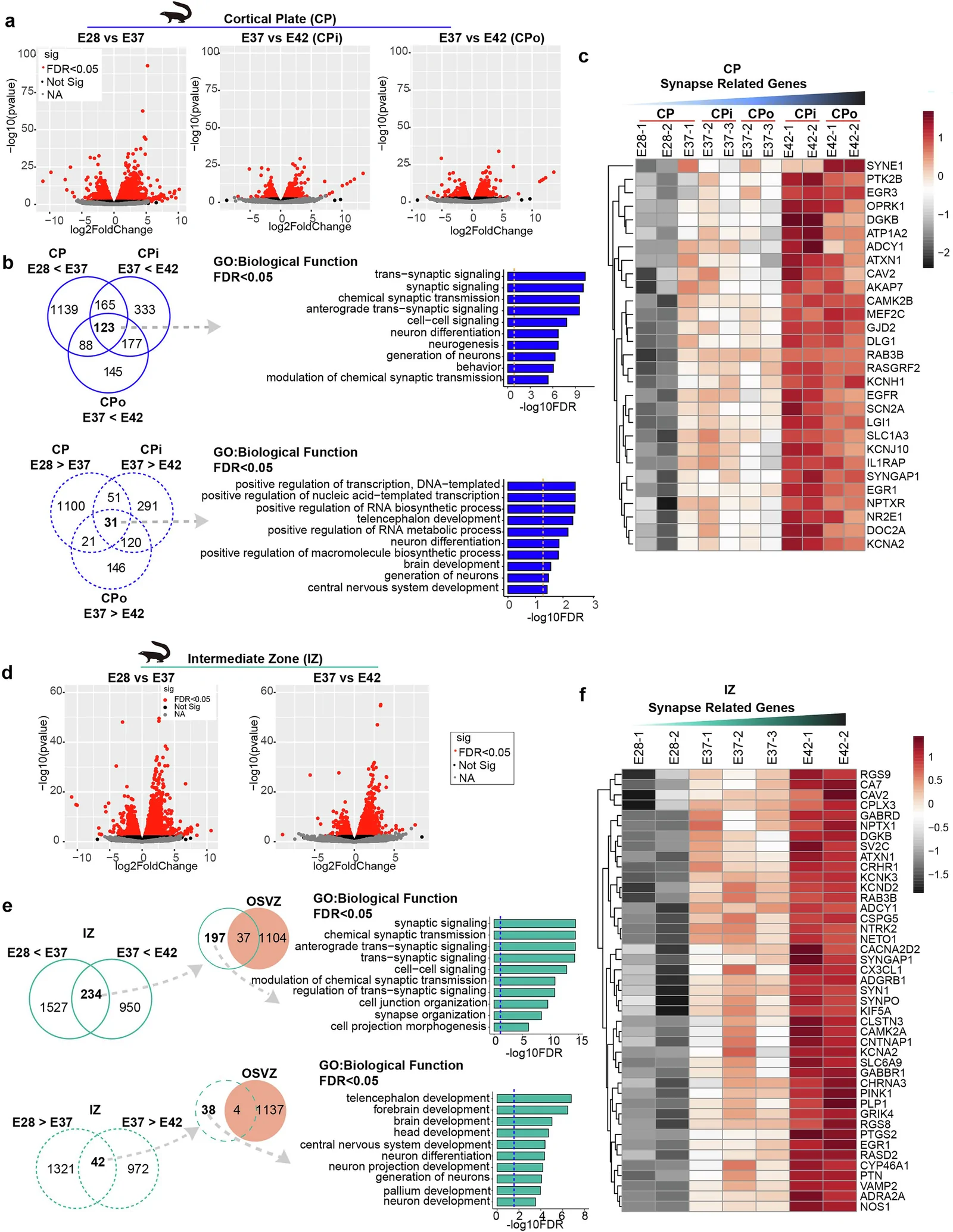
Synapse-related genes are progressively upregulated in the cortical plate (CP) and intermediate zone (IZ) during tree shrew brain development. **a** Volcano plots showing DEGs between E28 and E37, and between E37 and E42, in the CP, inner CP, and outer CP. **b** Venn diagram plots showing genes with consistent expression changes during cortical development. (Top) A total of 123 genes exhibited consistent increases in expression and were predominantly enriched in synaptic signaling-associated pathways. (Bottom) Thirty-one genes associated with neurogenesis showed decreased expression during cortical development. Note that the broken line marks the cutoff at -log10(FDR) = 1.301. **c** Hierarchical clustering illustrating the gradual upregulation of synapse-related genes during cortical plate development in the tree shrew. **d** Volcano plots showing DEGs between E28 and E37, and between E37 and E42, in the intermediate zone. **e** Venn diagram plots showing genes with consistent expression changes in the intermediate zone. (Top) A total of 234 genes exhibited consistent increases in expression and were predominantly enriched in synaptic signaling-associated pathways. (Bottom) Forty-two genes associated with neurogenesis showed decrease of expression during cortical development. Note that the broken line marks the cutoff at -log10(FDR) = 1.301, and IZ-expressed genes were further curated by filtering out OSVZ-expressed genes. **f** Hierarchical clustering illustrating the gradual upregulation of synapse-related genes during intermediate zone development in the tree shrew.

During cortical neurogenesis, newly born neurons migrate along radial glial cell (RGC) scaffolds toward the MZ and gradually mature into distinct cortical layers within the CP. To characterize transcriptional changes accompanying neuronal maturation, we analyzed gene expression dynamics in neuron-rich layers CP, IZ, and MZ, corresponding to mature, migrating, and transient neurons, respectively. We aimed to identify genes that exhibit linear increases or decreases in expression during development, reasoning that such genes may play important roles in the neuronal maturation process (Fig. 3a, d and Supplementary Data 6). We identified 123 and 234 genes whose expression increased linearly during development in the CP and IZ, respectively. These genes were enriched in synapse-related signaling pathways (Fig. 3b, e, top panel, and Supplementary Data 6), which are known to play key roles in neuronal maturation. Consistently, hierarchical clustering analysis based on synapse-associated DEGs in the CP and IZ revealed clear linear increases in expression across developmental stages (Fig. 3c, f). Notably, several of these genes are implicated in human neurodevelopmental disorders. For example, *SCN2A* (Fig. 3c, CP), an autism spectrum disorder (ASD)-associated gene, plays a critical role in synaptic function and dendritic excitability^49^. Nitric oxide synthase 1 (*NOS1;* Fig. 3f, IZ) is associated with Fragile X syndrome and has been shown to be regulated in a species-dependent manner^50^.

In contrast, only 31 and 42 genes exhibited gradual decreases in expression in the CP and IZ, respectively. These genes were enriched in processes related to neuronal differentiation and neuronal development (Fig. 3b, e, bottom panel). Interestingly, in contrast to the reduced expression of neurogenesis-related genes observed in the CP and IZ, gliogenesis-associated genes exhibited linear increases in expression in the MZ, which is occupied by transient glutamatergic neurons, namely Cajal-Retzius (CR) cells (Supplementary Fig. 5a–c and Supplementary Data 7). These findings suggest that CR cells may play regulatory roles in early cortical organization in tree shrews.

Together, these data demonstrate that, as cortical neurons mature in the tree shrew, there is a coordinated transcriptional shift from neurogenesis programs toward synaptic and functional maturation programs.

### Differentially expressed genes in germinal zones

We next examined how transcriptional programs change over developmental time within progenitor-rich germinal zones (VZ) of the tree shrew cortex. While apical progenitors (APs) in the VZ showed largely stable expression profiles (Supplementary Fig. 5d, e and Supplementary Data 8), basal progenitors in the ISVZ and OSVZ exhibited distinct and dynamic transcriptional trajectories. Notably, the OSVZ retained active neurogenic and gliogenic programs at late developmental stages, distinguishing it from both the VZ and ISVZ.

In the ISVZ, 205 genes exhibited a linear increase in expression during development (Supplementary Fig. 5f, g, *n* = 205). These genes were associated with synaptic signaling pathways (Supplementary Fig. 5h), indicating that ISVZ progenitors progressively acquire neuronal expression features during cortical development. In contrast, genes showing linear decreases in expression were enriched for cell cycle and DNA repair processes (Supplementary Fig. 5g, bottom panel).

**Fig. 4:**
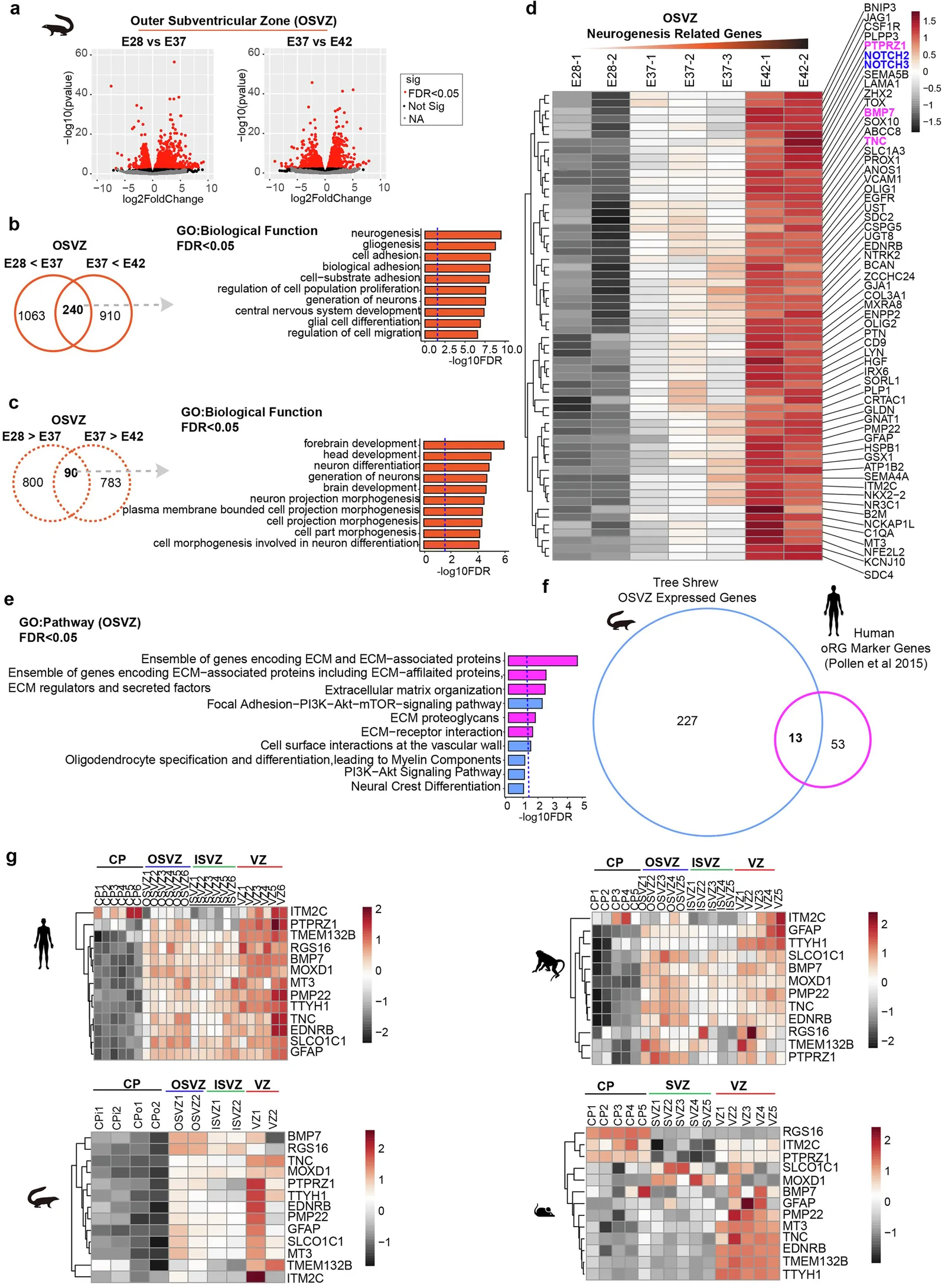
Neurogenesis-related genes are increased during tree shrew OSVZ development. **a** Volcano plots showing DEGs between E28 and E37, and between E37 and E42, in the outer subventricular zone. **b, c** Venn diagram plots illustrating genes with consistent expression changes during OSVZ development. **b** A total of 240 genes showed consistent increases in expression and were predominantly enriched for neurogenesis-related processes. **c** Ninety genes associated with morphogenetic signaling exhibited decreased expression during cortical development. Note that the broken line marks the cutoff at -log10(FDR) = 1.301. **d** Hierarchical clustering showing the gradual upregulation of neurogenesis-related genes during tree shrew OSVZ development. Pink and blue indicate genes associated with outer radial glial cells (oRGs) and cell proliferation, respectively. **e** GO pathway analysis of genes consistently upregulated during OSVZ development. Note that the broken line marks the cutoff at -log10(FDR) = 1.301, and ECM-associated pathways were highlighted in purple. **f** Venn diagram showing 13 genes shared with human outer oRG-enriched genes. **g** Hierarchical clustering showing the expression patterns of these 13 genes across mouse, tree shrew, macaque, and human cortices.

In the OSVZ (Fig. 4a and Supplementary Data 8), genes whose expression linearly decreased over development (*n* = 90) were primarily associated with brain development (Fig. 4c). By contrast, genes whose expression increased during OSVZ development (*n* = 240) were enriched in neurogenesis- and gliogenesis-related processes (Fig. 4b). In addition, the expression of *NOTCH2* and *NOTCH3* from NOTCH pathway^51^ gradually increased (Fig. 4d, blue color), indicating that OSVZ progenitors maintain proliferation-related programs at late stages of neurogenesis. Notably, several of these genes, including *PTPRZ1*, *TNC*, and *BMP7* (Fig. 4d, pink color), are well-established markers of outer radial glial cells in the primate OSVZ^52^.

Gene ontology analysis of genes whose expression linearly increased during OSVZ development further revealed enrichment for extracellular matrix (ECM)-related pathways and PI3K-Akt-mTOR signaling (Fig. 4e, ECM pathways highlighted in orange), consistent with mechanisms previously implicated in primate OSVZ expansion^53–56^. Moreover, 13 of these 240 genes overlapped with human OSVZ-enriched genes identified from micro-dissected germinal zones^52^ (Fig. 4f), suggesting the presence of conserved molecular features of the OSVZ that predate primate divergence. Analysis of their expression patterns across fetal cortical laminae in mouse, tree shrew, rhesus macaque, and human^57,58^ revealed that most of these genes (12 of 13, with the exception of *RGS16*) were highly expressed in the mouse VZ, but extended their expression into the SVZ of tree shrew, macaque, and human, where they remained highly expressed (Fig. 4g).

### Identification of OSVZ gene expression in tree shrews

**Fig. 5:**
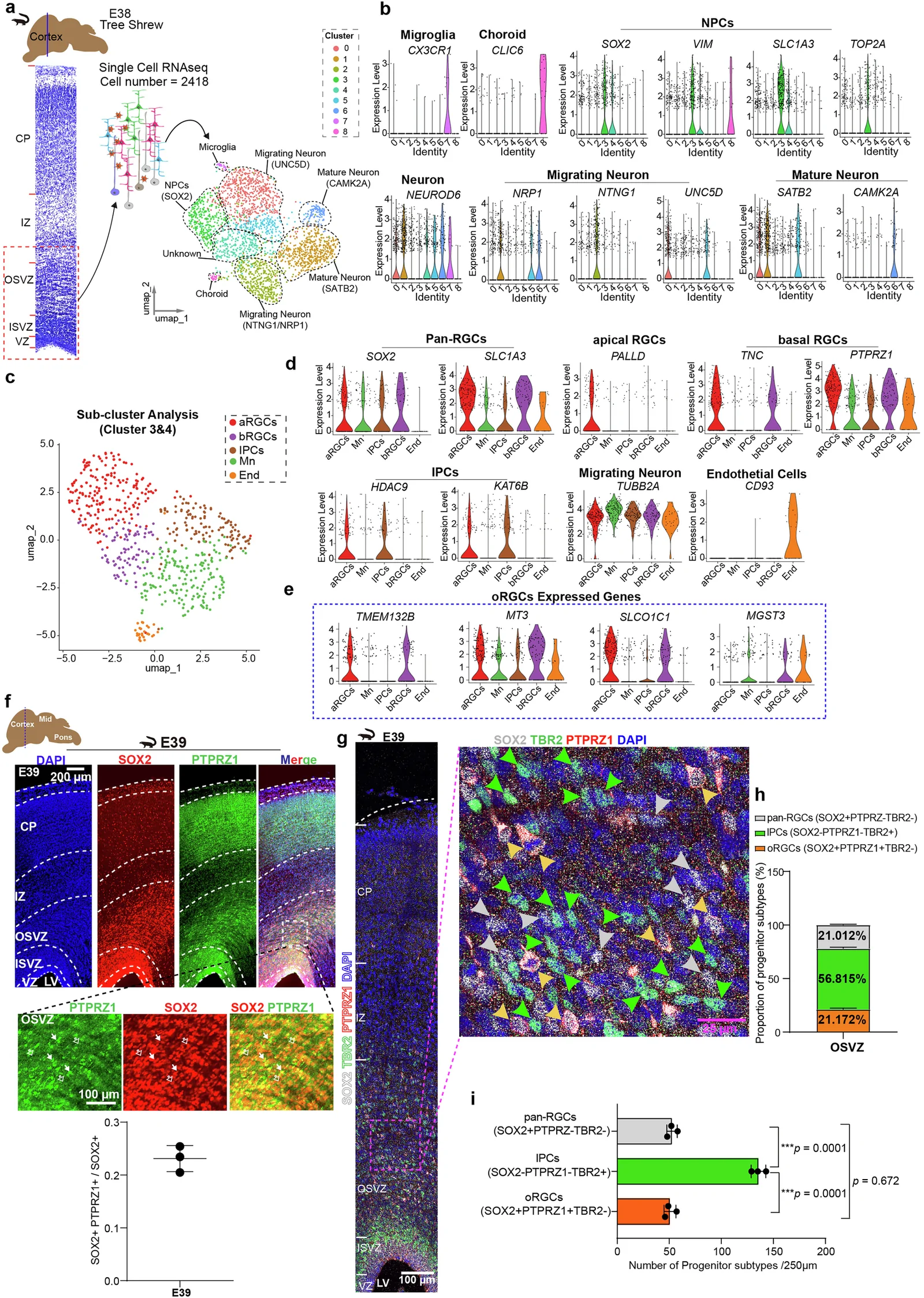
Single-cell analysis of the embryonic tree shrew cortex. **a** Schematic overview of the single-cell RNA sequencing workflow and uniform manifold approximation and projection (UMAP) plot showing cell types identified in the dissected germinal zone of the E38 tree shrew cortex. **b** Violin plots showing marker gene expression for microglia, choroid cells, RGCs, excitatory neurons, migrating neurons, and mature neurons in the single-cell dataset. **c** UMAP plot showing identified cell types from cluster 3 and cluster 4 by subcluster analysis. **d** Violin plots showing marker gene expression for pan-RGCs, apical RGCs, basal RGCs, IPCs, migrating neurons, and endothelial cells in the subclusters. **e** Violin plots showing expression of oRG-associated genes in the E38 embryonic tree shrew cortex. oRG-associated genes were highlighted in a dashed box. **f** Immunostaining and quantification of *PTPRZ1*^+^*SOX2*^+^ cells among *SOX2*^+^ cells in the E39 tree shrew cortex (tree shrew, *n* = 3; mea*n* ± SD). Solid arrows indicate *SOX2*^+^*PTPRZ1*^+^ double-positive cells, whereas open arrows indicate *SOX2*^+^ single-positive cells. **g** Immunostaining for pan-RGC marker SOX2, oRG marker PTPRZ1, and IPC marker TBR2 in the E39 tree shrew cortex. Green arrows indicate SOX2^-^PTPRZ1^-^TBR2^+^ cells, gray arrows indicate SOX2^+^PTPRZ1^-^TBR2^-^ cells whereas orange arrows indicate SOX2^+^PTPRZ1^+^TBR2^-^ cells. **h** The proportions of pan-RGC, oRGC, and IPCs in the E39 tree shrew OSVZ (*n* = 3 samples, standard deviation, SD). **i** The numbers of pan-RGC, oRGC, and IPCs in the E39 tree shrew OSVZ. (tree shrew, *n* = 3; mea*n* ± SD, *** represents *p* < 0.001, two-tailed, unpaired *t*-test).

To complement these observations, we performed single-cell RNA sequencing of the germinal zone, including the OSVZ, from embryonic tree shrew cortex at E38 (Fig. 5a). After quality control, 2418 single cells were retained for analysis. Uniform Manifold Approximation and Projection (UMAP) analysis indicated that there were at least 4 cell types, including radial glial cells (RGCs) representing progenitors (*SOX2*, *VIM*, *SLC1A3*, *TOP2A*), migrating neurons (*NRP1*, *NTNG1*, *UNC5D*), mature neurons (*SATB2*, *CAMK2A*), microglia (*CX3CR1*), and choroid cells (*CLIC6*) (Fig. 5a, b and Supplementary Data 9).

We further performed sub-cluster analysis with cluster 3 and 4, both of which exhibited much higher SOX2 expression than other clusters did (Fig. 5b–c). Different types of neural progenitors were identified by using *PALLD* as a marker for aRGCs^52^, *TNC* and *PTPRZ1* as markers for bRGCs^52^, *HDAC9 and KAT6B* for IPCs markers^59^, *TUBB2A* for migrating neuron^60^ (Fig. 5d). We found that the majority (45 of 66) of previously identified human OSVZ-specific genes^52^ were already expressed in the tree shrew ISVZ/OSVZ, although many were detected at relatively low expression levels (Supplementary Fig. 6a). Notably, six genes (*TNC*, *PTPRZ1*, *TMEM132B*, *MT3*, *SLCO1C1*, *MGST3*) were highly expressed in bRGCs, consistent with conserved OSVZ-associated progenitor identity (Fig. 5d–e). Notably, 4 genes (*EEPD1*, *FAM107A*, *BMP7*, *TKTL1*) were not detected in the tree shrew bRGCs (Supplementary Fig. 6b)

Furthermore, we performed immunostaining on the embryonic cortex using antibodies against PTPRZ1, a classical OSVZ marker^52^. We observed that PTPRZ1 was robustly expressed in the tree shrew VZ and ISVZ (Fig. 5f, top panel), while *PTPRZ1* showed selective expression in the OSVZ, with 23.11% of cells exhibiting SOX2^+^ PTPRZ1^+^ staining (Fig. 5f, bottom panel). To clarify the composition of progenitors in the tree shrew OSVZ, we performed triple-immunostaining using antibodies against SOX2, PTPRZ1, and IPCs marker TBR2 (Fig. 5g). Further quantification analysis suggested that in OSVZ, 56.815% of the progenitors belonged to IPCs (SOX2^-^PTPRZ1^-^TBR2^+^; Fig. 5g, green arrow), while pan-RGCs (SOX2^+^PTPRZ1^-^TBR2^-^; Fig. 5g, gray arrow) and oRGCs *(*SOX2^+^ PTPRZ1^+^TBR2^-^; Fig. 5g, orange arrow) are found at 20.172% and 21.012%, respectively (Fig. 5h). Consistently, in OSVZ, the number of IPCs was significantly higher than those of pan-RGCs and oRGCs, which were found in similar frequencies to each other (Fig. 5i).

Together, these results demonstrate that a shared molecular toolkit underlying OSVZ progenitor identity is conserved across tree shrews and primates, and potentially in other gyrencephalic mammals such as ungulates (pig, sheep) and carnivores (cat, ferret, dog).

### Comparative analysis of OSVZ gene expression between tree shrews and primates

**Fig. 6:**
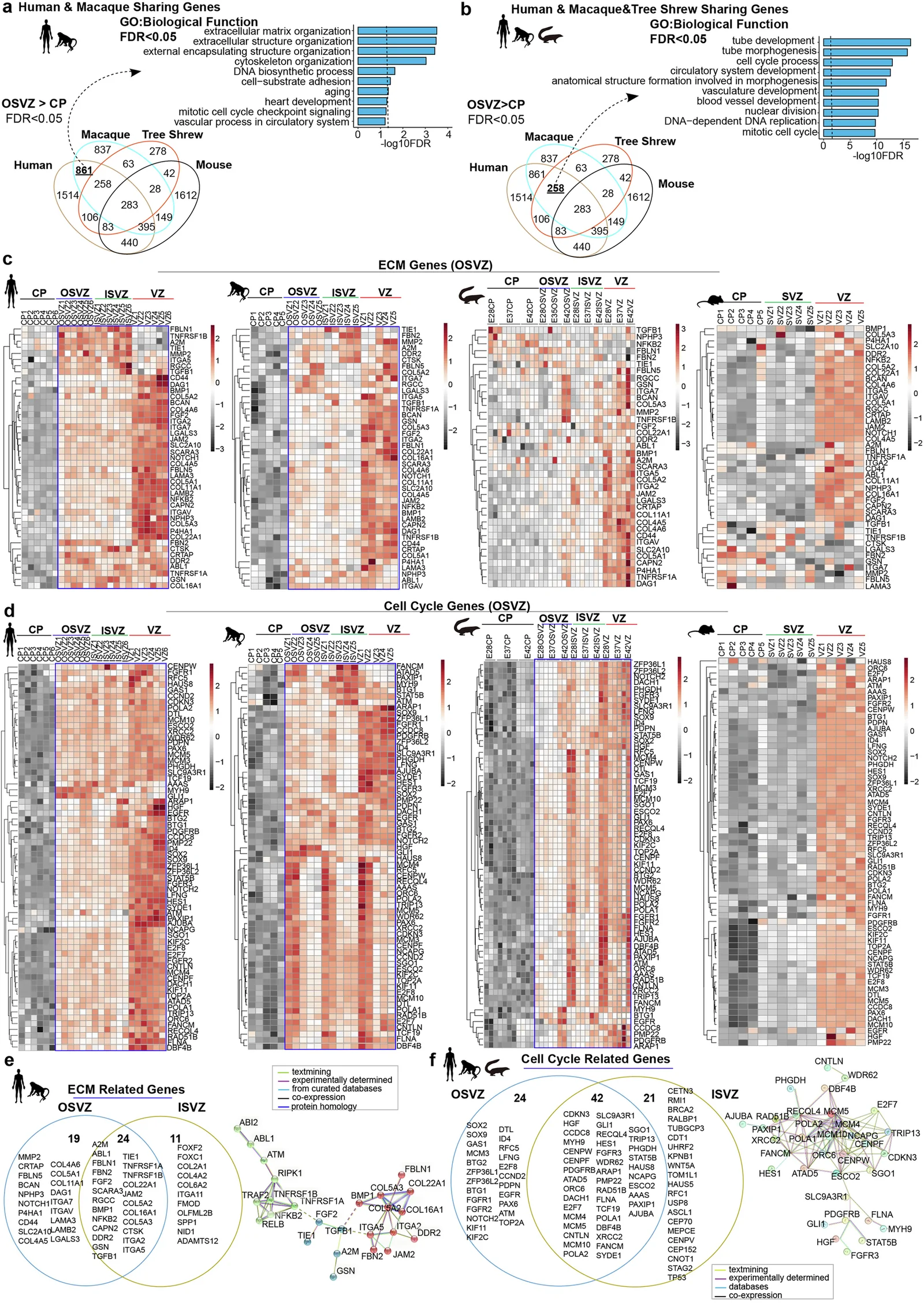
Comparative analysis of OSVZ-expressed genes among mouse, tree shrew, monkey, and human species. **a** GO analysis of OSVZ-expressed genes shared between human and macaque. Note that the broken line marks the cutoff at -log10(FDR) = 1.301. **b** GO analysis of OSVZ-expressed genes shared among human, macaque, and tree shrew. Note that the broken line marks the cutoff at -log10(FDR) = 1.301. **c** Hierarchical clustering showing expression pattern of ECM-associated genes in the OSVZ among mouse, tree shrew, monkey and human species. ECM-associated genes in human and monkey were highlighted in a dashed box. **d** Hierarchical clustering showing expression pattern of cell-cycle-associated genes in the OSVZ among mouse, tree shrew, monkey and human species. Cell-cycle-associated genes in human, monkey, and tree shrew were highlighted in a dashed box. **e** Venn diagram plots showing 24 ECM genes shared between the OSVZ and ISVZ in human and macaque, forming a protein-protein interaction network. **f** Venn diagram plots showing 42 cell cycle genes shared between the OSVZ and ISVZ in human, macaque, and tree shrew, forming a protein-protein interaction network.

To investigate molecular programs associated with OSVZ evolution, we performed comparative transcriptomic analyses of germinal zone layers across mouse, tree shrew, macaque, and human cortices. Using an approach consistent with that applied to the tree shrew dataset (Fig. 2f), we conducted differential gene expression analyses to identify genes more highly expressed in the OSVZ and ISVZ compared with the CP in human^57^, macaque^58^, and mouse^57^ (Supplementary Data 1). As an outgroup comparison, we identified genes more highly expressed in the mouse SVZ than in the mouse CP. Genes upregulated in the OSVZ and ISVZ relative to the CP in primates (human and macaque) (Fig. 6a and Supplementary Fig. 7a, 861 and 712 genes, respectively) were highly enriched for GO terms related to ECM organization and structure (Fig. 6a and Supplementary Fig. 7a). Consistently, expression patterns of ECM genes across the four species revealed increased expression in human and macaque germinal layers, whereas these genes were predominantly expressed in the VZ in tree shrew and mouse (Fig. 6c and Supplementary Fig. 7c, blue square).

We then identified 258 and 304 genes that were more highly expressed in the OSVZ and ISVZ, respectively, compared with the CP in human, macaque, and tree shrew (Fig. 6b and Supplementary Fig. 7b). Genes associated with cell cycle-related processes were overrepresented in the OSVZ (4/10) and ISVZ (8/10) (Fig. 6b and Supplementary Fig. 7b, pink). Across the four species, cell cycle genes in mouse displayed higher expression in the VZ than in other layers, particularly compared with the SVZ (Fig. 6d and Supplementary Fig. 7d). By contrast, in human, rhesus macaque, and tree shrew, cell cycle genes exhibited increased expression in the ISVZ and OSVZ (Fig. 6d and Supplementary Fig. 7d, blue square). Together, these results indicate that ECM and cell cycle-associated genes played important roles in the evolution of the OSVZ and ISVZ in primate brains. We further identified 24 ECM-genes and 42 cell cycle genes shared between the ISVZ and OSVZ that form a protein-protein interaction network (Fig. 6e, f), consistent with coordinated expression of these gene sets.

Together, these results highlight conserved and primate-enriched molecular programs in basal progenitor zones.

### *ZNF664* promotes proliferation of intermediate progenitor cells

Previous studies have recognized important roles for krüppel-associated box (KRAB) domain-containing zinc finger proteins (ZNFs) in primate neurodevelopment^61,62^. Our data provide a unique opportunity to explore the roles of ZNF genes in the evolution of the primate OSVZ, as multiple ZNF genes are selectively more highly expressed in the OSVZ and/or ISVZ than in the CP of human, macaque, and tree shrew cortices (Supplementary Fig. 8a, b).

**Fig. 7:**
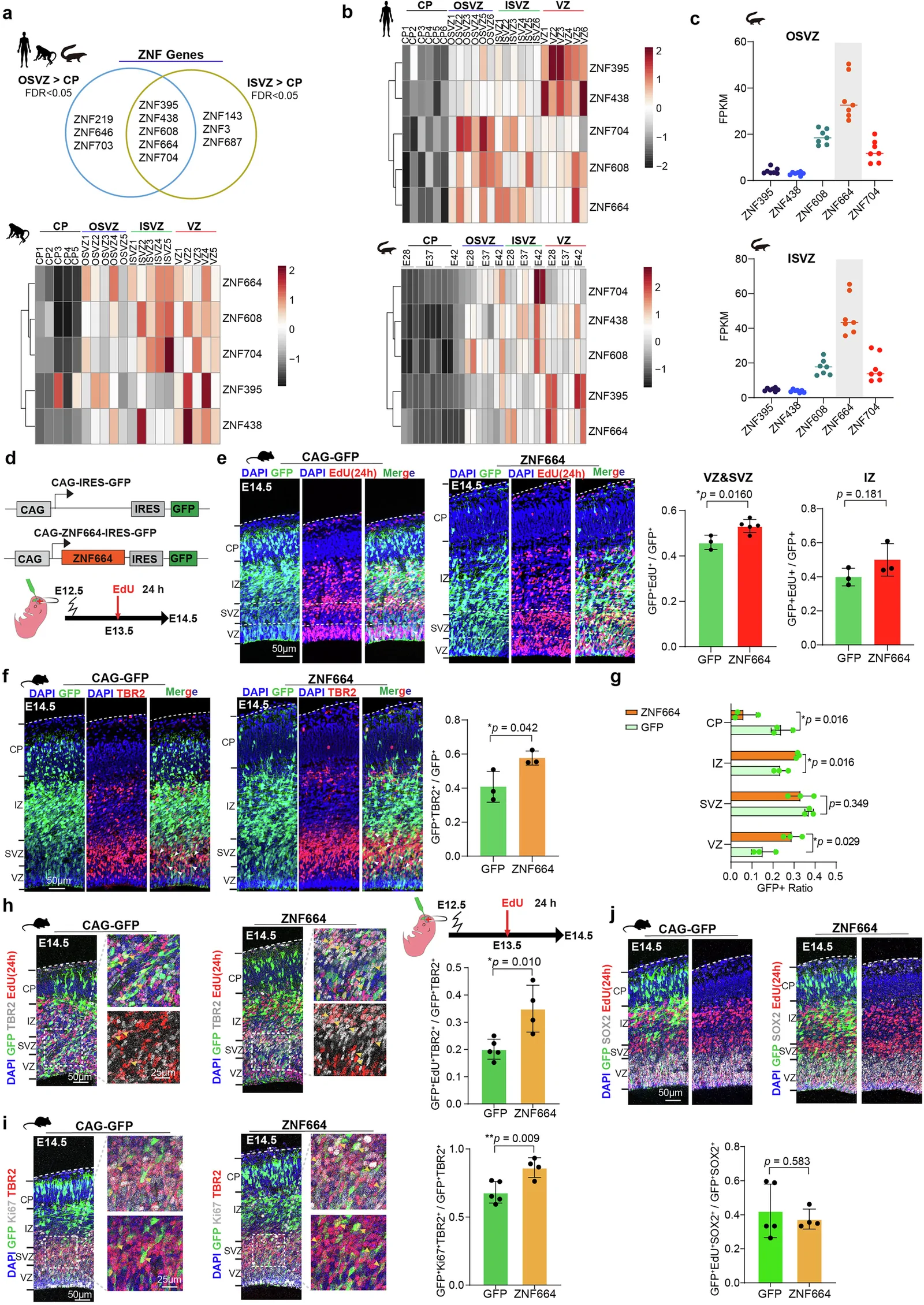
*ZNF664* expression promotes IPCs proliferation in mice. **a** Venn diagram plots showing ZNF genes shared between the OSVZ and ISVZ in human, macaque, and tree shrew. **b** Heatmap plots showing expression patterns of ZNF genes among tree shrew, monkey, and human species. **c** Scatter plots demonstrating the high expression of *ZNF664* among shared ZNF genes in both the OSVZ and ISVZ (*n* = 7 tree shrew samples). Note that *ZNF664* was highlighted in shadow. **d** Schematic diagram of the IUE (E12.5-E14.5) strategy, in which CAG-GFP and CAG-*ZNF664*-GFP constructs were electroporated at E12.5, followed by EdU labeling at 24 h and analysis at E14.5. **e** Quantification of GFP^+^ and *ZNF664*-GFP^+^ cells co-localized with EdU^+^ in the VZ, SVZ, or IZ (Embryos from two IUE experiments; VZ&SVZ: GFP, *n* = 3 embryos, ZNF664, *n* = 5 embryos; IZ: GFP, *n* = 3 embryos, ZNF664, *n* = 3 embryos; mea*n* ± SD; * represents *p* < 0.05; two-tailed unpaired *t*-test). White arrowheads indicate GFP^+^EdU^+^ double-positive cells. **f** Quantification of GFP^+^ and *ZNF664*-GFP^+^ colocalization with TBR2^+^ in the SVZ (*n* = 3, embryos; mea*n* ± SD; * represents *p* < 0.05; two-tailed unpaired *t-test*). White arrowheads indicate GFP^+^*TBR2*^+^ double-positive cells. **g** Quantification of GFP^+^ in different cortical layers (*n* = 3, embryos; mea*n* ± SD; *p < 0.05; two-tailed unpaired *t-test*). **h** Quantification of GFP^+^ and *ZNF664*-GFP^+^ colocalization with EdU^+^ TBR2^+^ in the SVZ (GFP: *n* = 5, embryos, ZNF664:*n* = 4 embryos; mea*n* ± SD; * represents *p* < 0.05, two-tailed unpaired *t-test*). Orange arrowheads indicate EdU+GFP^+^TBR2^+^ triple-positive cells. **i** Quantification of GFP^+^ and *ZNF664*-GFP^+^ colocalization with Ki67^+^ TBR2^+^ in the SVZ (GFP: *n* = 5, embryos, ZNF664:*n* = 4 embryos; mea*n* ± SD; ** represents *p* < 0.01, two-tailed unpaired *t-test*). Orange arrowheads indicate GFP^+^Ki67^+^TBR2^+^triple -positive cells. **j** Quantification of GFP^+^ and *ZNF664*-GFP^+^ colocalization with EdU^+^ SOX2^+^ in the VZ (GFP: *n* = 5, embryos, ZNF664:*n* = 4 embryos; mea*n* ± SD; two-tailed unpaired *t-test*).

Interestingly, three ZNF genes were specifically expressed in the OSVZ (*ZNF219*, *ZNF646*, *ZNF703*), whereas three others were specifically expressed in the ISVZ (*ZNF143*, *ZNF3*, *ZNF687*) (Fig. 7a). Five ZNF genes were expressed in both the ISVZ and OSVZ of human, macaque, and tree shrew cortices (*ZNF395*, *ZNF438*, *ZNF608*, *ZNF664*, *ZNF704*) (Fig. 7a, b). These genes exhibited little or no expression in mouse. Among these genes, *ZNF664* showed the most robust and consistent expression across the OSVZ and ISVZ in the tree shrew (Fig. 7c). *ZNF664* was also highly expressed in the VZ of human, macaque, and tree shrew cortices (Fig. 7b and Supplementary Fig. 9a). This conserved expression pattern suggested a potential role for *ZNF664* in neural progenitor proliferation.

To test this hypothesis, we performed in utero electroporation (IUE) of *ZNF664* in mouse embryos at E12.5, followed by EdU labeling at E13.5 for 24 h, and analyzed embryos at E15.5 (Fig. 7d). Remarkably, ZNF664 expression significantly increased progenitor proliferation in the mouse VZ/SVZ, but not in the IZ (Fig. 7e, right panel; EdU^+^GFP^+^/GFP^+^ from 45.96% to 54.51%, *unpaired t-test, p* = 0.023), while knock down of ZNF664 expression using small interfering RNA (siRNA) in human *SH-SY5Y* cells revealed a significant decrease of EdU^+^GFP^+^ cells (Supplementary Fig. 9b, c). Forced expression of *ZNF664* significantly increased the proportion of IPCs (*TBR2*^+^ cells) by 1.413-fold (Fig. 7f, *unpaired t-test, p* = 0.042). Quantitative analysis of GFP^+^ in different layers identified that the proportion of GFP^+^ cells was significantly increased in the VZ and IZ but not CP, suggesting a delay in neuron differentiation after ZNF664 expression (Fig. 7g). Another independent IUE experiments confirmed that ZNF664 expression could promote IPCs proliferation in the mouse (Fig. 7h, right panel; EdU^+^GFP^+^TBR2^+^/GFP^+^ TBR2^+^ from 20.12% to 34.98%, *unpaired t-test, p* = 0.010), and a higher percent of IPCs was observed in a proliferative status using Ki67 as a cell cycle marker (Fig. 7i, right panel; Ki67^+^GFP^+^TBR2^+^/GFP^+^ TBR2^+^ from 68.12% to 86.28%, *unpaired t-test, p* = 0.009). More importantly, ZNF664 expression didn’t increase the proportion of RGCs in mice (Fig. 7j), and future experiments are warranted to investigate whether ZNF664 can promote proliferation basal RGCs using primate brain organoids or tree shrew IUE experiments.

Together, these results demonstrate that *ZNF664* promotes the proliferation of intermediate progenitor cells.

## Discussion

In this study, we generated the first genome-wide expression map of fetal cortical layers in the tree shrew, a close outgroup to primates with an evolutionarily intermediate brain phenotype. DEG analysis demonstrated that, over the course of cortical development, synapse-related genes progressively increased their expression in the CP, IZ, VZ, and ISVZ, whereas neurogenesis-related genes were selectively enriched in the MZ and OSVZ. By integrating these data with the previously identified human OSVZ-specific genes and applying complementary single-cell analyses, we showed that most OSVZ-associated markers were already expressed in the tree shrew OSVZ, indicating that these molecular features originated prior to the divergence of tree shrews and primates. Comparative analyses further suggested that cell cycle-related genes were highly expressed in the tree shrew OSVZ before primate divergence, whereas ECM genes are specifically linked to OSVZ expansion in primates. In addition, we identified ZNF as candidate regulators of OSVZ development and evolution. Among these, *ZNF664* was detected in the tree shrew brain, and IUE experiments further demonstrated its functional role in promoting basal progenitor proliferation in the mouse SVZ.

Cell cycle regulation has been shown to play a significant role in enhancing the proliferative capacity of RGCs, thereby contributing to OSVZ expansion in primates^32,63,64^. Notably, our comparative analyses indicate that cell cycle regulation was already involved in OSVZ development in the tree shrew prior to primate divergence, whereas ECM genes were specifically enriched in the primate OSVZ. While evolutionary changes in cell cycle regulation can enhance RGC self-renewal and proliferative capacity, ECM components provide both structural support and signaling cues, thereby influencing diverse cellular behaviors, including proliferation^54,55^. For example, supplementation of human fetal neocortical cultures with ECM components such as proteoglycan link protein 1, lumican, and collagen I can induce local cortical folding^56^. Moreover, a recent study demonstrated that the ECM protein galectin-3 regulates human NPC division and delamination, and that its inhibition induces gyrification in the developing mouse cortex^65^. Therefore, the Chinese tree shrew, as an outgroup to primates, provides a valuable model for identifying novel ECM genes and investigating their potential roles in cortical complexification during primate evolution.

Genetic studies of OSVZ evolution have revealed the contributions from human-specific genes such as *ARHGAP11B* and *NOTCH2NL*^66–69^, great ape-specific genes including *CROCCP2*^70^ and *TBC1D3*^71^, as well as the primate-specific gene *TMEM14B*^72^. In this context, the identification of ZNF proteins that are highly expressed in the OSVZ is particularly noteworthy. Although previous studies have reported high expression of ZNF genes in the developing brain^73,74^, their functional roles in primate OSVZ evolution have remained largely unexplored. The functional characterization of *ZNF664*, which promotes BP proliferation, represents an initial step toward understanding the contribution of ZNF genes to OSVZ evolution. Elucidating the molecular mechanisms by which ZNF664 regulates basal progenitor behavior will require further investigation.

Some studies have used the ferret, a small carnivore with an expanded and folded cortex, as an animal model to investigate brain evolution and development^75^. Although these studies yielded important insights into cortical folding^76–78^, ferrets are more distantly related to primates than rodents. Given their close genetic distance to primates and intermediate brain phenotype, tree shrews represent a promising alternative animal model for studying brain evolution and development, particularly with respect to the OSVZ. Our study highlights the potential of this system for uncovering the genetic and molecular bases of primate- and human-specific evolutionary features, including the mechanisms and cortical circuits underlying human OSVZ evolution and development.

## Methods

### Ethics statement

All experimental procedures in this study strictly adhered to relevant ethical regulations and guidelines. All animal experiments were approved by the Animal Care and Use Committee of the Kunming Institute of Zoology, Chinese Academy of Sciences under approval number IACUC-TE-2023-05-003.

### Animal husbandry, randomization, and blinding

Chinese tree shrews and CD-1 mice were housed in the Laboratory Animal Center of the Kunming Institute of Zoology under specific-pathogen-free conditions at 22–25 °C, 50–60% relative humidity, and a 12-h light/dark cycle (lights on at 08:00), with ad libitum access to food and water. For embryo collection, randomization was not applicable because all pregnant females, as confirmed by palpation, were included. For in utero electroporation, pregnant mice were randomly assigned to experimental groups by a researcher independent of the surgical procedures and subsequent analyses. Blinding was not implemented for immunofluorescence and quantitative PCR analyses due to constraints of the experimental design; however, to mitigate potential bias, samples from different groups were intermixed and processed in random order, and all quantitative measurements were performed using identical parameter settings across all groups.

### Tree shrew embryonic and postnatal brain collections

To obtain embryos at defined developmental stages, male and female tree shrews were housed together overnight and separated the following day, with the day of separation designated as embryonic day 0 (E0). Approximately 10 days later, pregnancy was assessed by gentle abdominal palpation performed by trained veterinarians, allowing accurate determination of embryonic developmental stages.

Tree shrews with confirmed gestational ages were selected and anesthetized by intramuscular injection of pentobarbital sodium (50 mg/kg; Sigma, P3761). After the animals were deeply anesthetized (confirmed by loss of pedal withdrawal reflex). The abdomen was then opened layer by layer using sterile scissors and forceps, and embryos were carefully collected and placed in a Petri dish containing physiological saline. Following embryo removal, the dam was euthanized by inhalation of 5% isoflurane until respiratory arrest and cardiac arrest were confirmed. The embryonic skull was gently removed with forceps to isolate the brain, which was subsequently bisected along the midline into left and right hemispheres using a blade. One hemisphere was fixed in 4% paraformaldehyde (PFA) for subsequent immunofluorescence staining, whereas the other hemisphere was embedded in OCT (4583; Sakura, Torrance, CA, USA), gradually frozen in a dry ice-alcohol bath, and stored at −80 °C for laser microdissection and omics sequencing analyses. Newborn tree shrews and mice were directly anesthetized with isoflurane, and after removing the brains using the same method, the brains were fixed in 4% paraformaldehyde solution for 48–72 h for subsequent measurement of brain area. All animals were euthanized by cervical dislocation after deep anesthesia (confirmed by absence of heartbeat and respiration) prior to brain removal.

### Nissl staining and laser capture microdissection (LCM)

Preparation of the Nissl staining solution: 0.1 g of Cresyl Violet acetate (Sigma-Aldrich, C5042) was dissolved in 100 mL of RNase-free DEPC-treated water (Biosharp, BL510B). After filtration, the staining solution was kept on ice until use. To prevent RNA degradation, the entire staining procedure was performed on ice. Tissue slides were air-dried at room temperature until no visible moisture remained, then sequentially dehydrated in pre-cooled graded ethanol solutions (50% → 75% → 100%) for 1 min each. Sections were subsequently immersed in the pre-cooled Nissl staining solution for 2–3 min and immediately transferred to a laser microdissection system (LMD7000; Leica) for microdissection. Target tissue regions were cut and collected into 0.6 mL microtubes (MCT-060-C; Axygen, NY, USA) containing TRIzol (15596026; Invitrogen, Waltham, MA, USA). RNA was isolated using the miRNeasy Micro Kit (217084; QIAGEN, Hilden, Germany).

### RNA sequencing library construction

Transcriptome libraries were constructed using the VAHTS mRNA-seq V3 Library Prep Kit for Illumina (Vazyme, NR611). Briefly, mRNA was isolated and fragmented, then double-stranded cDNA was synthesized, purified, and end-repaired. Adapter ligation was then performed using Vazyme adapters (N803), after which the ligation products were purified and size-selected. Following library amplification and purification of PCR products, library quality was assessed using an Agilent 2100 Bioanalyzer. Specifically, 1 μL of the purified PCR product was analyzed with the Agilent DNA 1000 kit (Agilent, 5067-1504).

### Isolation of Germinal Zone (GZ) and single-cell sequencing

Freshly collected tree shrew embryonic brains were embedded in Optimal Cutting Temperature compound (O.C.T.; Sakura, 4583) using embedding cassettes and slowly frozen in a dry ice-alcohol mixture until complete solidification to preserve tissue integrity. Coronal sections (20–25 μm thick) containing the ventricles were then cut using a cryostat (Leica CM3050 S). Sections were immediately subjected to Nissl staining to visualize cortical cytoarchitecture. Following staining, the boundaries of the germinal zone (GZ) and cortical plate (CP) were precisely identified under a microscope to delineate the GZ region. The defined GZ area was carefully scored on the tissue block within the cryostat using a sterile fine blade, and the GZ tissue was subsequently dissected with a blade and forceps.

The isolated GZ tissue was immediately processed into a single-cell suspension to minimize degradation. The nuclear suspension was stained with 0.4% trypan blue (Invitrogen Thermo, T10282) and examined under a microscope to assess viability, clumping rate, background debris, and nuclear integrity. Samples that passed quality control were subsequently subjected to library preparation. The prepared single-cell suspension, oil, and beads were sequentially loaded onto C-series scRNA slides. Water-in-oil droplets were generated using the DNBelab C-TaiM 4 device, allowing cell lysis and mRNA capture by magnetic beads within individual droplets. Reverse transcription and demulsification were then performed by adding demulsification reagent to the cDNA, followed by room-temperature incubation, centrifugation, and product recovery. Magnetic beads were used to separate oligonucleotide and cDNA products. This was followed by cDNA amplification, oligonucleotide library construction, cDNA fragmentation, end repair and A-tailing, adapter ligation, PCR amplification, and library quality control. Sequencing was performed on the DNBSEQ-T7 platform with paired-end 100-bp reads, targeting approximately 50,000 reads per cell and ~600 million raw reads per sample.

### Real-time quantitative RT-PCR

For quantitative PCR (qPCR) analysis, total RNA was extracted from cultured cells using TRIzol reagent (Invitrogen, 15596018CN) according to the manufacturer’s instructions. RNA concentration and purity were assessed by spectrophotometry (NanoDrop ND2000), and samples with A260/A280 ratios between 1.8 and 2.0 were considered suitable for subsequent analyses. First-strand cDNA was synthesized from 1 μg of total RNA using the PrimeScript RT reagent kit (Takara, RR037A) with oligo(dT) primers in a 20 μL reaction volume. qPCR reactions were performed in triplicate using SYBR Green Master Mix (Vazyme, Q711) on an Applied Biosystems 7500 Real-Time PCR System (Waltham, MA, USA). Primer sequences were as follows: ZNF664 forward, 5’-CAAGTGCCCCATGTGTAGGG-3’ and reverse 5’-AAGGCTTTGCCACACTCGTA-3’; GAPDH forward 5’-ACAACTTTGGTATCGTGGAAGG-3’ and reverse 5’-GCCATCACGCCACAGTTTC-3’. Relative expression levels of ZNF664 were calculated using the 2^ΔΔCt method with GAPDH as the endogenous reference.

### *In Utero* electroporation

In utero electroporation (IUE) was performed as follows. Pregnant CD-1 mice were anesthetized using an animal anesthesia machine (R500; RWD Life Science, Shenzhen, China) with isoflurane (5% induction, 2–3% maintenance in 100% O2). After deep anesthesia (confirmed by lack of toe pinch response), mice were placed in the supine position with limbs secured using tape, and the abdominal surgical site was disinfected with 75% ethanol. The abdominal wall was then incised layer by layer using sterile scissors. Sterile surgical gauze was placed over the incision site, and the uterus was gently exteriorized and positioned on the gauze. From uterine exposure until completion of the procedure, warm physiological saline (40 °C) was continuously applied to the exposed uterus and intra-abdominal tissues to maintain moisture and temperature. After identifying the cerebral ventricles of the embryos, 1–2 μL of plasmid DNA containing 0.1% Fast Green (A610452; Sangon Biotech, Shanghai, China) was injected into the ventricles using a glass micropipette. Fast Green was used as a tracer to visualize the injection. A 3-mm positive electrode was placed on the injection side, and the negative electrode was positioned on the opposite side to minimize damage to other brain regions. Electric pulses were delivered using an electroporator (ECM830; BTX, MA, USA) with the following parameters: 28–30 V, 50 ms pulse duration, 1 s intervals, for a total of 5–6 pulses. After electroporation, the uterus was gently returned to the abdominal cavity, and the incision was sutured under sterile conditions, first closing the muscle layer followed by the skin. Mice were then placed in a warm environment and monitored until they fully recovered from anesthesia. No animals were excluded from the analysis. The number of embryos electroporated per litter ranged from 4 to 6, with at least three independent litters per experimental condition. Postoperative analgesia was provided by subcutaneous injection of buprenorphine (0.1 mg/kg) once immediately after surgery. At the experimental endpoint, all dams were deeply anesthetized with isoflurane (5% induction) and subsequently euthanized by cervical dislocation, with death confirmed by the cessation of heartbeat and respiration.

### Immunofluorescence staining and imaging

Post-dissection brains were fixed in 4% paraformaldehyde (PFA) for 48 h. After fixation, the tissues were embedded in 4% agarose and sectioned into 30–50 μm slices using a vibrating microtome (VT1000S; Leica). Brain sections from comparable anatomical locations were selected for immunofluorescence staining.

The staining procedure was performed as follows. After antigen retrieval with citrate buffer (G1202; Servicebio, Wuhan, China), sections were washed three times with PBS (5 min each) and incubated for 1–2 h at room temperature in blocking buffer containing PBS with 5% normal donkey serum (017-000-121; Jackson ImmunoResearch, PA, USA), 1% bovine serum albumin (BSA; SRE0096, Sigma), 0.1% glycine, 0.1% lysine, and 0.3% Triton X-100. Sections were then incubated with primary antibodies diluted in blocking buffer overnight at 4 °C. After three additional washes in PBS (5 min each), sections were incubated with secondary antibodies for 1–2 h at room temperature in the dark, followed by nuclear counterstaining with DAPI (1:1000; 62248, Invitrogen, CA, USA). Sections were coverslipped using antifade mounting medium (H-1000; Vector Laboratories, CA, USA). Images were acquired using a confocal microscope (TCS SP8; Leica) and processed with ImageJ software (National Institutes of Health).

Detailed antibody information (including catalog numbers, clone names, and lot numbers where applicable) is provided below: rabbit polyclonal anti-SATB2 (1:300; Affinity, DF2962; polyclonal; lot #AF402611), rabbit polyclonal anti-TBR1 (1:300; Affinity, DF2396; polyclonal; lot #AF31061), Mouse anti-BRN2 (1:500, Santa Cruz Biotechnology,sc-393324; clone B-2; lot #E2322), rat monoclonal anti-SOX2 (1:500; Invitrogen, 53-9811-82; clone Btjce; lot #2275462), Mouse Monoclonal anti-SOX2 (L1D6A2)(1:500;Cell Signaling Technology, 4900 T; clone L1D6A2), rabbit polyclonal anti-TBR2/EOMES (1:500; Abcam, ab23345; polyclonal; lot #GR3379077-1), rabbit polyclonal anti-PTPRZ1 (1:500; Sigma-Aldrich, HPA015103; polyclonal; lot #R02658). Secondary antibodies included Alexa Fluor 488 AffiniPure donkey anti-rat (1:800; Jackson ImmunoResearch, 712-545-150; lot #156282), Alexa Fluor 594 AffiniPure donkey anti-rat (1:800; Jackson ImmunoResearch, 712-585-153; lot #157934), Alexa Fluor 488 AffiniPure donkey anti-rabbit (1:800; Jackson ImmunoResearch, 711-545-152; lot #153614), and Alexa Fluor 594 AffiniPure donkey anti-rabbit (1:800; Jackson ImmunoResearch, 711-585-152; lot #160725).

### Cell culture and transfection

Neuro-2a (N2A) cells were maintained in complete growth medium consisting of

DMEM (Gibco, C11995500BT) supplemented with 10% fetal bovine serum (FBS; Gibco, A5669401) and 1% penicillin/streptomycin (Gibco, 15140122) at 37 °C in a humidified incubator with 5% CO_2_. For routine passaging, cells at approximately 80% confluency were washed with PBS, detached using 0.25% trypsin-EDTA (Gibco, 25200056) for 2–3 min at 37 °C, and neutralized with complete medium prior to centrifugation (300 × *g*, 5 min). Cells were resuspended and subcultured in 100-mm culture dishes (Nest, 704202). For transfection experiments, cells were seeded in 6-well plates at a density of 2 × 10^5^ cells per well in antibiotic-free medium 1 day before transfection. Lipofectamine 3000-mediated transfection (Invitrogen, L3000008) was performed using two solutions: solution A contained 2.5 μg plasmid DNA mixed with 5 μL P3000 enhancer in 125 μL Opti-MEM (Gibco, 11058021), and solution B contained 3.75 μL Lipofectamine 3000 in 125 μL Opti-MEM. After incubation for 5 min at room temperature, the two solutions were combined (total volume, 250 μL) and incubated for an additional 15–20 min to allow complex formation before being added dropwise to the cells. The transfection medium was replaced with complete growth medium after 6–8 h, and transfection efficiency was assessed 24–48 h post-transfection by fluorescence microscopy and quantitative PCR (qPCR).

### Knockdown of ZNF664 by siRNA

Double-stranded small interfering RNA (siRNA) targeting the coding region of endogenous *ZNF664* was designed and synthesized by Tsingke Biotechnology (Beijing, China). The siRNA sequences used were as follows: siRNA1-sense strand: CAGGAGAGAAGGUCUAUAA(dT)(dT), siRNA1-antisense strand: UUAUAGACCUUCUCUCCUG(dT)(dT); siRNA2-sense strand: CAGUGGAGAAGCCCUAUAA(dT)(dT), siRNA2-antisense strand: UUAUAGGGCUUCUCCACUG(dT)(dT). Negative and positive control siRNAs were obtained from the manufacturer.

Transfection was performed according to the manufacturer’s instructions, and the final siRNA concentration was 50 nM. After transfection, the medium was replaced with complete culture medium, and the cells were cultured for 48 h. Knockdown efficiency was then assessed by quantitative PCR.

### Targeting expression vector cloning

The full-length coding sequence of human *ZNF664* was synthesized by a commercial gene synthesis service (Beijing Tsingke Biotech Co., Ltd.), PCR-amplified, and independently cloned into the GFP-tagged expression vector pCS2-eGFP-N and IUE expression plasmid CAG-IRES-GFP.

### Differentially expressed gene analysis

Differentially expressed genes (DEGs) were identified from integer count data using the DESeq2 R package^79^, which models count data with a negative binomial distribution. Size factors were estimated to normalize for differences in sequencing depth across samples. Gene-specific dispersion parameters were then estimated to account for biological variability between samples, and counts for each gene were fitted to a negative binomial model. Statistical significance was assessed using the Wald test, and *P* values were adjusted for multiple testing using the Benjamini-Hochberg procedure to control the false discovery rate.

### Chinese tree shrew RNA-seq read mapping

Illumina sequencing reads were processed by trimming adapter sequences and mapped to the Chinese tree shrew genome (TS_3.0 annotation)^36^ using HISAT v2.1.0. Gene expression levels were quantified from the mapped reads using HTSeq-count^80^, which generated integer read counts for each gene. Cufflinks v2.2.1^81^ was used to calculate fragments per kilobase of transcript per million mapped reads (FPKM) values. Differentially expressed genes were subsequently identified from integer count data using the DESeq2 R package^79^.

### Human, macaque, tree shrew and mouse lamina RNA-seq data

Raw count data and FPKM values for human, macaque, tree shrew E37 VZ, ISVZ, OSVZ, CP, and mouse neocortical laminae were obtained from the Gene Expression Omnibus (GEO) database (GSE38805) and from our previous studies^42,57,58^. DEGs were identified from integer count data using DESeq2.

### Gene ontology (GO) analysis and protein-protein interaction analysis

DEGs for GO biological processes and molecular functions were assessed using the ToppGene Suite^82^. *P* values were adjusted for multiple testing using the Benjamini-Hochberg correction, with an adjusted *P* < 0.05 considered statistically significant. Protein-protein interaction networks were constructed using the STRING database^83^ following the standard workflow.

### Cluster analysis of gene expression data

The *regularized log (rlog)* transformation converts raw count data to the log2 scale by fitting a model that includes a term for each sample and a prior distribution on coefficients estimated from the data. Hierarchical clustering analyses were then performed using the pheatmap R package based on the rlog-transformed values.

### Single-cell data analysis

Single-cell data analysis was performed using the Seurat R package^84^. Feature barcode matrices were processed and analyzed using the RunUMAP function in Seurat for nonlinear dimensionality reduction. Integrated analyses were subsequently performed on all cells following the standard Seurat workflow.

### Reporting summary

Further information on research design is available in the Nature Portfolio Reporting Summary linked to this article.

## Supplementary information

Supplementary information

Dataset 1

Dataset 2

Dataset 3

Dataset 4

Dataset 5

Dataset 6

Dataset 7

Dataset 8

Dataset 9

Dataset 10

Reporting Summary

Peer Review File

## Data Availability

The RNA-seq data generated in this study have been deposited in the NCBI Gene Expression Omnibus (GEO) database under the accession number GSE318518, and also in the Genome Sequence Archive (GSA) under accession numbers CRA038045, CRA044752, and are publicly accessible at https://ngdc.cncb.ac.cn/gsa. The source data for Fig. 1, Fig. 2, Fig. 5, Fig. 7, and Supplementary Fig. 2, Supplementary Fig. 9 are in Supplementary Data 10.

## Code availability

We didn’t generate specific codes for this study. All codes are based on the customer’s software and are available upon request.

## References

[1] Sousa, A. M. M., Meyer, K. A., Santpere, G., Gulden, F. O. & Sestan, N. Evolution of the human nervous system function, structure, and development. *Cell* **170**, 226–247 (2017). 28708995 10.1016/j.cell.2017.06.036 PMC5647789

[2] Pollen, A. A., Kilik, U., Lowe, C. B. & Camp, J. G. Human-specific genetics: new tools to explore the molecular and cellular basis of human evolution. *Nat. Rev. Genet* **24**, 687–711 (2023). 36737647 10.1038/s41576-022-00568-4 PMC9897628

[3] Geschwind, D. H. & Rakic, P. Cortical evolution: judge the brain by its cover. *Neuron* **80**, 633–647 (2013). 24183016 10.1016/j.neuron.2013.10.045 PMC3922239

[4] Konopka, G. & Geschwind, D. H. Human brain evolution: harnessing the genomics (r)evolution to link genes, cognition, and behavior. *Neuron* **68**, 231–244 (2010). 20955931 10.1016/j.neuron.2010.10.012 PMC2993319

[5] Pollard, K. S. et al. An RNA gene expressed during cortical development evolved rapidly in humans. *Nature* **443**, 167–172 (2006). 16915236 10.1038/nature05113

[6] Zhang, J. Evolution of the human ASPM gene, a major determinant of brain size. *Genetics* **165**, 2063–2070 (2003). 14704186 10.1093/genetics/165.4.2063 PMC1462882

[7] Shi, L., Li, M., Lin, Q., Qi, X. & Su, B. Functional divergence of the brain-size regulating gene MCPH1 during primate evolution and the origin of humans. *BMC Biol.* **11**, 62 (2013). 23697381 10.1186/1741-7007-11-62 PMC3674976

[8] Somel, M. et al. MicroRNA-driven developmental remodeling in the brain distinguishes humans from other primates. *PLoS Biol.* **9**, e1001214 (2011). 22162950 10.1371/journal.pbio.1001214 PMC3232219

[9] Briggs, J. A., Wolvetang, E. J., Mattick, J. S., Rinn, J. L. & Barry, G. Mechanisms of long non-coding RNAs in mammalian nervous system development, plasticity, disease, and evolution. *Neuron* **88**, 861–877 (2015). 26637795 10.1016/j.neuron.2015.09.045

[10] Soto, D. C. et al. Human-specific gene expansions contribute to brain evolution. *Cell* **188**, 5363–5383 (2025). 10.1016/j.cell.2025.06.037 PMC12313184 40695280

[11] Karageorgiou, C., Gokcumen, O. & Dennis, M. Y. Deciphering the role of structural variation in human evolution: a functional perspective. *Curr. Opin. Genet Dev.* **88**, 102240 (2024). 39121701 10.1016/j.gde.2024.102240 PMC11485010

[12] Jeong, H. et al. Evolution of DNA methylation in the human brain. *Nat. Commun.* **12**, 2021 (2021). 33795684 10.1038/s41467-021-21917-7 PMC8017017

[13] Reilly, S. K. et al. Evolutionary genomics. Evolutionary changes in promoter and enhancer activity during human corticogenesis. *Science* **347**, 1155–1159 (2015). 25745175 10.1126/science.1260943 PMC4426903

[14] Jorstad, N. L. et al. Comparative transcriptomics reveals human-specific cortical features. *Science* **382**, eade9516 (2023). 37824638 10.1126/science.ade9516 PMC10659116

[15] Ma, S. et al. Molecular and cellular evolution of the primate dorsolateral prefrontal cortex. *Science* **377**, eabo7257 (2022). 36007006 10.1126/science.abo7257 PMC9614553

[16] He, Z. et al. Comprehensive transcriptome analysis of neocortical layers in humans, chimpanzees and macaques. *Nat. Neurosci.* **20**, 886–895 (2017). 28414332 10.1038/nn.4548

[17] Goodman, M. & Sterner, K. N. Colloquium paper: phylogenomic evidence of adaptive evolution in the ancestry of humans. *Proc. Natl. Acad. Sci. USA* **107**, 8918–8923 (2010). 20445097 10.1073/pnas.0914626107 PMC3024020

[18] Vallender, E. J., Mekel-Bobrov, N. & Lahn, B. T. Genetic basis of human brain evolution. *Trends Neurosci.* **31**, 637–644 (2008). 18848363 10.1016/j.tins.2008.08.010 PMC2715140

[19] Lancaster, M. A. Unraveling mechanisms of human brain evolution. *Cell* **187**, 5838–5857 (2024). 39423803 10.1016/j.cell.2024.08.052 PMC7617105

[20] Namba, T. & Huttner, W. B. What Makes Us Human: Insights from the Evolution and Development of the Human Neocortex. *Annu Rev. Cell Dev. Biol.* **40**, 427–452 (2024). 39356810 10.1146/annurev-cellbio-112122-032521

[21] Lindhout, F. W., Krienen, F. M., Pollard, K. S. & Lancaster, M. A. A molecular and cellular perspective on human brain evolution and tempo. *Nature* **630**, 596–608 (2024). 38898293 10.1038/s41586-024-07521-x

[22] Lewitus, E., Kelava, I., Kalinka, A. T., Tomancak, P. & Huttner, W. B. An adaptive threshold in mammalian neocortical evolution. *PLoS Biol.* **12**, e1002000 (2014). 25405475 10.1371/journal.pbio.1002000 PMC4236020

[23] Libe-Philippot, B. & Vanderhaeghen, P. Cellular and molecular mechanisms linking human cortical development and evolution. *Annu Rev. Genet* **55**, 555–581 (2021). 34535062 10.1146/annurev-genet-071719-020705

[24] Kwan, K. Y., Sestan, N. & Anton, E. S. Transcriptional co-regulation of neuronal migration and laminar identity in the neocortex. *Development* **139**, 1535–1546 (2012). 22492350 10.1242/dev.069963 PMC3317962

[25] Lui, J. H., Hansen, D. V. & Kriegstein, A. R. Development and evolution of the human neocortex. *Cell* **146**, 18–36 (2011). 21729779 10.1016/j.cell.2011.06.030 PMC3610574

[26] Llinares-Benadero, C. & Borrell, V. Deconstructing cortical folding: genetic, cellular and mechanical determinants. *Nat. Rev. Neurosci.* **20**, 161–176 (2019). 30610227 10.1038/s41583-018-0112-2

[27] Kennedy, H., Wianny, F. & Dehay, C. Determinants of primate neurogenesis and the deployment of top-down generative networks in the cortical hierarchy. *Curr. Opin. Neurobiol.* **66**, 69–76 (2021). 33099180 10.1016/j.conb.2020.09.012

[28] Dehay, C. & Huttner, W. B. Development and evolution of the primate neocortex from a progenitor cell perspective. *Development* **151**, dev199797 (2024). 10.1242/dev.199797 38369736

[29] Fietz, S. A. et al. OSVZ progenitors of human and ferret neocortex are epithelial-like and expand by integrin signaling. *Nat. Neurosci.* **13**, 690–699 (2010). 20436478 10.1038/nn.2553

[30] Kalebic, N. & Huttner, W. B. Basal progenitor morphology and neocortex evolution. *Trends Neurosci.* **43**, 843–853 (2020). 32828546 10.1016/j.tins.2020.07.009

[31] Akula, S. K., Exposito-Alonso, D. & Walsh, C. A. Shaping the brain: the emergence of cortical structure and folding. *Dev. Cell* **58**, 2836–2849 (2023). 38113850 10.1016/j.devcel.2023.11.004 PMC10793202

[32] Dehay, C., Kennedy, H. & Kosik, K. S. The outer subventricular zone and primate-specific cortical complexification. *Neuron* **85**, 683–694 (2015). 25695268 10.1016/j.neuron.2014.12.060

[33] Wang, X., Tsai, J. W., LaMonica, B. & Kriegstein, A. R. A new subtype of progenitor cell in the mouse embryonic neocortex. *Nat. Neurosci.* **14**, 555–561 (2011). 21478886 10.1038/nn.2807 PMC3083489

[34] Hansen, D. V., Lui, J. H., Parker, P. R. & Kriegstein, A. R. Neurogenic radial glia in the outer subventricular zone of human neocortex. *Nature* **464**, 554–561 (2010). 20154730 10.1038/nature08845

[35] Fan, Y. et al. Genome of the Chinese tree shrew. *Nat. Commun.* **4**, 1426 (2013). 23385571 10.1038/ncomms2416

[36] Ye, M. S. et al. Comprehensive annotation of the Chinese tree shrew genome by large-scale RNA sequencing and long-read isoform sequencing. *Zool. Res* **42**, 692–709 (2021). 34581030 10.24272/j.issn.2095-8137.2021.272 PMC8645884

[37] Shao, Y. et al. Phylogenomic analyses provide insights into primate evolution. *Science* **380**, 913–924 (2023). 37262173 10.1126/science.abn6919

[38] Son, D. R. et al. Whole-genome DNA methylomes of tree shrew brains reveal conserved and divergent roles of DNA methylation on sex chromosome regulation. *BMC Biol.* **22**, 277 (2024). 39609804 10.1186/s12915-024-02071-0 PMC11603898

[39] Yin, C. et al. Abundant self-amplifying intermediate progenitors in the subventricular zone of the Chinese tree shrew neocortex. *Cereb. Cortex* **30**, 3370–3380 (2020). 32080709 10.1093/cercor/bhz315

[40] Romer, S. et al. Neural progenitors in the developing neocortex of the northern tree shrew (Tupaia belangeri) show a closer relationship to gyrencephalic primates than to lissencephalic rodents. *Front Neuroanat.* **12**, 29 (2018). 29725291 10.3389/fnana.2018.00029 PMC5917011

[41] Reillo, I., de Juan Romero, C., Garcia-Cabezas, M. A. & Borrell, V. A role for intermediate radial glia in the tangential expansion of the mammalian cerebral cortex. *Cereb. Cortex* **21**, 1674–1694 (2011). 21127018 10.1093/cercor/bhq238

[42] Hu, T. et al. Cis-regulatory evolution of CCNB1IP1 driving gradual increase of cortical size and folding in primates. *bioRxiv, 2024.2012.2008.627376* (2024).

[43] Cadwell, C. R., Bhaduri, A., Mostajo-Radji, M. A., Keefe, M. G. & Nowakowski, T. J. Development and arealization of the cerebral cortex. *Neuron* **103**, 980–1004 (2019). 31557462 10.1016/j.neuron.2019.07.009 PMC9245854

[44] Workman, A. D., Charvet, C. J., Clancy, B., Darlington, R. B. & Finlay, B. L. Modeling transformations of neurodevelopmental sequences across mammalian species. *J. Neurosci.* **33**, 7368–7383 (2013). 23616543 10.1523/JNEUROSCI.5746-12.2013 PMC3928428

[45] Rakic, P. Evolution of the neocortex: a perspective from developmental biology. *Nat. Rev. Neurosci.* **10**, 724–735 (2009). 19763105 10.1038/nrn2719 PMC2913577

[46] Kostic, M. et al. YAP activity is necessary and sufficient for basal progenitor abundance and proliferation in the developing neocortex. *Cell Rep.* **27**, 1103–1118.e1106 (2019). 31018127 10.1016/j.celrep.2019.03.091 PMC6486488

[47] Gonzalez-Bohorquez, D. et al. FASN-dependent de novo lipogenesis is required for brain development. *Proc. Natl. Acad. Sci. USA* **119**, e2112040119 (2022). 10.1073/pnas.2112040119 PMC8764667 34996870

[48] Shi, L., Qalieh, A., Lam, M. M., Keil, J. M. & Kwan, K. Y. Robust elimination of genome-damaged cells safeguards against brain somatic aneuploidy following Knl1 deletion. *Nat. Commun.* **10**, 2588 (2019). 31197172 10.1038/s41467-019-10411-w PMC6565622

[49] Spratt, P. W. E. et al. The autism-associated gene Scn2a contributes to dendritic excitability and synaptic function in the prefrontal cortex. *Neuron* **103**, 673–685.e675 (2019). 31230762 10.1016/j.neuron.2019.05.037 PMC7935470

[50] Kwan, K. Y. et al. Species-dependent posttranscriptional regulation of NOS1 by FMRP in the developing cerebral cortex. *Cell* **149**, 899–911 (2012). 22579290 10.1016/j.cell.2012.02.060 PMC3351852

[51] Louvi, A. & Artavanis-Tsakonas, S. Notch signalling in vertebrate neural development. *Nat. Rev. Neurosci.* **7**, 93–102 (2006). 16429119 10.1038/nrn1847

[52] Pollen, A. A. et al. Molecular identity of human outer radial glia during cortical development. *Cell* **163**, 55–67 (2015). 26406371 10.1016/j.cell.2015.09.004 PMC4583716

[53] Andrews, M. G., Subramanian, L. & Kriegstein, A. R. mTOR signaling regulates the morphology and migration of outer radial glia in developing human cortex. *Elife* **9**, e58737 (2020). 10.7554/eLife.58737 PMC7467727 32876565

[54] Amin, S. & Borrell, V. The extracellular matrix in the evolution of cortical development and folding. *Front Cell Dev. Biol.* **8**, 604448 (2020). 33344456 10.3389/fcell.2020.604448 PMC7744631

[55] Long, K. R. & Huttner, W. B. The role of the extracellular matrix in neural progenitor cell proliferation and cortical folding during human neocortex development. *Front Cell Neurosci.* **15**, 804649 (2021). 35140590 10.3389/fncel.2021.804649 PMC8818730

[56] Long, K. R. et al. Extracellular matrix components HAPLN1, lumican, and collagen i cause hyaluronic acid-dependent folding of the developing human neocortex. *Neuron* **99**, 702–719.e706 (2018). 30078576 10.1016/j.neuron.2018.07.013

[57] Fietz, S. A. et al. Transcriptomes of germinal zones of human and mouse fetal neocortex suggest a role of extracellular matrix in progenitor self-renewal. *Proc. Natl. Acad. Sci. USA* **109**, 11836–11841 (2012). 22753484 10.1073/pnas.1209647109 PMC3406833

[58] Shi, L. et al. Transgenic rhesus monkeys carrying the human MCPH1 gene copies show human-like neoteny of brain development. *Natl. Sci. Rev.* **6**, 480–493 (2019). 34691896 10.1093/nsr/nwz043 PMC8291473

[59] Bedogni, F. & Hevner, R. F. Cell-type-specific gene expression in developing mouse neocortex: intermediate progenitors implicated in axon development. *Front Mol. Neurosci.* **14**, 686034 (2021). 34321999 10.3389/fnmol.2021.686034 PMC8313239

[60] Di Bella, D. J. et al. Molecular logic of cellular diversification in the mouse cerebral cortex. *Nature* **595**, 554–559 (2021). 34163074 10.1038/s41586-021-03670-5 PMC9006333

[61] Turelli, P. et al. Primate-restricted KRAB zinc finger proteins and target retrotransposons control gene expression in human neurons. *Sci. Adv.* **6**, eaba3200 (2020). 32923624 10.1126/sciadv.aba3200 PMC7455193

[62] Johansson, P. A. et al. A cis-acting structural variation at the locus controls a gene regulatory network in human brain development. *Cell Stem Cell* **29**, 52–69 (2022). 34624206 10.1016/j.stem.2021.09.008

[63] Betizeau, M. et al. Precursor diversity and complexity of lineage relationships in the outer subventricular zone of the primate. *Neuron* **80**, 442–457 (2013). 24139044 10.1016/j.neuron.2013.09.032

[64] Lukaszewicz, A. et al. G1 phase regulation, area-specific cell cycle control, and cytoarchitectonics in the primate cortex. *Neuron* **47**, 353–364 (2005). 16055060 10.1016/j.neuron.2005.06.032 PMC1890568

[65] Soares, L. C. et al. Galectin-3 induces neurodevelopmental apical-basal polarity and regulates gyrification. *Sci. Adv.* **11**, eadt5859 (2025). 40901969 10.1126/sciadv.adt5859 PMC12407091

[66] Fiddes, I. T. et al. Human-specific NOTCH2NL genes affect notch signaling and cortical neurogenesis. *Cell* **173**, 1356–1369.e1322 (2018). 29856954 10.1016/j.cell.2018.03.051 PMC5986104

[67] Florio, M. et al. Human-specific gene ARHGAP11B promotes basal progenitor amplification and neocortex expansion. *Science* **347**, 1465–1470 (2015). 25721503 10.1126/science.aaa1975

[68] Heide, M. et al. Human-specific ARHGAP11B increases size and folding of primate neocortex in the fetal marmoset. *Science* **369**, 546–550 (2020). 32554627 10.1126/science.abb2401

[69] Suzuki, I. K. et al. Human-specific NOTCH2NL genes expand cortical neurogenesis through delta/notch regulation. *Cell* **173**, 1370–1384.e1316 (2018). 29856955 10.1016/j.cell.2018.03.067 PMC6092419

[70] Van Heurck, R. et al. CROCCP2 acts as a human-specific modifier of cilia dynamics and mTOR signaling to promote expansion of cortical progenitors. *Neuron* **111**, 65–80.e66 (2023). 36334595 10.1016/j.neuron.2022.10.018 PMC9831670

[71] Ju, X. C. et al. The hominoid-specific gene TBC1D3 promotes generation of basal neural progenitors and induces cortical folding in mice. *Elife* **5**, e18197 (2016). 10.7554/eLife.18197 PMC5028191 27504805

[72] Liu, J. et al. The primate-specific gene TMEM14B marks outer radial glia cells and promotes cortical expansion and folding. *Cell Stem Cell* **21**, 635–649.e638 (2017). 29033352 10.1016/j.stem.2017.08.013

[73] Farmiloe, G., Lodewijk, G. A., Robben, S. F., van Bree, E. J. & Jacobs, F. M. J. Widespread correlation of KRAB zinc finger protein binding with brain-developmental gene expression patterns. *Philos. Trans. R. Soc. Lond. B Biol. Sci.* **375**, 20190333 (2020). 32075554 10.1098/rstb.2019.0333 PMC7061980

[74] Playfoot, C. J. et al. Transposable elements and their KZFP controllers are drivers of transcriptional innovation in the developing human brain. *Genome Res* **31**, 1531–1545 (2021). 34400477 10.1101/gr.275133.120 PMC8415367

[75] Gilardi, C. & Kalebic, N. The ferret as a model system for neocortex development and evolution. *Front Cell Dev. Biol.* **9**, 661759 (2021). 33996819 10.3389/fcell.2021.661759 PMC8118648

[76] Singh, A. et al. Gene regulatory landscape of cerebral cortex folding. *Sci. Adv.* **10**, eadn1640 (2024). 38838158 10.1126/sciadv.adn1640 PMC11152136

[77] Shinmyo, Y. et al. Localized astrogenesis regulates gyrification of the cerebral cortex. *Sci. Adv.* **8**, eabi5209 (2022). 35275722 10.1126/sciadv.abi5209 PMC8916738

[78] Matsumoto, N., Shinmyo, Y., Ichikawa, Y. & Kawasaki, H. Gyrification of the cerebral cortex requires FGF signaling in the mammalian brain. *Elife* **6**, e29285 (2017). 10.7554/eLife.29285 PMC5685484 29132503

[79] Anders, S. & Huber, W. Differential expression analysis for sequence count data. *Genome Biol.* **11**, R106 (2010). 20979621 10.1186/gb-2010-11-10-r106 PMC3218662

[80] Anders, S., Pyl, P. T. & Huber, W. HTSeq-a Python framework to work with high-throughput sequencing data. *Bioinformatics* **31**, 166–169 (2015). 25260700 10.1093/bioinformatics/btu638 PMC4287950

[81] Trapnell, C. et al. Differential analysis of gene regulation at transcript resolution with RNA-seq. *Nat. Biotechnol.* **31**, 46–53 (2013). 23222703 10.1038/nbt.2450 PMC3869392

[82] Huang da, W., Sherman, B. T. & Lempicki, R. A. Bioinformatics enrichment tools: paths toward the comprehensive functional analysis of large gene lists. *Nucleic Acids Res.* **37**, 1–13 (2009). 19033363 10.1093/nar/gkn923 PMC2615629

[83] Szklarczyk, D. et al. The STRING database in 2023: protein-protein association networks and functional enrichment analyses for any sequenced genome of interest. *Nucleic Acids Res.* **51**, D638–D646 (2023). 36370105 10.1093/nar/gkac1000 PMC9825434

[84] Butler, A., Hoffman, P., Smibert, P., Papalexi, E. & Satija, R. Integrating single-cell transcriptomic data across different conditions, technologies, and species. *Nat. Biotechnol.* **36**, 411–420 (2018). 29608179 10.1038/nbt.4096 PMC6700744

