# Supplementary material for: Transcriptome of fetal cortex of tree shrew underlying the emergence of outer subventricular zone
Source: Commun Biol. 2026 Sep 14;9:1179. doi: 10.1038/s42003-026-10802-y (PMC13575156; doi:10.1038/s42003-026-10802-y)
Supplement: Supplementary file 1 [file 42003_2026_10802_MOESM12_ESM.pdf]

nature portfolio

Corresponding author(s): Lei Shi

Last updated by author(s): Jul 25, 2026

Reporting Summary

Nature Portfolio wishes to improve the reproducibility of the work that we publish. This form provides structure for consistency and transparency in reporting. For further information on Nature Portfolio policies, see our Editorial Policies and the Editorial Policy Checklist.

Statistics

For all statistical analyses, confirm that the following items are present in the figure legend, table legend, main text, or Methods section.

n/a	Confirmed	
☐	☒	The exact sample size (*n*) for each experimental group/condition, given as a discrete number and unit of measurement
☐	☒	A statement on whether measurements were taken from distinct samples or whether the same sample was measured repeatedly
☐	☒	The statistical test(s) used AND whether they are one- or two-sided<br>*Only common tests should be described solely by name; describe more complex techniques in the Methods section.*
☒	☐	A description of all covariates tested
☒	☐	A description of any assumptions or corrections, such as tests of normality and adjustment for multiple comparisons
☒	☐	A full description of the statistical parameters including central tendency (e.g. means) or other basic estimates (e.g. regression coefficient) AND variation (e.g. standard deviation) or associated estimates of uncertainty (e.g. confidence intervals)
☐	☒	For null hypothesis testing, the test statistic (e.g. *F*, *t*, *r*) with confidence intervals, effect sizes, degrees of freedom and *P* value noted<br>*Give P values as exact values whenever suitable.*
☒	☐	For Bayesian analysis, information on the choice of priors and Markov chain Monte Carlo settings
☒	☐	For hierarchical and complex designs, identification of the appropriate level for tests and full reporting of outcomes
☒	☐	Estimates of effect sizes (e.g. Cohen's *d*, Pearson's *r*), indicating how they were calculated

Our web collection on statistics for biologists contains articles on many of the points above.

Software and code

Policy information about availability of computer code

Data collection	Images were taken with confocal (Leica TCS SP8); The tree shrew target cortical layers were dissected using Leica laser micro-dissection system (Leica, LMD7000). SH-SY5Y Cell images were collected using Olympus Optiplex 7080.
Data analysis	Bulk RNA sequencing reads were analyzed using Hista2, Samtools, HTSeq-count, cufflinks et al softwares. Single cell datasets were analyzed by Seurat_5.1.0. Images were analyzed using Image J (Fiji). All statistical analyses were conducted using GraphPad Prism (8.4.0) and R version (4.2.1).

For manuscripts utilizing custom algorithms or software that are central to the research but not yet described in published literature, software must be made available to editors and reviewers. We strongly encourage code deposition in a community repository (e.g. GitHub). See the Nature Portfolio guidelines for submitting code & software for further information.

Data

Policy information about availability of data

All manuscripts must include a data availability statement. This statement should provide the following information, where applicable:

- Accession codes, unique identifiers, or web links for publicly available datasets
- A description of any restrictions on data availability
- For clinical datasets or third party data, please ensure that the statement adheres to our policy

All sequencing data were deposited at the NCBI Gene Expression Omnibus (GEO) under accession number GSE247966 (NCBI GEO), and also Genome Sequence Archine (GSA) under accession number CRA038045 and are publicly accessible at https://ngdc.cncb.ac.cn/gsa.

Research involving human participants, their data, or biological material

Policy information about studies with human participants or human data. See also policy information about sex, gender (identity/presentation), and sexual orientation and race, ethnicity and racism.

Reporting on sex and gender	n/a
Reporting on race, ethnicity, or other socially relevant groupings	n/a
Population characteristics	n/a
Recruitment	n/a
Ethics oversight	Tree shrew tissue samples, mouse embryos, and postnatal samples were obtained from the Kunming Institute of Zoology. All operations in this experiment adhered to animal ethics standards and were approved by the Ethics committe of Kunming Institute of Zoology,Chinese Academy of Sciences,with the ethical approval number IACUC-TE-2023-05-003.

Note that full information on the approval of the study protocol must also be provided in the manuscript.

Field-specific reporting

Please select the one below that is the best fit for your research. If you are not sure, read the appropriate sections before making your selection.

☒ Life sciences ☐ Behavioural & social sciences ☐ Ecological, evolutionary & environmental sciences

For a reference copy of the document with all sections, see nature.com/documents/nr-reporting-summary-flat.pdf

Life sciences study design

All studies must disclose on these points even when the disclosure is negative.

Sample size	All sample sizes were described in the figure legends, Given that tree shrews give birth to 2-3 offspring per litter. At least two biological replicates were used for each experiment.
Data exclusions	No data were excluded from the analyses.
Replication	We confirm that all experimental results have been repeated at least two times.as described in the Methods and figure legends.
Randomization	No randomization methods were used to determine how samples were allocated.This study mainly involves the comparison of different developmental stages of the same species.and comparisons of the same developmental period among different species.
Blinding	A single-blinded approach was adopted for statistical evaluation during the process of image data analysis.

Reporting for specific materials, systems and methods

We require information from authors about some types of materials, experimental systems and methods used in many studies. Here, indicate whether each material, system or method listed is relevant to your study. If you are not sure if a list item applies to your research, read the appropriate section before selecting a response.

Materials & experimental systems

n/a	Involved in the study
☐	☒ Antibodies
☐	☒ Eukaryotic cell lines
☒	☐ Palaeontology and archaeology
☐	☒ Animals and other organisms
☒	☐ Clinical data
☒	☐ Dual use research of concern
☒	☐ Plants

Methods

n/a	Involved in the study
☒	☐ ChIP-seq
☒	☐ Flow cytometry
☒	☐ MRI-based neuroimaging

Antibodies

Antibodies used

roduct Name,Vendor,Catalog Number
Rabbit Polyclonal anti-SATB2,Affinity Biosciences,DF292;
Rabbit Polyclonal anti-TBR1,Affinity Biosciences,DF2396;
Rabbit Polyclonal anti-TBR2/Eomes,Abcam,ab23345;
Rat Monoclonal anti-SOX2,Invitrogen,53-9811-82;
Rabbit Polyclonal anti-PTPRZ1,Sigma-Aldrich,HPA015103;
Mouse anti-BRN2 (1:500, Santa Cruz Biotechnology,sc-393324)
Mouse Monoclonal anti-SOX2 (L1D6A2)(1:500;Cell Signaling Technology, 4900T)

Alexa Fluor 594 AffiniPure Donkey Anti-Rabbit,Jackson ImmunoResearch Labs,711-585-152;
Alexa Fluor 594 AffiniPure Donkey Anti-Rat,Jackson ImmunoResearch Labs,712-585-153;
Alexa Fluor 488 AffiniPure Donkey Anti-Rabbit,Jackson ImmunoResearch Labs,711-545-152;
Alexa Fluor 488 AffiniPure Donkey Anti-Rat,Jackson ImmunoResearch Labs,712-545-150;

Validation

Antibodies were validated by the manufacture as described online. No further in-house validation was performed.
All Affinity Biosciences antibodies are validated and validation can be found at http://www.affbiotech.cn;
Validation of antibodies from Jackson ImmunoResearch can be found at http://www.jacksonimmuno.com;
Validation of antibodies from Invitrogen when available can be found at: http://www.invitrogen.com;
Validation of antibodies from Atlas Antibodies can be found at http://www.atlasantibodies.com;
Validation of antibodies from Abcam can be found at http://www.abcam.com;
validation of antibodies from Sigma-Aldrrich can be found at http://www.sigmaaldrich.com;

Eukaryotic cell lines

Policy information about cell lines and Sex and Gender in Research

Cell line source(s)

SH-SY5Y,a human neuroblastoma cell line, was purchased from the ATCC

Authentication

The SH-SY5Y cell line was purchased from ATCC and authenticated using STR analysis by company.

Mycoplasma contamination

All cell lines tested negative for mycoplasma contamination, and Mycoplasma removal agent were added during all cell cultures.

Commonly misidentified lines
(See ICLAC register)

n/a

Animals and other research organisms

Policy information about studies involving animals; ARRIVE guidelines recommended for reporting animal research, and Sex and Gender in Research

Laboratory animals

CD-1(ICR) mice were from laboratory animal center,Kunming institute of zoology;
Chinese tree shrew were from Primate Research Center,Kunming institute of zoology;

Wild animals

The study did not involve wild animals.

Reporting on sex

Lack of sex-based analysis because when analyzing data,the priority is consider the same batch coming from the same nest of embryos.

Field-collected samples

The study did not involve samples collected from field.

Ethics oversight

Animal research using Chinese tree shrews and mice was conducted according to the guidelines approved by the Animal Care and Use Committee of the Kunming institute of Zoology, CAS. The animals involved in this study were all from standard feeding environments.

Note that full information on the approval of the study protocol must also be provided in the manuscript.

Plants

Seed stocks	n/a
Novel plant genotypes	n/a
Authentication	n/a
