# Supplementary material for: Transcriptome of fetal cortex of tree shrew underlying the emergence of outer subventricular zone
Source: Commun Biol. 2026 Sep 14;9:1179. doi: 10.1038/s42003-026-10802-y (PMC13575156; doi:10.1038/s42003-026-10802-y)
Supplement: Supplementary file 2 [file 42003_2026_10802_MOESM13_ESM.pdf]

nature portfolio

Peer Review File

Transcriptome of Fetal Cortex of Tree Shrew Underlying the Emergence of Outer Subventricular Zone

Corresponding Author: Professor Lei Shi

This file contains all reviewer reports in order by version, followed by all author rebuttals in order by version.

Version 0:

Reviewer comments:

Reviewer #1

(Remarks to the Author)
In the manuscript "Transcriptome of Fetal Cortex of Tree Shrew Underlying the Emergence and Evolution of the Outer Subventricular Zone" by Hu et al., the authors characterize the OSVZ of the tree shrew and its developmental and evolutionary emergence using laser microdissection-based transcriptomics across cortical layers, combined with a comparative transcriptomic approach. The study identifies both shared and distinct gene expression signatures between the OSVZ of tree shrews and primates.
Furthermore, the authors highlight zinc finger proteins (ZNFs) with enriched expression in the OSVZ/iSVZ compared to the cortical plate, as a shared molecular feature between tree shrew and primate OSVZ. Functional analysis of one candidate, ZNF664, via overexpression in mouse demonstrates an increase in basal progenitor proliferation, suggesting a potential role in expanding the SVZ.
Overall, this study provides valuable insight into the molecular mechanisms underlying OSVZ evolution and represents an important contribution to understanding the origins of primate cortical complexity. The manuscript is well suited for publication in Communications Biology. I recommend publication after minor revision.

Main comments:

1. SATB2 is not specific to upper-layer neurons, as it also labels layer IV neurons and a subset of layer V neurons. To more accurately define upper-layer identity, the authors should include additional markers such as BRN2 (POU3F2) or CUX1/2.

2. The authors use SOX2 to label progenitors; however, this does not distinguish between progenitor subtypes. It would be important to clarify the composition of progenitors in the tree shrew OSVZ—specifically whether basal radial glia (bRG/oRG), intermediate progenitor cells (IPCs), or both are present. This point is particularly relevant given the presented single-cell RNA-seq data oft he OSVZ suggesting the absence of a clear IPC cluster in tree shrew OSVZ. Moreover, the ZNF664 overexpression experiments in mouse appear to increase IPC abundance. The authors should therefore include staining and quantification for IPCs (e.g., TBR2/EOMES) and compare these to bRG/oRG markers (e.g., HOPX).

3. The SOX2 staining pattern in the tree shrew cortex is somewhat unexpected, as SOX2-positive cells appear not only in germinal zones but also within the cortical plate. The authors should clarify whether this reflects biological differences, antibody specificity, or technical factors.

4. While the overexpression experiments are informative, complementary loss-of-function approaches (e.g., knockdown or knockout) would significantly strengthen the conclusions. Such experiments could test whether ZNF664 depletion reduces IPC abundance and affects OSVZ size or progenitor proliferation.

Reviewer #2

(Remarks to the Author)
Shi and colleagues aim to establish the tree shrew as a new and more suitable animal model to study the evolution of the human cortex, in particular to the formation and expansion of the OSVZ. This germinal zone is particularly large and expanded in primates, including human, but is also present in all the species with a folded cortex studied to far, which

reduces the strategic relevance of the tree shrew. Nevertheless, this animal is phylogenetically closer to primates than mouse and carnivores or ungulates, thus has some value in that respect. In this study, the authors perform several transcriptomic analyses, including layer-specific bulk transcriptomics, and single cell transcriptomics of combined germinal regions, of the embryonic cortex of tree shrew. They combine their results with public data from a number of species interesting to compare with, including human, macaque and mouse, to draw conclusions on conservation or divergence of gene expression, and potential functional relevance. They end by focusing on zinc-finger transcription factors, and test the relevance of one particular gene on the proliferation of mouse SVZ by in utero electroporation. The authors conclude that the tree shrew has potential for uncovering genetic and molecular bases of primate- and human-specific evolutionary features, including the mechanisms and cortical circuits underlying human OSVZ evolution and development. The study is original in its focus on a close relative to primates but with a smooth cortex and that yet develops and OSVZ. Experimentation is well conducted and analyses well presented, but they are rather naïve and insufficient to allow extracting several of the conclusions drawn by the authors, in particular their grand final conclusion indicated above.

The authors begin by arguing that the tree shrew exhibits an expanded OSVZ during cortical development, suggesting that SVZ expansion preceded the emergence primates. This is also true for other clades including carnivores and ungulates, as shown in Reillo et al 2011. Here this must be mentioned and this study cited. The key relevance of tree shrew is that it is lissencephalic, as mentioned in the next sentence, whereas all other species with OSVZ are gyrencephalic.

The sentence “These results suggest that NPC differentiation processes are initiated in the OSVZ around E37. – line 155”, is unclear. What does it exactly mean? OSVZ NPCs likely produce neurons since much earlier stages. If the authors mean that OSVZ NPCs self-consumption begins at E37 because they begin decreasing in number, this may be true overall, but it is very possible that it already occurs at earlier stages though only for a minority of NPCs, in which case the sentence is again not true. The authors must be more clear and specific with this conclusion.

In line 165, the authors state: “In the IZ, 404 genes were consistently highly expressed and were enriched in cell division, tube development, neurogenesis, and related processes” - What are these genes and what is the meaning of their expression in the proliferation-devoid IZ? Was this due to sampling contamination from OSVZ? The authors must clarify the functional meaning and relevance of these results.

Line 197: “Notably, several of these genes are implicated in human neurodevelopmental disorders, further supporting the utility of the tree shrew as
a novel animal model for investigating the molecular mechanisms underlying human brain diseases.” - These genes are also expressed in mouse cortical development, so this conclusion is not justified, especially given the significant disadvantages of tree shrew over mouse as experimental animal.

Line 251: “genes whose expression increased during OSVZ development (n = 240) were enriched in neurogenesis- and gliogenesis-related processes (Fig. 4b), indicating that OSVZ progenitors maintain differentiation-related programs at late stages of neurogenesis.” - Some of the top genes with developmental increase in expression in OSVZ are from the Notch pathway, which promotes progenitor proliferation. That contradicts this conclusion.

“PTPRZ1, TNC, TTYH1, TMEM132B, MT3, SLCO1C1, HOPX, MOD1, SLITRK2, EDNRB, PMP22 were highly expressed in RGCs (cluster 3), consistent with conserved OSVZ-associated progenitor identity (Fig. 5c).” - But were these RGCs in VZ (aRGCs), ISVZ or OSVZ (bRGCs)?

It is well known and established that HOPX is not a marker of OSVZ across development and species. It is highly enriched in OSVZ only at late stages of human cortex, but not at early stages of human, nor at any particular age in other species, like ferret, or the tree shrew like the authors find here. The authors’ us of HOPX as a classical OSVZ marker is flawed.

One of the main conclusions of this study is that “Together, these results demonstrate the conservation of a shared molecular toolkit underlying OSVZ progenitor identity in tree shrews and primates.”. This molecular identity may also be share with other non-primate species with OSVZ, like ungulates (pig, sheep) and carnivores (cat, ferret, dog). This conclusion must be extended to these other species unless the authors compare expressions in tree shrew with other species.

The conclusion that “conserved and primate-enriched molecular
programs in basal progenitor zones provide a framework for identifying regulatory factors involved in OSVZ evolution” is trivial. This conclusion is based on comparisons made between progenitor cells and neurons, and thus findings do not favor identification of regulatory factors involved in OSVZ evolution. That will be achieved only if the authors identify factors driving the formation of OSVZ cells prior to OSVZ existence, and maintenance of OSVZ progenitors without differentiation. The comparisons performed in this study do not allow reaching these conclusions.

In the in utero electroporation experiments, the authors find significant increases in the proportions of Tbr2+ cells, and then argue that these results demonstrate that ZNF664 promotes the proliferation of basal progenitor cells, particularly IPCs. But the authors do not demonstrate that those Tbr2+ cells are proliferating and undergoing mitotic division. Maybe the effect of ZNF664 is to block cell cycle progression in Tbr2+ cells, and hence they accumulate instead of progressing to produce neurons.

Finally, the conclusions from the experiments in N2A cells are flawed, because N2A cells are of cancer origin, and hence their gene regulation mechanisms may be completely different and unrelated to those in cortical progenitor cells. If the authors want to investigate this, they should do this kind of experiments in the cortex, for example in utero electroporating

ZNF664 and then FACS-sorting electroporated cells and analyzing their transcriptomic changes.

Reviewer #3

(Remarks to the Author)
In this paper, the authors focused on the development of the cerebral cortex in the tree shrew, a unique lineage among mammals. Although gyri do not form in the cerebral cortex of this animal, a distinct outer subventricular zone (OSVZ) was observed, unlike in rodents such as mice. Transcriptome analysis combining techniques such as laser microtome sectioning revealed specific changes in gene expression at different developmental stages. Furthermore, the changes in the expression of gene clusters associated with the extracellular matrix are of particular interest in relation to the OSVZ. Finally, through a comparison of gene expression shared with the primate OSVZ, the authors identified the ZNF664 gene, and overexpression of this gene in the mouse cerebral cortex increased the number of precursor cells in the subventricular zone. In summary, the authors have succeeded in identifying the molecular mechanisms involved in brain enlargement, which are common to all mammals, through a comparative developmental study of the cerebral cortex in distinct mammalian lineages.

Minor comment:

Figure 1 compares adult brains between mice and tree shrews, but to what extent do the brain sizes of tree shrews and mice differ immediately after birth? It would also be helpful to have a schematic comparing the developmental timelines of the cerebral cortex in mice, tree shrews, and primates.

In Figure 7, it appears that the proportion of GFP-positive cells invade the cortical plate is lower in the cerebral cortex where znf664 is overexpressed compared to the control. Is this actually due to a delay in neuronal differentiation or migration caused by the overexpression of this gene? It is necessary to quantify the proportion of GFP-positive cells in different bins/layers of the cerebral cortex, or the proportion of cells that have exited the cell cycle.

Version 1:

Reviewer comments:

Reviewer #1

(Remarks to the Author)
I appreciate the authors' thorough revision. All of my previous comments have been adequately addressed, and I have no further suggestions.
I recommend acceptance of the manuscript.

Reviewer #3

(Remarks to the Author)
All concerns raised by the reviewer have been appropriately addressed.

Reviewer #4

(Remarks to the Author)
The authors successfully replied to all comments. All changes improve the manuscript by strengthening the robustnesss of data and by lowering down the tone of some unsupported conclusions.

Open Access This Peer Review File is licensed under a Creative Commons Attribution 4.0 International License, which permits use, sharing, adaptation, distribution and reproduction in any medium or format, as long as you give appropriate credit to the original author(s) and the source, provide a link to the Creative Commons license, and indicate if changes were made.
In cases where reviewers are anonymous, credit should be given to 'Anonymous Referee' and the source.
The images or other third party material in this Peer Review File are included in the article's Creative Commons license, unless indicated otherwise in a credit line to the material. If material is not included in the article's Creative Commons license and your intended use is not permitted by statutory regulation or exceeds the permitted use, you will need to obtain permission directly from the copyright holder.
To view a copy of this license, visit https://creativecommons.org/licenses/by/4.0/

<u>Reviewers' comments:</u>

Reviewer #1 (Remarks to the Author):

In the manuscript "Transcriptome of Fetal Cortex of Tree Shrew Underlying the Emergence and Evolution of the Outer Subventricular Zone" by Hu et al., the authors characterize the OSVZ of the tree shrew and its developmental and evolutionary emergence using laser microdissection-based transcriptomics across cortical layers, combined with a comparative transcriptomic approach. The study identifies both shared and distinct gene expression signatures between the OSVZ of tree shrews and primates.
Furthermore, the authors highlight zinc finger proteins (ZNFs) with enriched expression in the OSVZ/iSVZ compared to the cortical plate, as a shared molecular feature between tree shrew and primate OSVZ. Functional analysis of one candidate, ZNF664, via overexpression in mouse demonstrates an increase in basal progenitor proliferation, suggesting a potential role in expanding the SVZ.
Overall, this study provides valuable insight into the molecular mechanisms underlying OSVZ evolution and represents an important contribution to understanding the origins of primate cortical complexity. The manuscript is well suited for publication in Communications Biology. I recommend publication after minor revision.
> We thank the reviewer for the careful assessment and positive and constructive comments. We are extremely happy that reviewer acknowledge that our work provides *"valuable insight into the molecular mechanisms underlying OSVZ evolution and represents an important contribution to understanding the origins of primate cortical complexity".* We have addressed the concerns, performed additional experiments and analyses, and carefully revised the manuscript while adding new results, additional methodological details, and revised figures. We believe that these changes have substantially improved the manuscript and thank the reviewer again.

Main comments:

1. SATB2 is not specific to upper-layer neurons, as it also labels layer IV neurons and a subset of layer V neurons. To more accurately define upper-layer identity, the authors should include additional markers such as BRN2 (POU3F2) or CUX1/2.
> We thank the reviewer for this point.
1) Following reviewer suggestions, we performed both CUX1 and BRN2 staining. However, CUX1 antibody didn't work in the tree shrew, and we added the BRN2 results as shown in revised **Fig.2**. We observed that the number of BRN2 cells continued to increase as well as that of SATB2 cells (**Fig. 2e**), however BRN2 cells were still in migration process and not restricted in the

upper layers during cortical development (**Fig. 2c**).
2) The number of TBR1 cells first decrease from E28 to E37, and then remain a comparable level from E37 to E42 (**Fig. 2e**).
3) We kept the SATB2 results in the revised text, which may be useful for other researchers in their future experiments.
These results have been added to the revised **Fig. 2**.

2. The authors use SOX2 to label progenitors; however, this does not distinguish between progenitor subtypes. It would be important to clarify the composition of progenitors in the tree shrew OSVZ—specifically whether basal radial glia (bRG/oRG), intermediate progenitor cells (IPCs), or both are present. This point is particularly relevant given the presented single-cell RNA-seq data oft he OSVZ suggesting the absence of a clear IPC cluster in tree shrew OSVZ. Moreover, the ZNF664 overexpression experiments in mouse appear to increase IPC abundance. The authors should therefore include staining and quantification for IPCs (e.g., TBR2/EOMES) and compare these to bRG/oRG markers (e.g., HOPX).
> We thank the reviewer for this constructive point.
1) As suggested by the reviewer, we have performed triple immunostaining using antibodies against SOX2, PTPRZ1, and IPCs marker TBR2 (**Fig. 5g)**. Quantification analysis suggested that in OSVZ, 56.815% of the progenitors belonged to IPCs ($SOX2^-PTPRZ1^-TBR2^+$; **Fig. 5g**, green arrow), while pan-RGCs ($SOX2^+PTPRZ1^-TBR2^-$; **Fig. 5g**, grey arrow) and oRGCs ($SOX2^+PTPRZ1^+TBR2^-$; **Fig. 5g**, orange arrow) constituted 21.172% and 21.012% respectively (**Fig. 5h).**

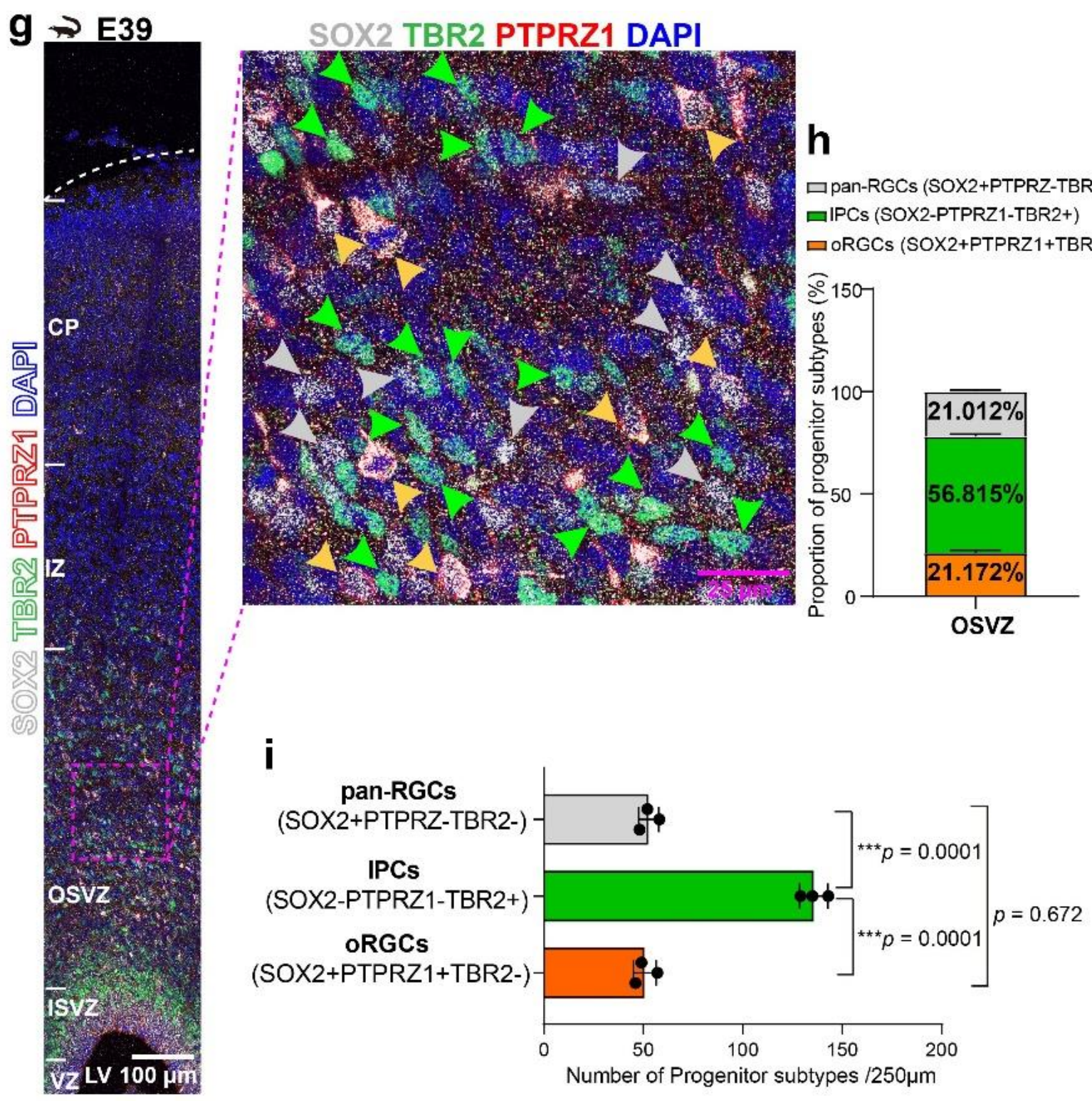

2) We previously did not find a clear IPCs cluster (**Fig.5 a,b**). However, during revision, we performed a subcluster analysis with cluster 3 and cluster 4, and identified an IPCs cluster (**Fig. 5c**). The new subcluster analysis results successfully classified cluster 3 and 4 into apical RGCs, basal RGCs, Intermediate progenitor cells (IPCs) and other cells types (**Fig. 5d**).

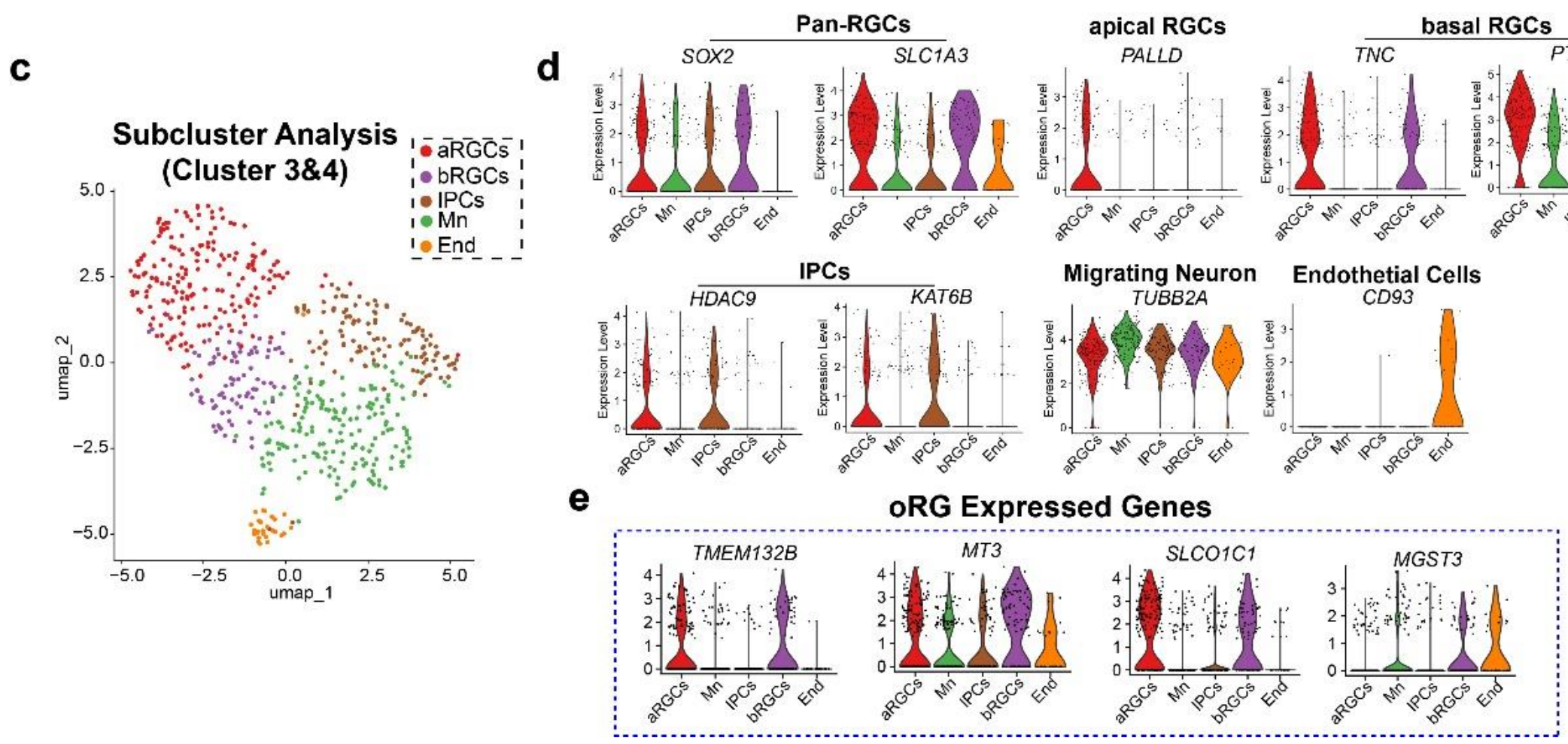

We have added these results to the revised **Fig. 5** and also related descriptions in the revised text.

3) Following reviewer's suggestions, we performed bRGCs marker PTPRZ1 staining in the ZNF664 overexpression experiments. However, PTPRZ1 or HOPX staining doesn't work in the mouse embryo. As an alternative solution, we performed RGCs marker SOX2 staining, and the results indicated that ZNF664 expression didn't increase the proportion of SOX2+ (**Fig. 7j**).

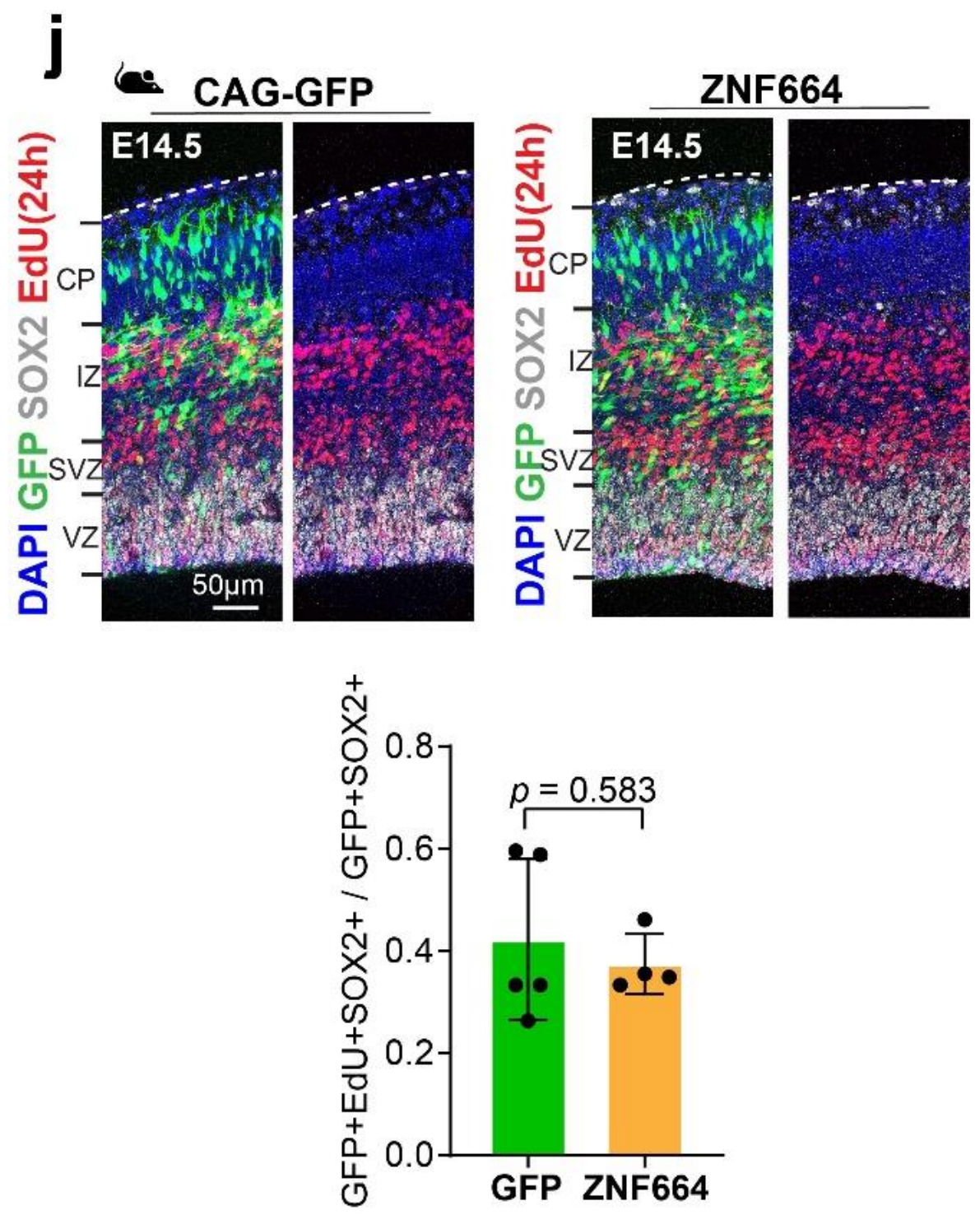

Therefore, we revised our conclusions and only emphasized that ZNF664 could

only promote IPCs proliferation. We also discuss that future experiments using primate brain organoids or tree shrew IUE to investigate functional effects of ZNF664 on basal RGCs.
These results have been added to the revised **Fig. 7** and related descriptions in the revised text.

3. The SOX2 staining pattern in the tree shrew cortex is somewhat unexpected, as SOX2-positive cells appear not only in germinal zones but also within the cortical plate. The authors should clarify whether this reflects biological differences, antibody specificity, or technical factors.
> We thank the reviewer for raising this point.
1) We performed additional SOX2 staining to address this. We tried a different SOX2-antibody (CST, Cell Signaling Technology, 4900T;1:500), as well as the previously used SOX2-antibody (Invitrogen, 53-9811-82;1:500). We performed SOX2-immunostaining at E28, E37 and E42 (**Supplementary Fig. 2a**). Quantitative analysis suggested SOX2+ progenitor cells exhibited the same trends as before, reaching the peak at E37 (**Fig. 2e and Supplementary Fig. 2b**). The number of SOX2+ cells was slightly higher for the Invitrogen antibody than from CST (**Supplementary Fig. 2b**).
2) Regardless, both antibodies demonstrate low proportions of SOX2+ progenitors in the CP compared to in VZ, ISVZ and OSVZ (**Supplementary Fig. 2c**).
3) We reviewed published studies, and from the published figures, observed that the following published studies also showed some cells expressing SOX2 in the cortical plate at embryonic stage.
Ref: 43 Kostic, M. et al. YAP Activity Is Necessary and Sufficient for Basal Progenitor Abundance and Proliferation in the Developing Neocortex. Cell Rep 27, 1103-1118 e1106 (2019). https://doi.org/10.1016/j.celrep.2019.03.091
44 Gonzalez-Bohorquez, D. et al. FASN-dependent de novo lipogenesis is required for brain development. Proc Natl Acad Sci U S A 119 (2022). https://doi.org/10.1073/pnas.2112040119
45 Shi, L., Qalieh, A., Lam, M. M., Keil, J. M. & Kwan, K. Y. Robust elimination of genome-damaged cells safeguards against brain somatic aneuploidy following Knl1 deletion. Nat Commun 10, 2588 (2019). https://doi.org/10.1038/s41467-019-10411-w

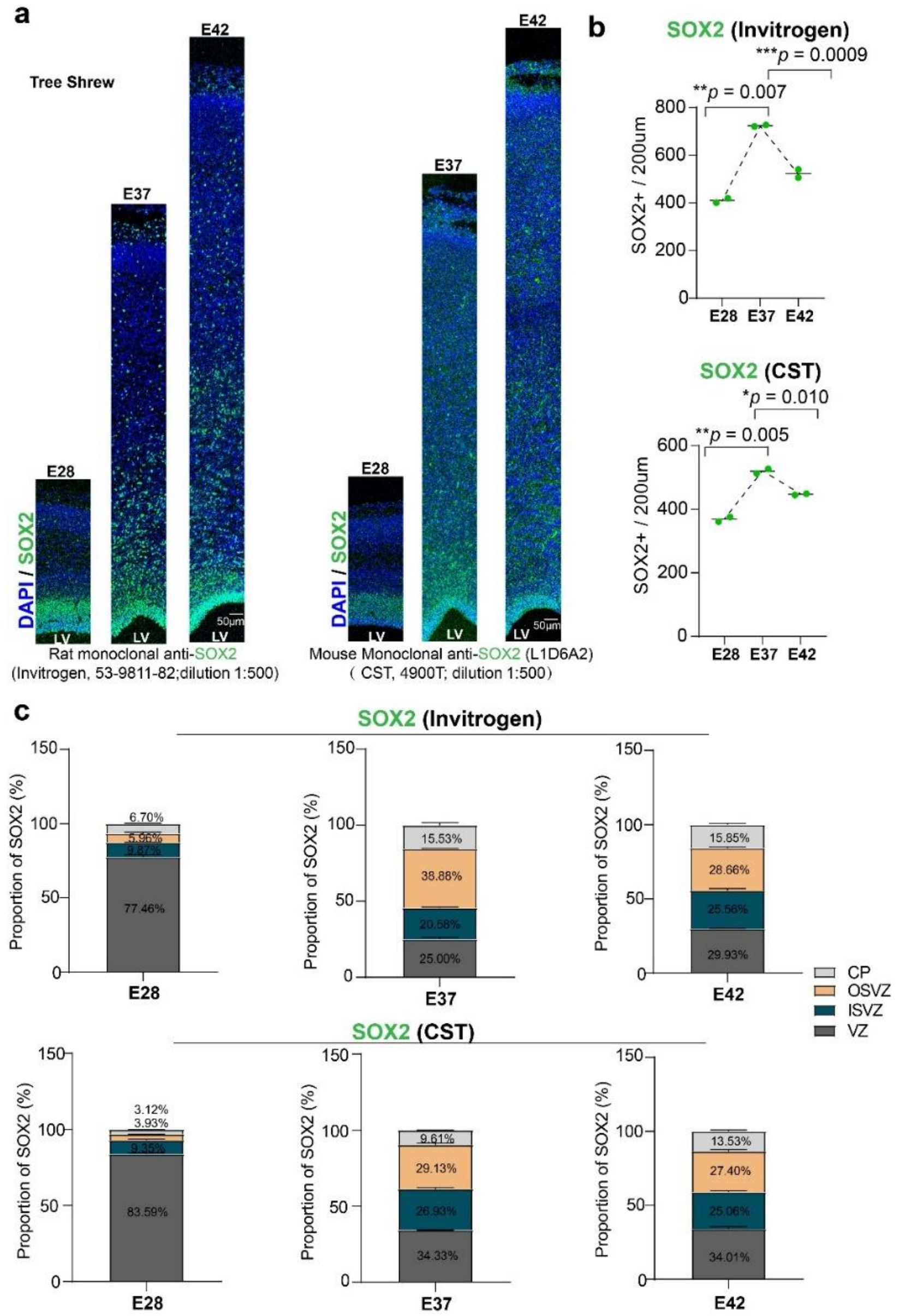

Overall, these results suggest that SOX2+ cells in the cortical plate of tree shrew may represent a true biological pattern, and antibody specificity may account for the intensity of immune reactions, which could contribute to some potential false positives.

We have added these results as a new figure **Supplementary Fig. 2** and also related descriptions in the revised text.

4. While the overexpression experiments are informative, complementary loss-of-function approaches (e.g., knockdown or knockout) would significantly strengthen the conclusions. Such experiments could test whether ZNF664 depletion reduces IPC abundance and affects OSVZ size or progenitor proliferation.

> We understand reviewer point and thanks for this suggestion.

1) ZNF664 is only expressed in tree shrew and primates. We could not perform

knockdown experiments in the tree shrew embryos or brain organoids from primates, as such experiments require extensive resources and are beyond the scope of our current work. We hope to expand to this research direction with future funding and timeline. We point our future direction as follows

"*future experiments are warranted to investigate whether ZNF664 can also promote proliferation basal RGCs using primate brain organoids or tree shrew IUE experiments*." in the revised text **Page 21.**

2) As a complementary approach, we performed knock-down experiments using siRNA targeting human *ZNF664* in human *SH-SY5Y* cells, and the quantification results showed that knockdown ZNF664 can significantly reduce the proliferating cells $EdU^{+}GFP^{+}$ (**Supplementary Fig. 9c**).

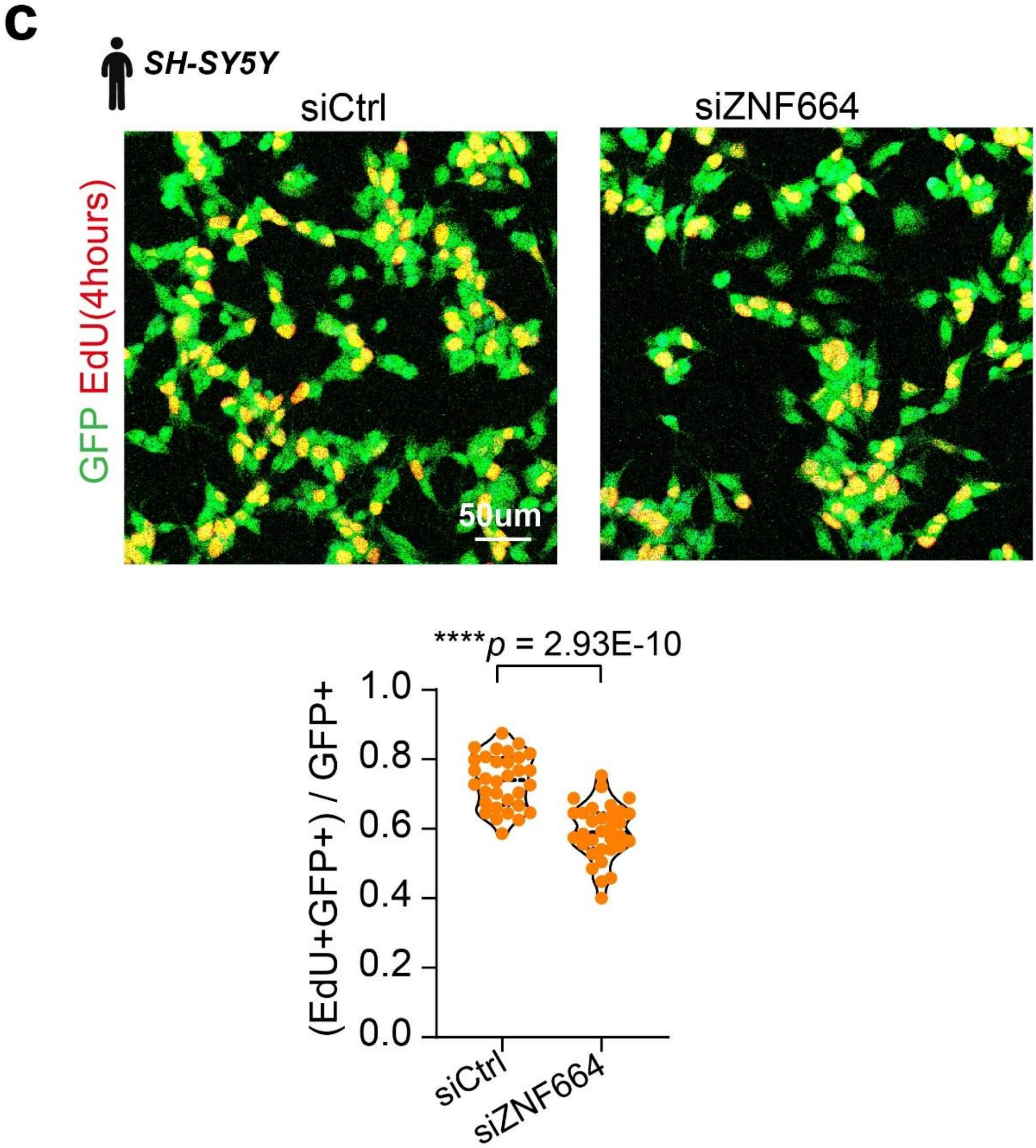

Reviewer #2 (Remarks to the Author):

Shi and colleagues aim to establish the tree shrew as a new and more suitable animal model to study the evolution of the human cortex, in particular to the formation and expansion of the OSVZ. This germinal zone is particularly large and expanded in primates, including human, but is also present in all the species with a folded cortex studied to far, which reduces the strategic relevance of the tree shrew. Nevertheless, this animal is phylogenetically closer to primates than mouse and carnivores or ungulates, thus has some value in that respect. In this study, the authors perform several transcriptomic analyses, including layer-specific bulk transcriptomics, and single cell transcriptomics of combined germinal regions, of the embryonic cortex of tree shrew. They combine their results with public data from a number of species interesting to compare with, including human, macaque and mouse, to draw conclusions on conservation or divergence of gene expression, and potential functional relevance. They end by focusing on zinc-finger transcription factors, and test the relevance of one particular gene on the proliferation of mouse SVZ by in utero electroporation. The authors conclude that the tree shrew has potential for uncovering genetic and molecular bases of primate- and human-specific evolutionary features, including the mechanisms and cortical circuits underlying human OSVZ evolution and development. The study is original in its focus on a close relative to primates but with a smooth cortex and that yet develops and OSVZ. Experimentation is well conducted and analyses well presented, but they are rather naïve and insufficient to allow extracting several of the conclusions drawn by the authors, in particular their grand final conclusion indicated above.

> We thank the reviewer for the careful evaluation of our work and for the positive and constructive comments. We are encouraged by the reviewer's comment that "*The study is original in its focus on a close relative to primates but with a smooth cortex and that yet develops and OSVZ*", and also "*Experimentation is well conducted and analyses well presented*". In response to the concerns raised, we have performed substantial revisions to our conclusions and have carefully revised the manuscript. These changes include the extending the OSVZ conclusions to other species as suggested (cited *Reillo et al 2011*), revision of figures, subcluster analysis of single cell datasets, and more results from ZNF664 IUE experiments. We believe these revisions have substantially improved the manuscript and thank the reviewer again for their valuable input.

The authors begin by arguing that the tree shrew exhibits an expanded OSVZ during cortical development, suggesting that SVZ expansion preceded the emergence primates. This is also true for other clades including carnivores and ungulates, as shown in Reillo et al 2011. Here this must be mentioned and this study cited. The key relevance of tree shrew is that it is lissencephalic, as mentioned in the next sentence, whereas all other species with OSVZ are

gyrencephalic.

> We appreciate the reviewer for raising this important point. We revised our manuscript to more accurately and rigorously discuss these points and added the *Reillo et al 2011* citation. The sentence in question on **page 4** has been amended to read: "*Intriguingly, the tree shrew and other clades including carnivores and ungulates exhibit an expanded OSVZ during cortical development* [39-41]*, suggesting that SVZ expansion preceded the emergence of primates.*"

The sentence "These results suggest that NPC differentiation processes are initiated in the OSVZ around E37. – line 155", is unclear. What does it exactly mean? OSVZ NPCs likely produce neurons since much earlier stages. If the authors mean that OSVZ NPCs self-consumption begins at E37 because they begin decreasing in number, this may be true overall, but it is very possible that it already occurs at earlier stages though only for a minority of NPCs, in which case the sentence is again not true. The authors must be more clear and specific with this conclusion.

> We thank the reviewer for identifying this inaccuracy. We agree that our original phrasing was incorrect, and have revised the manuscript accordingly to emphasize its rigor and clarity. The sentence in question on **page 8** has been amended to read: "*These findings suggest that NPC proliferation processes were reaching the peak in the OSVZ around E37, while NPCs differentiation processes may have already initiated at earlier stage*."

In line 165, the authors state: "In the IZ, 404 genes were consistently highly expressed and were enriched in cell division, tube development, neurogenesis, and related processes" - What are these genes and what is the meaning of their expression in the proliferation-devoid IZ? Was this due to sampling contamination from OSVZ? The authors must clarify the functional meaning and relevance of these results.

> We really appreciate the reviewer for raising this point.

1) As reviewer pointed out that IZ is very close to the OSVZ, and it is challenging to dissect an intact IZ without contamination from OSVZ.

2) Therefore, we performed analysis by explicitly filtering out OSVZ-expressed genes, and identified 85 genes as exclusively IZ-expressed genes, and found that they were enriched for tube development, cell-cell adhesion et al processes, consistent with the roles of neuron migration (**Fig. 2g** and **Supplementary Data 5**).

3) By filtering out OSVZ-expressed genes, we also updated the list of IZ genes that exhibit linear increases or decreases of expression during development, as shown in **Fig. 3e.**

We have added these results to the revised **Fig. 2, Fig. 3e** and also related descriptions in the revised text.

Line 197: "Notably, several of these genes are implicated in human neurodevelopmental disorders, further supporting the utility of the tree shrew as a novel animal model for investigating the molecular mechanisms underlying human brain diseases." - These genes are also expressed in mouse cortical development, so this conclusion is not justified, especially given the significant disadvantages of tree shrew over mouse as experimental animal.

> We appreciated the reviewer for these points. We agreed with the reviewer that these genes are also expression during mouse cortical development, therefore, we removed our previous conclusions in the revised manuscript.

See **page 10**.

Line 251: "genes whose expression increased during OSVZ development (n = 240) were enriched in neurogenesis- and gliogenesis-related processes (Fig. 4b), indicating that OSVZ progenitors maintain differentiation-related programs at late stages of neurogenesis." - Some of the top genes with developmental increase in expression in OSVZ are from the Notch pathway, which promotes progenitor proliferation. That contradicts this conclusion.

> We appreciated the reviewer for this point. We agreed with reviewer point that *NOTCH2* and *NOTCH3* are from the Notch pathway and their expression are developmental increased. Therefore, we rewrote the relevant text as follows:" *By contrast, genes whose expression increased during OSVZ development (n = 240) were enriched in neurogenesis- and gliogenesis-related processes (Fig. 4b). In addition, the expression of NOTCH2 and NOTCH3 from NOTCH pathway*[45] *is developmental increased (Fig. 4d, blue color), indicating that OSVZ progenitors maintain proliferation-related programs at late stages of neurogenesis.*"

See **page 14**.

"PTPRZ1, TNC, TTYH1, TMEM132B, MT3, SLCO1C1, HOPX, MOD1, SLITRK2, EDNRB, PMP22 were highly expressed in RGCs (cluster 3), consistent with conserved OSVZ-associated progenitor identity (Fig. 5c)." - But were these RGCs in VZ (aRGCs), ISVZ or OSVZ (bRGCs)?

> We thank the reviewer for this constructive point.

To further identify RGCs types, we performed an additional subcluster analysis using cluster 3 and cluster 4, both of which exhibit increased expression of SOX2 compared to other clusters (**Fig. 5b**). This sub-cluster analysis successfully further classified cluster 3 and 4 into apical RGCs, basal RGCs, Intermediate progenitor cells (IPCs) and other cells types (**Fig. 5c,d**). We further presented oRGCs-expressed genes including TNC, PTPRZ1, et al, as shown in (**Fig. 5d,e and Supplementary Fig. 6**). Notably, 4 genes (*EEPD1*, *FAM107A*, *BMP7*, *TKTL1*) were not detected in the tree shrew bRGCs (**Supplementary Fig. 6b**)

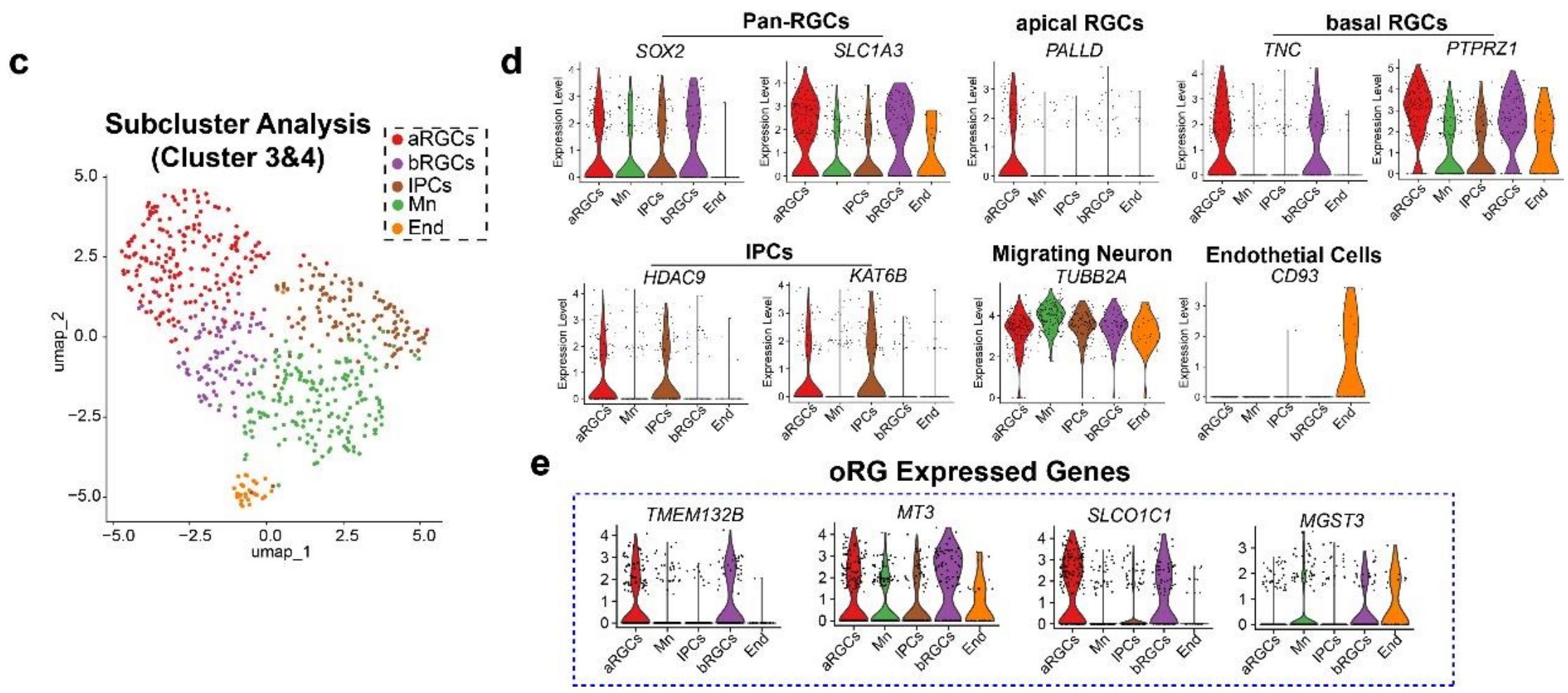

We have added these results to the revised **Fig. 5** and also related descriptions in the revised text.

It is well known and established that HOPX is not a marker of OSVZ across development and species. It is highly enriched in OSVZ only at late stages of human cortex, but not at early stages of human, nor at any particular age in other species, like ferret, or the tree shrew like the authors find here. The authors' us of HOPX as a classical OSVZ marker is flawed.

> We understand reviewer point and really appreciate for this point.
Following reviewer suggestion, we removed HOPX immunostaining results and related descriptions in the revised manuscript. See **page 15** and revised **Fig. 5**.

One of the main conclusions of this study is that "Together, these results demonstrate the conservation of a shared molecular toolkit underlying OSVZ progenitor identity in tree shrews and primates". This molecular identity may also be share with other non-primate species with OSVZ, like ungulates (pig, sheep) and carnivores (cat, ferret, dog). This conclusion must be extended to these other species unless the authors compare expressions in tree shrew with other species.

> We appreciated the reviewer for these points. We agreed with the reviewer that this molecular toolkit may be shared by other species, therefore, we have modified our conclusions in the revised manuscript as below: "*Together, these results demonstrate that a shared molecular toolkit underlying OSVZ progenitor identity is conserved across tree shrews and primates, and potentially in other gyrencephalic mammals such as ungulates (pig, sheep) and carnivores (cat, ferret, dog).*"
See **page 17**.

The conclusion that "conserved and primate-enriched molecular programs in basal progenitor zones provide a framework for identifying regulatory factors involved in OSVZ evolution" is trivial. This conclusion is based on comparisons made between progenitor cells and neurons, and thus findings do not favor

identification of regulatory factors involved in OSVZ evolution. That will be achieved only if the authors identify factors driving the formation of OSVZ cells prior to OSVZ existence, and maintenance of OSVZ progenitors without differentiation. The comparisons performed in this study do not allow reaching these conclusions.

> We understand and really appreciated for these points. Following reviewer suggestions, we removed the words about regulator factors. The new conclusion is as follows: "*Together, these results highlight conserved and primate-enriched molecular programs in basal progenitor zones.*"

See **page 19**.

In the in utero electroporation experiments, the authors find significant increases in the proportions of Tbr2+ cells, and then argue that these results demonstrate that ZNF664 promotes the proliferation of basal progenitor cells, particularly IPCs. But the authors do not demonstrate that those Tbr2+ cells are proliferating and undergoing mitotic division. Maybe the effect of ZNF664 is to block cell cycle progression in Tbr2+ cells, and hence they accumulate instead of progressing to produce neurons.

> We appreciated the reviewer for these points.

Following reviewer suggestions, we performed another independent IUE experiments, and the results suggested that ZNF664 expression can promote IPCs proliferation in the mouse based on the EdU (**Fig. 7h**, right panel; EdU+GFP+TBR2+/GFP+ TBR2+ from 20.12% to 34.98%, unpaired t-test, p = 0.010), and Ki67 experiments (**Fig. 7i**, right panel; Ki67+GFP+TBR2+/GFP+ TBR2+ from 68.12% to 86.28%, unpaired t-test, p = 0.009).

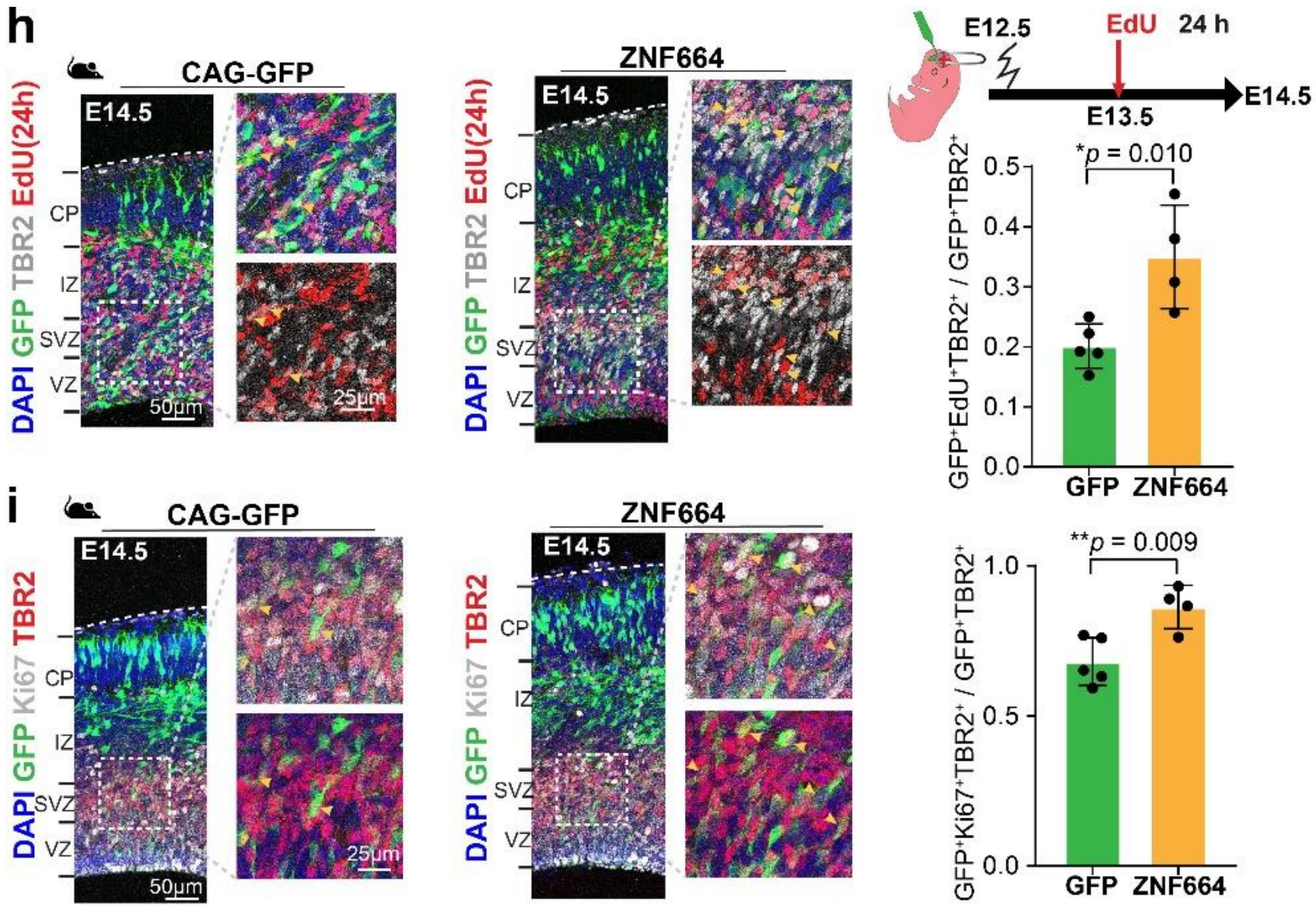

These results have been added to the revised **Fig. 7** and related descriptions in the revised text.

Finally, the conclusions from the experiments in N2A cells are flawed, because

N2A cells are of cancer origin, and hence their gene regulation mechanisms may be completely different and unrelated to those in cortical progenitor cells. If the authors want to investigate this, they should do this kind of experiments in the cortex, for example in utero electroporating ZNF664 and then FACS-sorting electroporated cells and analyzing their transcriptomic changes.

> We agreed and appreciated the reviewer for these points. We removed our N2A result in the revised text, and the molecular mechanism about ZNF664 will be investigated in our future experiments.

See **page 20-21**.

Reviewer #3 (Remarks to the Author):

In this paper, the authors focused on the development of the cerebral cortex in the tree shrew, a unique lineage among mammals. Although gyri do not form in the cerebral cortex of this animal, a distinct outer subventricular zone (OSVZ) was observed, unlike in rodents such as mice. Transcriptome analysis combining techniques such as laser microtome sectioning revealed specific changes in gene expression at different developmental stages. Furthermore, the changes in the expression of gene clusters associated with the extracellular matrix are of particular interest in relation to the OSVZ. Finally, through a comparison of gene expression shared with the primate OSVZ, the authors identified the ZNF664 gene, and overexpression of this gene in the mouse cerebral cortex increased the number of precursor cells in the subventricular zone. In summary, the authors have succeeded in identifying the molecular mechanisms involved in brain enlargement, which are common to all mammals, through a comparative developmental study of the cerebral cortex in distinct mammalian lineages.
> We thank the reviewer for the careful assessment, positive and constructive comments. We are encouraged by the reviewer acknowledge that our work "*succeeded in identifying the molecular mechanisms involved in brain enlargement*". We have addressed the concerns, performed additional analyses and experiments, and carefully revised the manuscript. We believe these changes have substantially improved the manuscript and thank the reviewer again.

Minor comment:

Figure 1 compares adult brains between mice and tree shrews, but to what extent do the brain sizes of tree shrews and mice differ immediately after birth? It would also be helpful to have a schematic comparing the developmental timelines of the cerebral cortex in mice, tree shrews, and primates.
> We thank the reviewer for these points.
1) We measured the cortical size in the tree shrews and mice respectively at postnatal day 0 (P0), and the results showed that the cortical size of tree shrew is about 3.47-fold of the mouse (**Fig. 1e**).

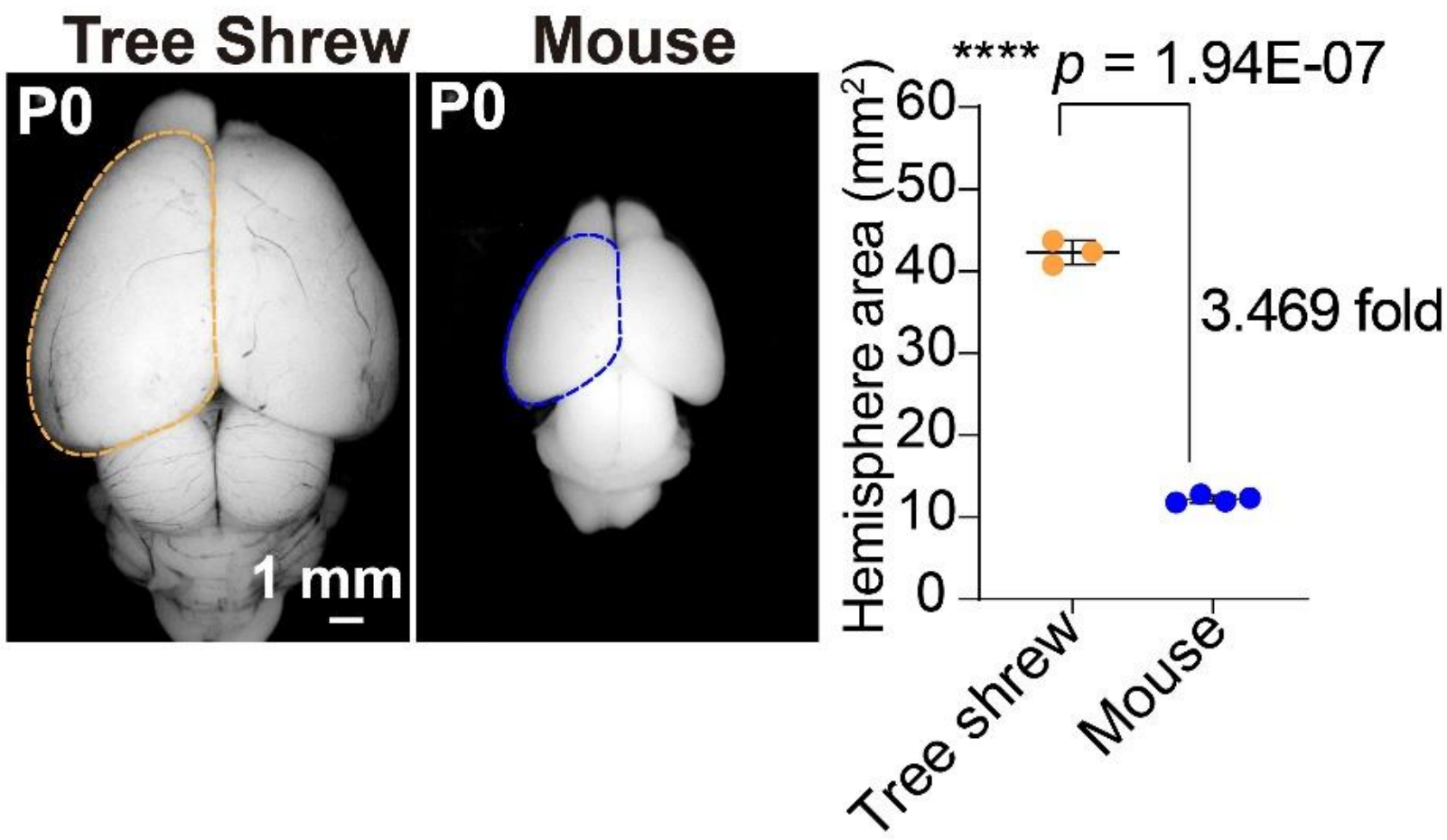

2) Following reviewer's suggestions, we made a schematic to compare the developmental timelines of the cerebral cortex among mice, tree shrews, macaques and human (**Fig. 1d**).

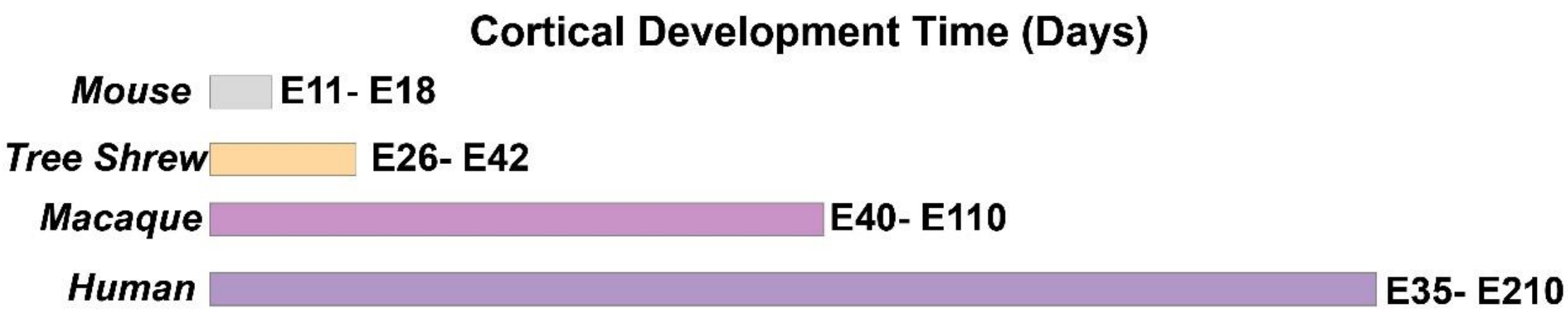

These results have been added to the revised **Fig. 1**.

In Figure 7, it appears that the proportion of GFP-positive cells invade the cortical plate is lower in the cerebral cortex where znf664 is overexpressed compared to the control. Is this actually due to a delay in neuronal differentiation or migration caused by the overexpression of this gene? It is necessary to quantify the proportion of GFP-positive cells in different bins/layers of the cerebral cortex, or the proportion of cells that have exited the cell cycle.

> We thank the reviewer for this point.
Following reviewer's suggestions, we quantified GFP-positive cells in different cortical layers, and the results showed that after expression of ZNF664, the proportion of GFP^+ cells was significantly increased in the VZ and IZ, but decreased in the CP. Combined with the results of promoting progenitor proliferation by ZNF664, GFP^+ quantification result suggested a delay in neuron differentiation after ZNF664 expression (**Fig. 7g)**.
These results have been added to the revised **Fig. 7**.

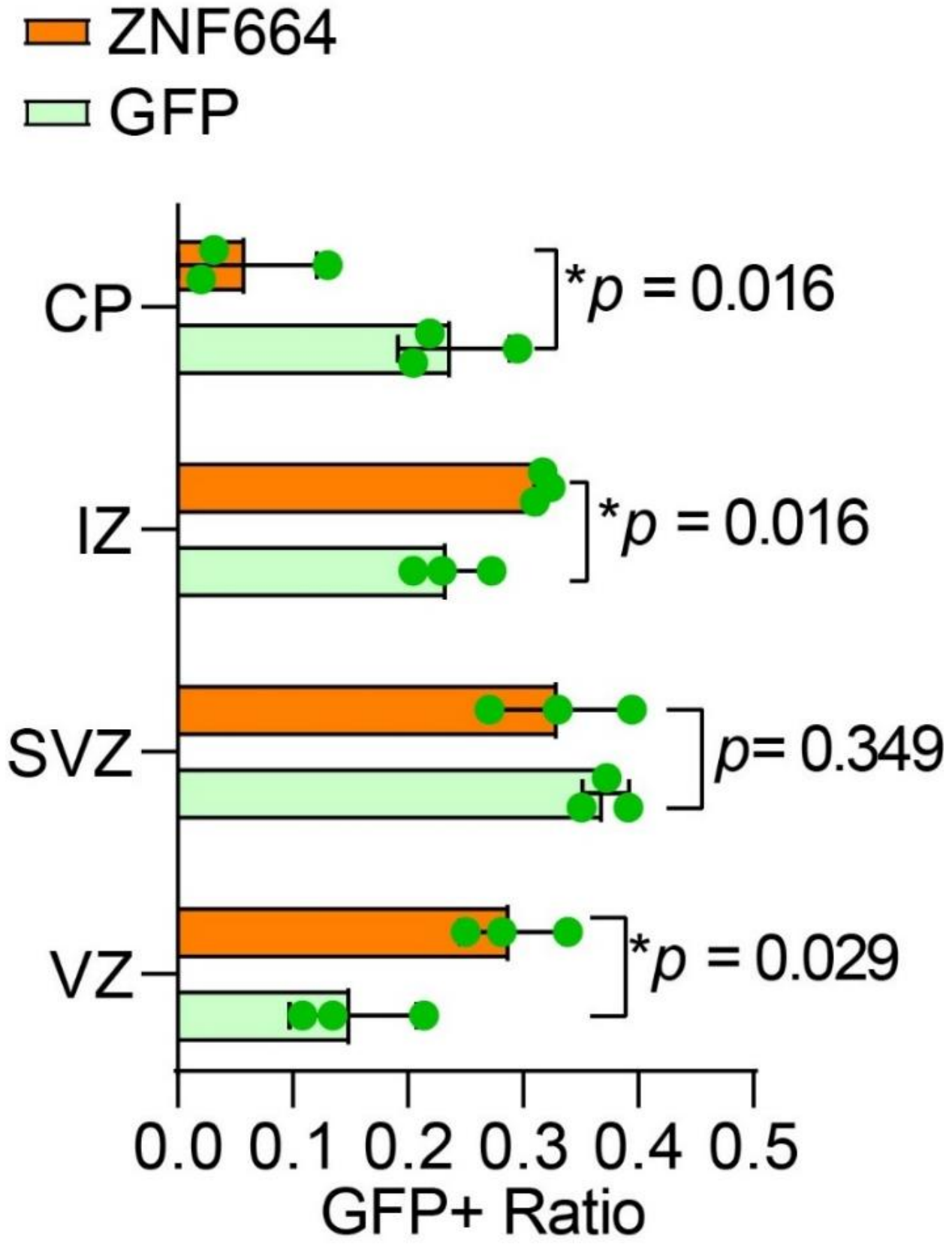
ZNF664
GFP
CP
IZ
SVZ
VZ
*p = 0.016
*p = 0.016
p= 0.349
*p = 0.029
0.0 0.1 0.2 0.3 0.4 0.5
GFP+ Ratio

Reviewers' comments:

Reviewer #1 (Remarks to the Author):

I appreciate the authors' thorough revision. All of my previous comments have been adequately addressed, and I have no further suggestions.

I recommend acceptance of the manuscript.

>We thank Reviewer #1 for the careful assessment and constructive comments throughout the review process. We are glad that the revisions have addressed all their concerns.

Reviewer #3 (Remarks to the Author):

All concerns raised by the reviewer have been appropriately addressed.

> We thank Reviewer #3 for their careful assessment, helpful comments, and positive feedback, which have significantly improved our manuscript. We are very happy to see that the revisions have addressed the remaining concerns.

Reviewer #4 (Remarks to the Author):

The authors successfully replied to all comments. All changes improve the manuscript by strengthening the robustnesss of data and by lowering down the tone of some unsupported conclusions.

> We thank Reviewer #2 and Reviewer #4 for their careful assessment, constructive comments, and positive feedback, which have greatly improved our manuscript. We're pleased that the revisions have resolved all remaining concerns.
