# Supplementary material for: Transcriptome of fetal cortex of tree shrew underlying the emergence of outer subventricular zone
Source: Commun Biol. 2026 Sep 14;9:1179. doi: 10.1038/s42003-026-10802-y (PMC13575156; doi:10.1038/s42003-026-10802-y)
Supplement: Supplementary file 3 [file 42003_2026_10802_MOESM1_ESM.pdf]

Supplementary information

Transcriptome of Fetal Cortex of Tree Shrew Underlying the Emergence of Outer Subventricular Zone

Ting Hu[1,2,4], Yangxuan Wen[5], Yulian Tan[1,2,3], Yifan Kong[1,2,4], Ling Yuan[5], Soojin V. Yi [6,7,8], Lei Shi[1,2,3*]

[1]State Key Laboratory of Genetic Evolution & Animal Models, Kunming Institute of Zoology, Chinese Academy of Sciences, Kunming, Yunnan, P.R. China
[2]Yunnan Key Laboratory of Animal Models and Human Disease Mechanisms, Kunming Institute of Zoology, Chinese Academy of Sciences, Kunming, Yunnan, P.R. China
[3]National Resource Center for Non-Human Primates, Kunming Primate Research Center, and National Research Facility for Phenotypic & Genetic Analysis of Model Animals (Primate Facility), Kunming Institute of Zoology, Chinese Academy of Sciences, Kunming, Yunnan, P.R. China
[4]Kunming College of Life Science, University of Chinese Academy of Sciences, Kunming, Yunnan, P.R. China
[5]Center for Medical Genetics, Hunan Key Laboratory of Medical Genetics, MOE Key Lab of Rare Pediatric Diseases, School of Life Sciences, Central South University, Changsha, Hunan, P.R. China
[6]Department of Ecology, Evolution, and Marine Biology, University of California, Santa Barbara, Santa Barbara, CA, USA
[7]Department of Molecular, Cellular and Developmental Biology, University of California, Santa Barbara, Santa Barbara, CA, USA
[8]Neuroscience Research Institute, University of California, Santa Barbara, Santa Barbara, CA, USA
*Correspondence and requests for materials should be addressed to L.S. shilei@mail.kiz.ac.cn

This file contains Supplementary Figures 1-9.

Supplementary Figures

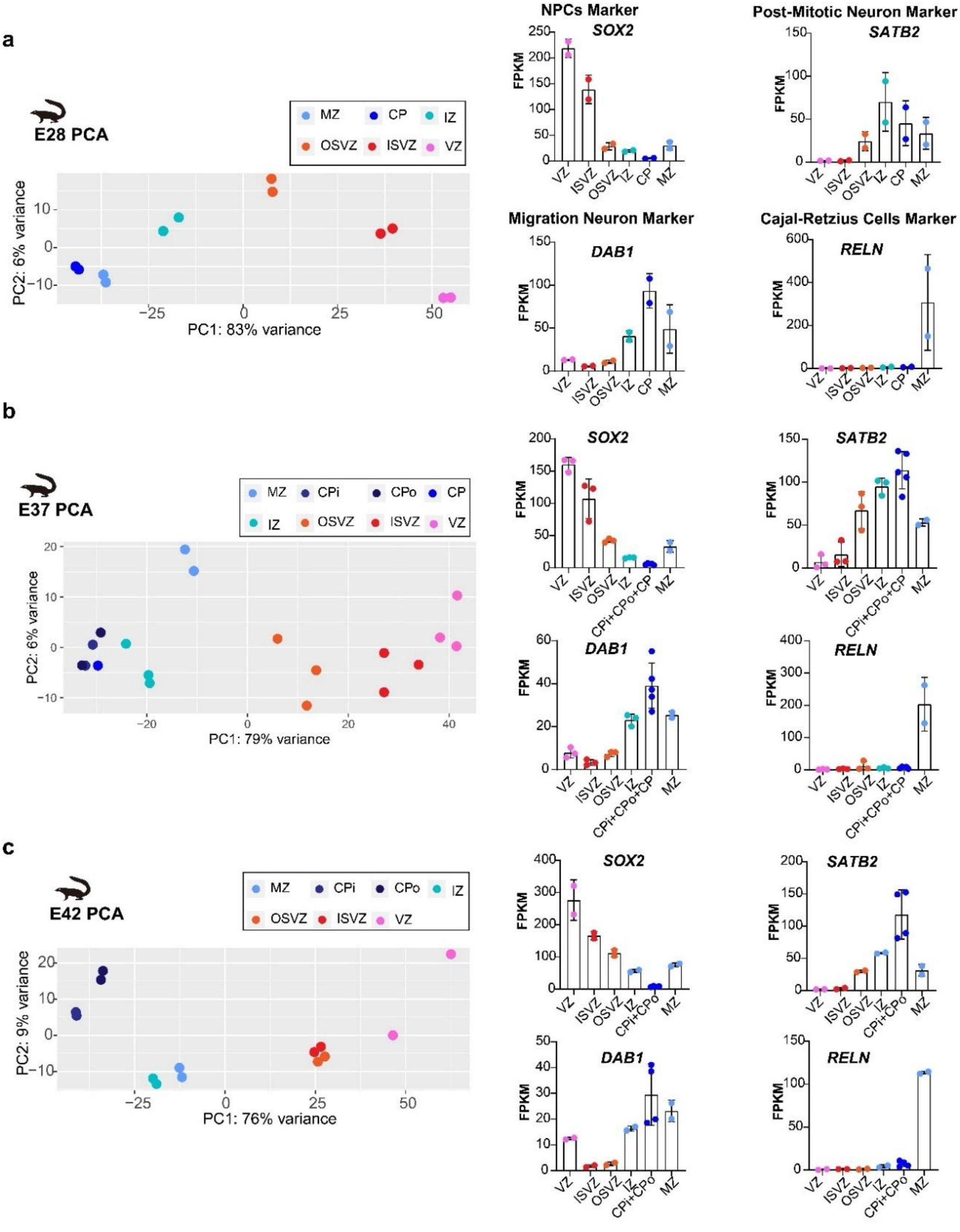

Fig. S1| Transcriptome analysis of cortical laminae in the Chinese tree shrew.

a–c, Left panel: PCA of frontal cortical layers from Chinese tree shrews at E28, E37, and E42 based on genome-wide gene expression levels. Right panel: marker gene analysis using *Sox2* for NPCs, *Satb2* for post-mitotic neurons, *DAB1* for migrating neurons, and *Reln* for Cajal-Retzius cells.

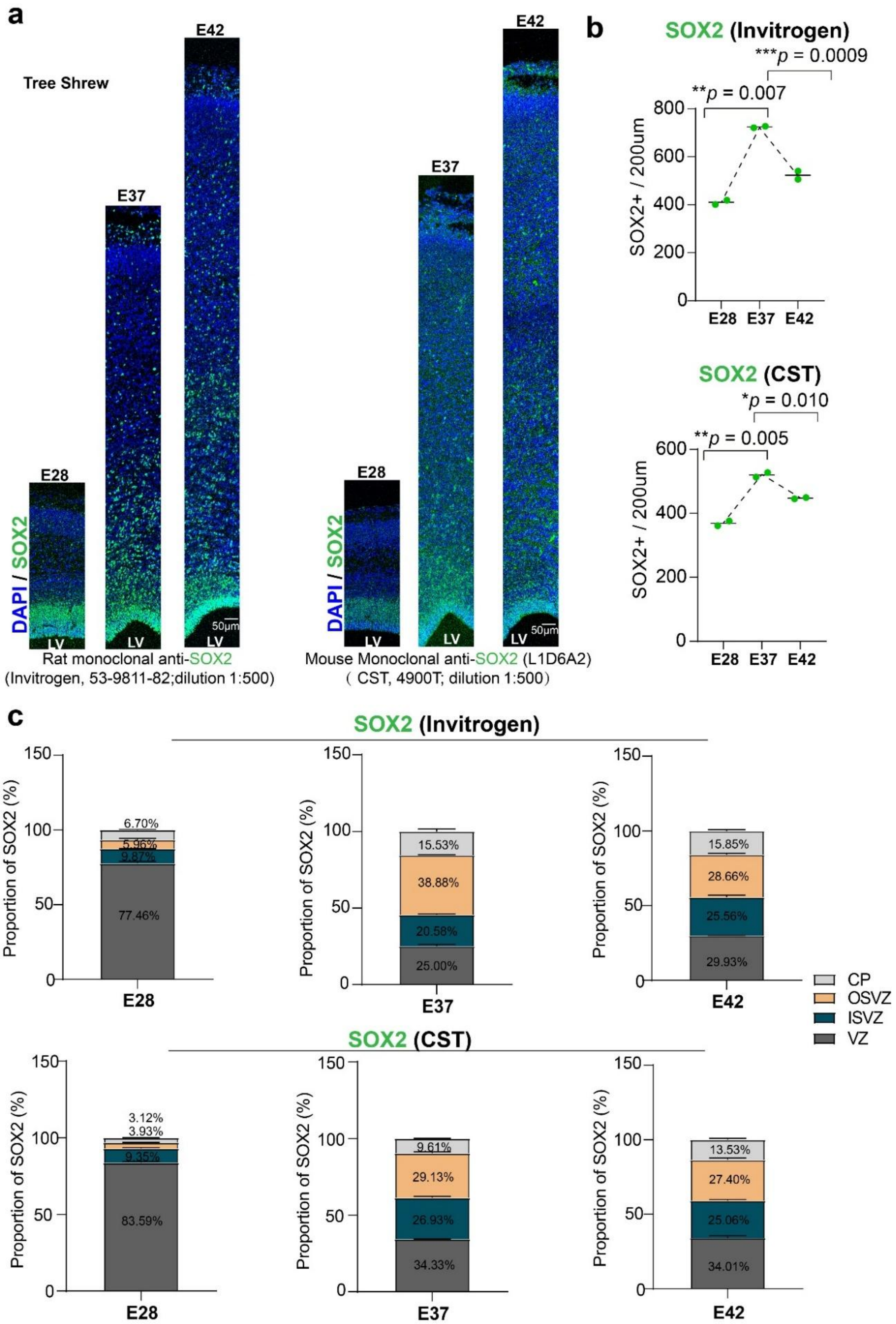

Fig. S2| SOX2 immunostaining comparisons between Invitrogen and CST SOX2 antibodies at E28, E37, and E42 in the cortical laminae of Chinese tree shrews.

a, Immunofluorescence staining of cortical sections for the *SOX2* using antibodies from Invitrogen and CST respectively, at E28, E37, and E42 in Chinese tree shrews.

b, Quantification of the distribution patterns of *SOX2* positive cells during cortical development. (E28, n = 2 or 3;E37, n = 2 ; E42, n = 2 , $*p < 0.05$, $**p < 0.01$, $***p < 0.001$, paired *t*-test).

c, Quantification of $SOX2^+$ cell numbers and proportions in the CP, VZ, ISVZ, and OSVZ at E28, E37 and E42. Two biological replicates were analyzed at each time point.

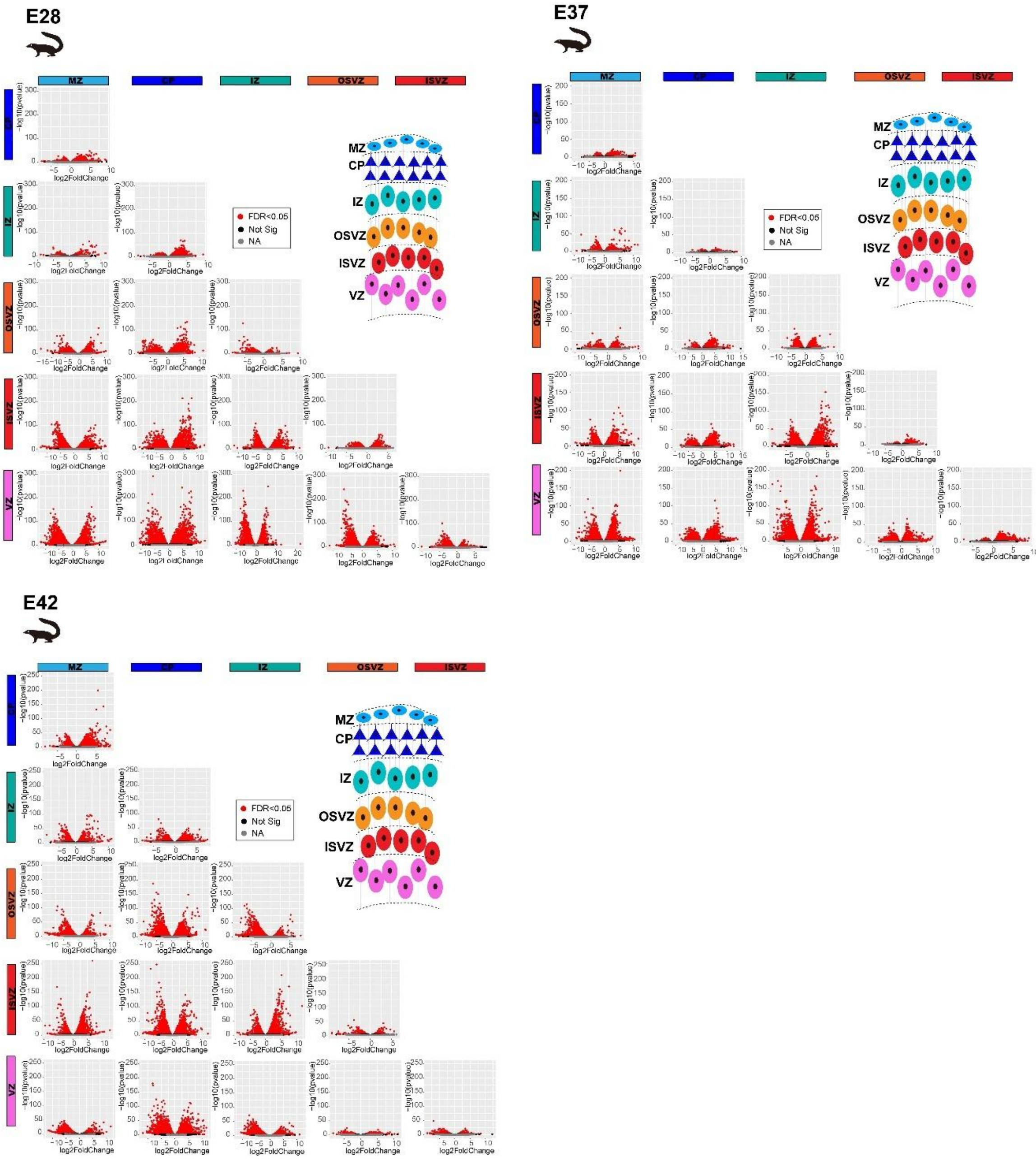

Fig. S3| Volcano plots demonstrating pairwise comparisons of gene expression between cortical laminae of Chinese tree shrews at E28, E37, and E42.

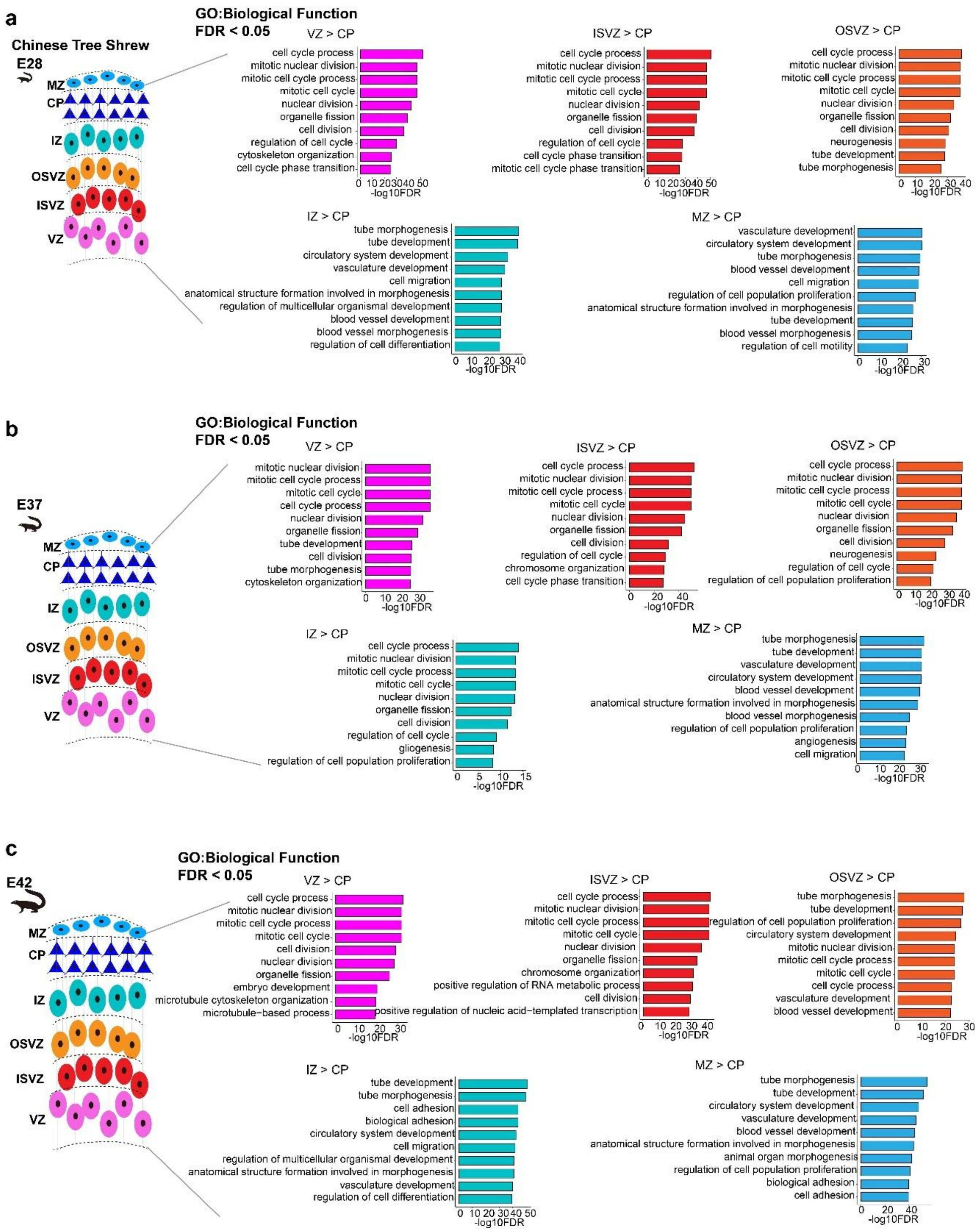

Fig. S4| GO analysis of genes upregulated in the VZ, ISVZ, OSVZ, IZ, and MZ relative to the CP at E28 (a), E37 (b), and E42 (c).

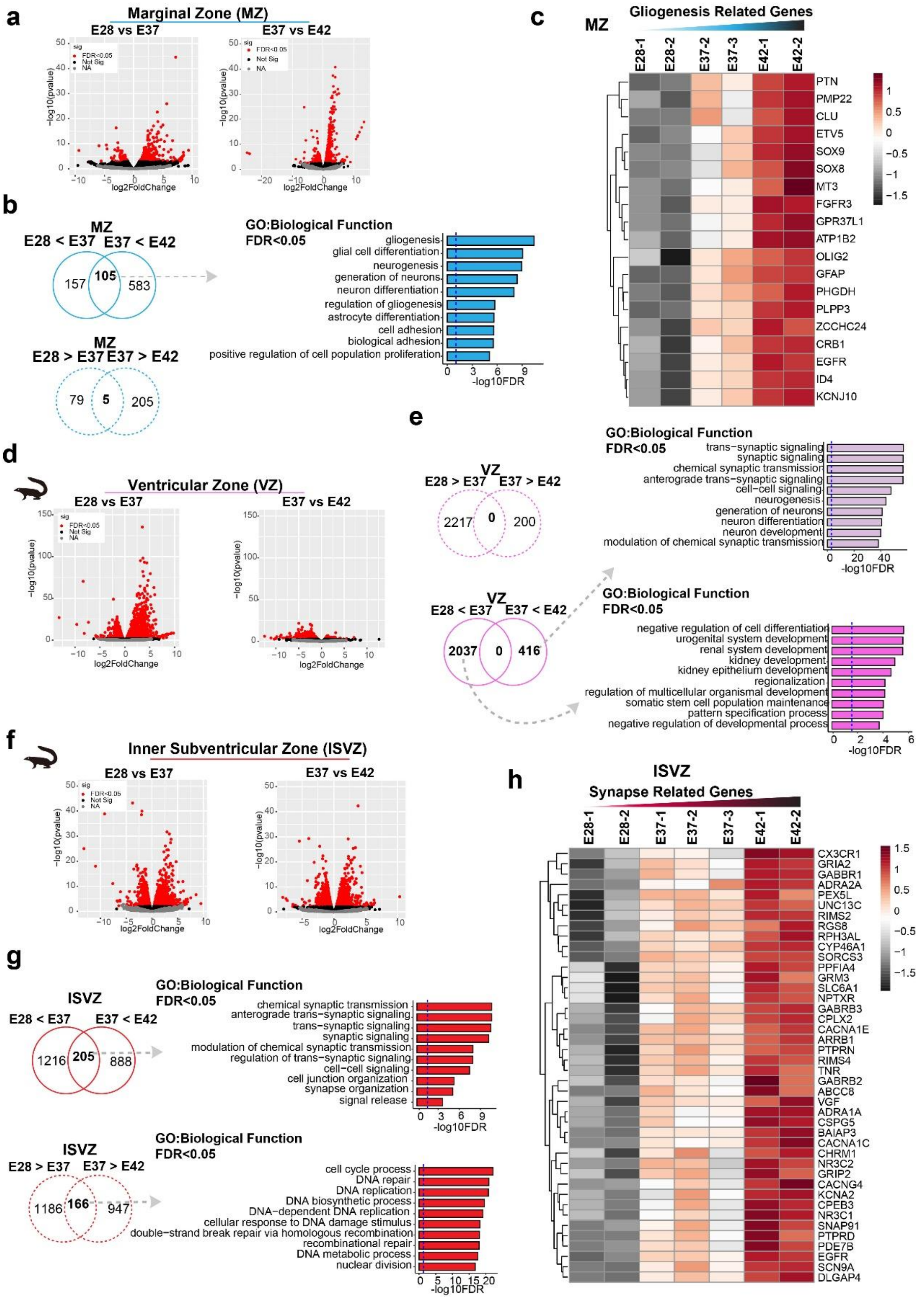

Fig. S5| Gliogenesis-related genes are progressively upregulated in the marginal zone (MZ) during tree shrew brain development.

a, Volcano plots showing DEGs between E28 and E37, and between E37 and E42, in the MZ.

b, Venn diagram plots (Top) showing that 105 genes exhibited consistently increased expression and were predominantly enriched in gliogenesis-associated pathways; (Bottom) only five genes showed decreased expression during cortical development. Note that the broken line marks the cutoff at -log10(FDR) = 1.301.

c, Hierarchical clustering showing the gradual upregulation of gliogenesis-related genes.

d, Volcano plots showing DEGs between E28 and E37, and between E37 and E42, in the ventricular zone.

e, (Left) Venn diagram plots showing that no genes exhibited consistently increased or decreased expression during cortical development. (Right) GO analysis of genes upregulated at E37 and E42, respectively. Note that the broken line marks the cutoff at -log10(FDR) = 1.301.

f, Volcano plots showing DEGs between E28 and E37, and between E37 and E42, in the inner subventricular zone.

g, Venn diagram plots (Top) showing that 205 genes exhibited consistently increased expression and were predominantly enriched in synaptic signaling–associated pathways; (Bottom) 166 cell cycle–related genes showed decreased expression during cortical development. Note that the broken line marks the cutoff at -log10(FDR) = 1.301.

h, Hierarchical clustering showing the gradual upregulation of synapse-related genes during ISVZ development in the tree shrew.

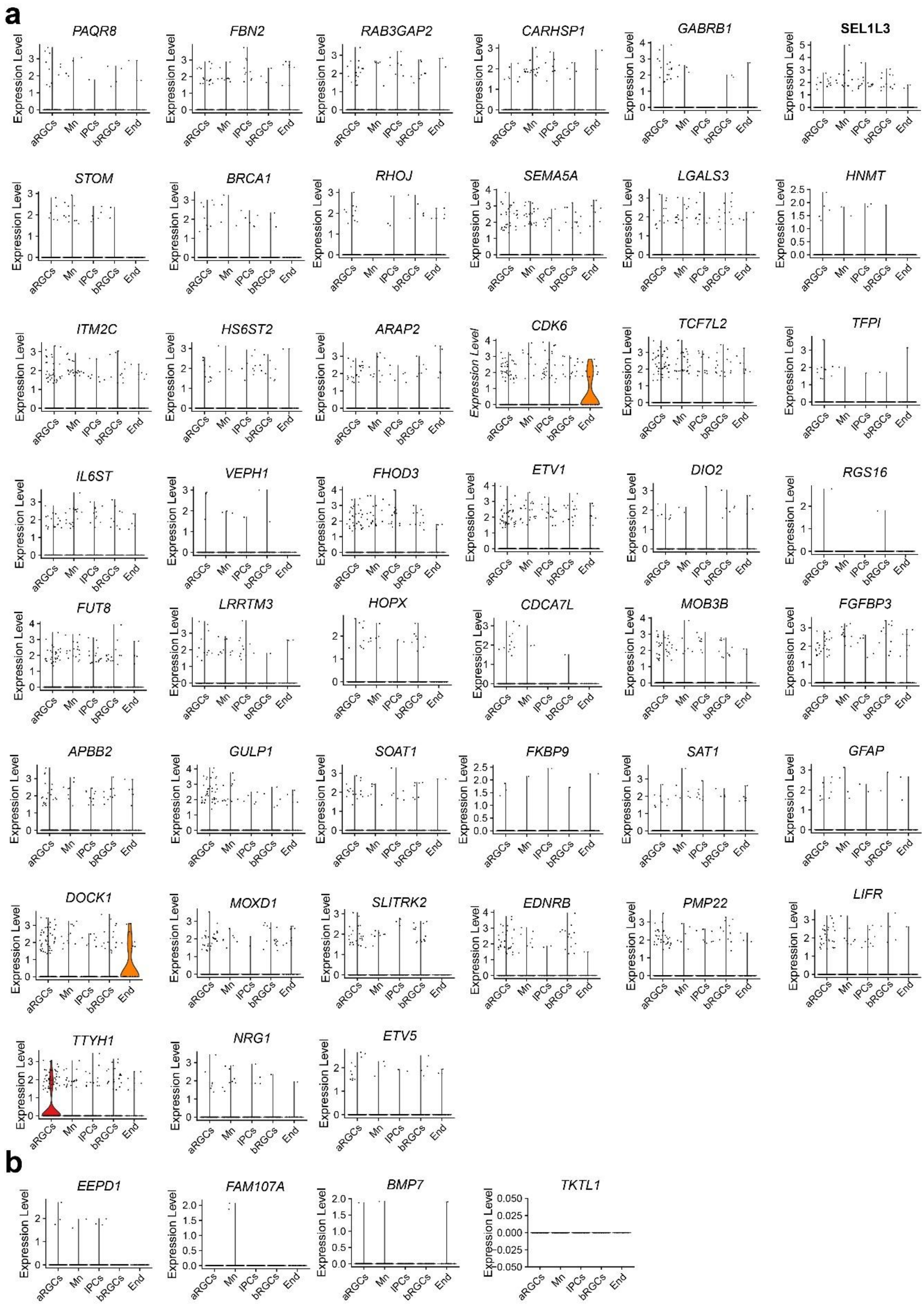

Fig. S6| Violin plots showing the expression patterns of oRGCs genes in the embryonic tree shrew cortex at E38.

a, Violin plots showing the expression patterns of 45 oRGCs genes.

b, Violin plots showing the expression patterns of another 4 oRG genes, which were not detected in bRGCs.

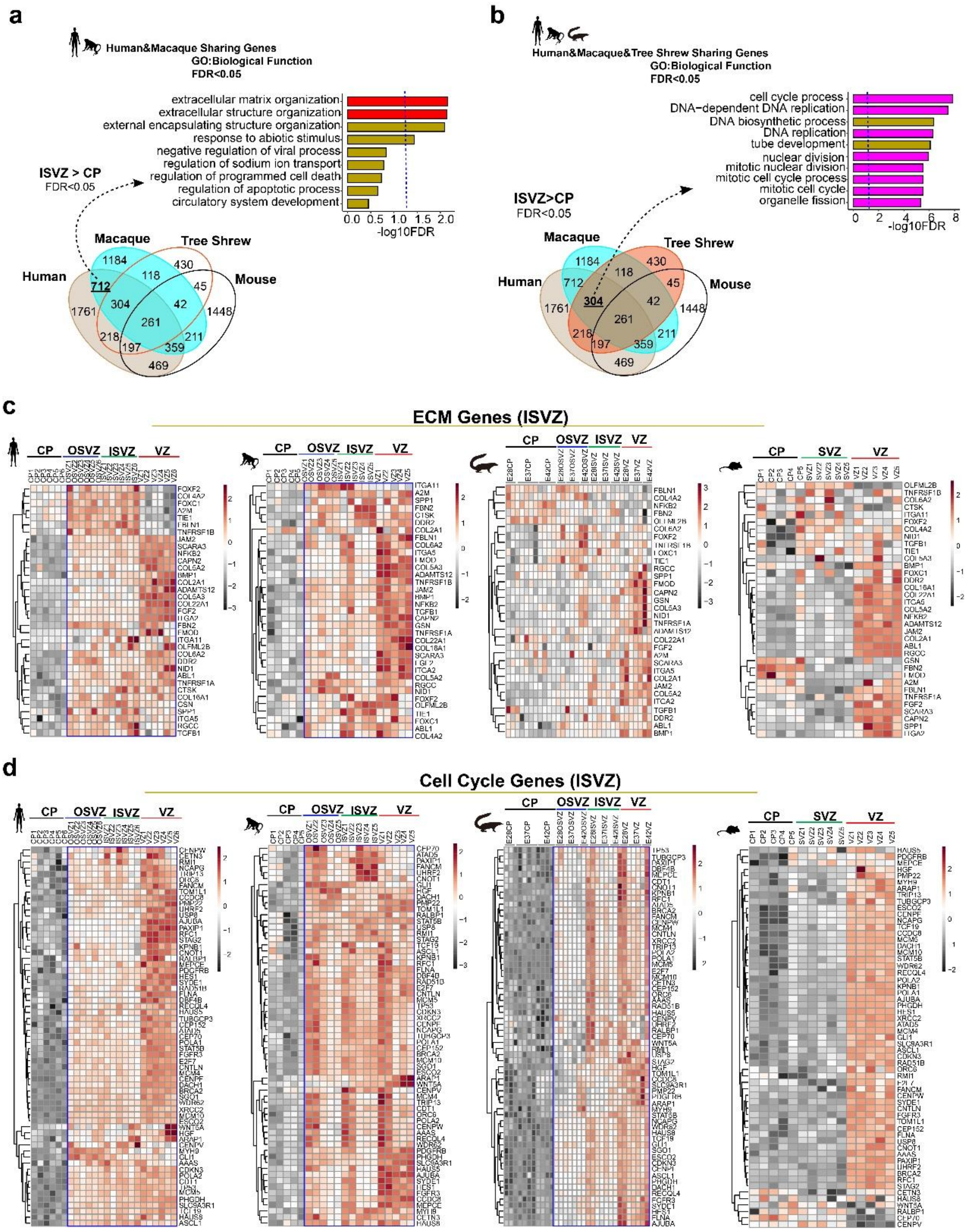

Fig. S7| Comparative analysis of ISVZ-expressed genes among mouse, tree shrew, monkey, and human species.

a, GO analysis of ISVZ-expressed genes shared between human and macaque. Note that the broken line marks the cutoff at -log10(FDR) = 1.301.

b, GO analysis of ISVZ-expressed genes shared among human, macaque, and tree shrew. Note that the broken line marks the cutoff at -log10(FDR) = 1.301.

c, Hierarchical clustering showing the expression patterns of ECM-associated genes in the ISVZ among mouse, tree shrew, monkey, and human species. ECM-associated genes in human and monkey were highlighted in a dashed box.

d, Hierarchical clustering showing the expression patterns of cell cycle–associated genes in the ISVZ among mouse, tree shrew, monkey, and human species. Cell-cycle-associated genes in human, monkey, and tree shrew were highlighted in a dashed box.

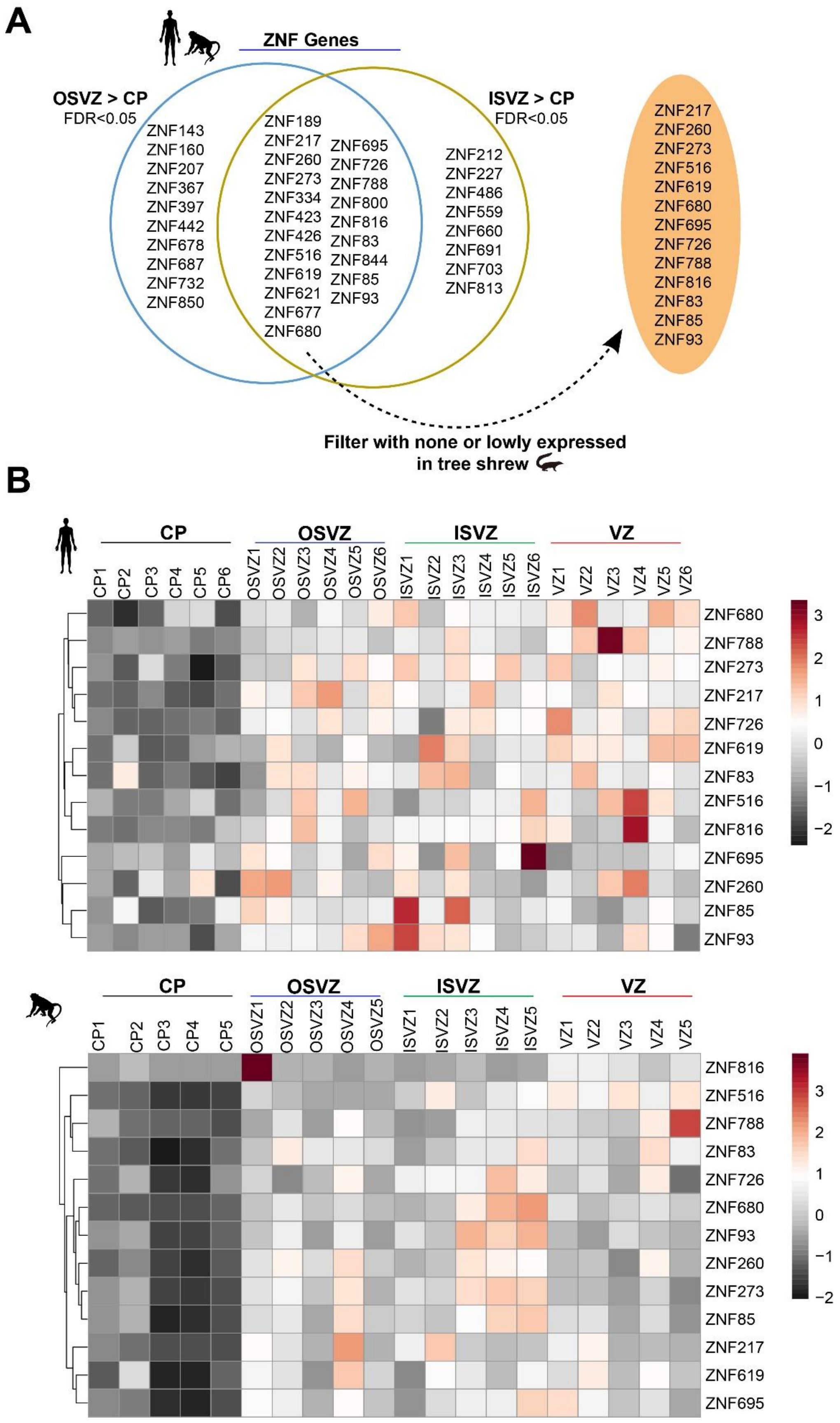

Fig. S8| Identification of ZNF genes expressed in the OSVZ and ISVZ of human and macaque.

a, Venny diagram plots showing ZNF genes shared between the OSVZ and ISVZ in human, and macaque.

b, Heatmap plots showing the expression patterns of ZNF genes in human and macaque, respectively.

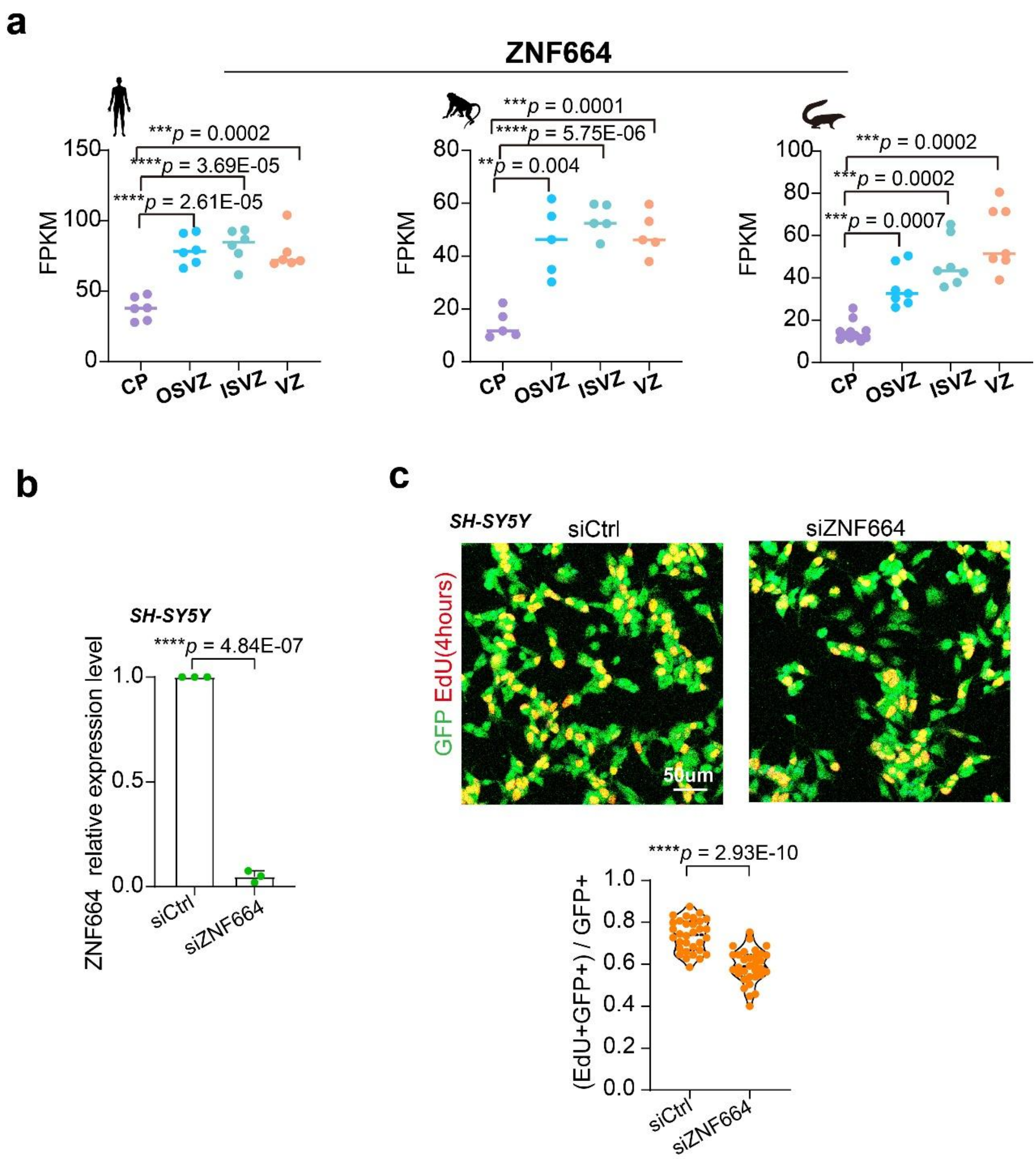

Fig. S9| Comparative expression analysis of *ZNF664* in the OSVZ and ISVZ across species.

a, Scatter plots demonstrating high expression of *ZNF664* in the VZ, ISVZ, and OSVZ compared with the CP in human, macaque, and tree shrew.

b, RNA quantification of *ZNF664* expression levels in *SH-SY5Y* cells.

c, (Top) Representative images of cells transfected siRNA with EdU pulses for 4 h. (Bottom) Violin plots of the percentages of $GFP^{+}EdU^{+}$ in GFP^{+} cells (n = 10 vision fields for total 3 replicates, data represented as median and quartile, $***p < 0.001$, unpaired *t*-test).
